# A Multilevel Redox-Based Prognostic Model for Asthma Severity: From Genotype to Serum Biomarkers

**DOI:** 10.3390/biomedicines14071509

**Published:** 2026-07-03

**Authors:** Shukur Wasman Smail, Rebaz Hamza Salih, Blnd Azad Ismail, Ivan Sdiq Maghdid, Raya Kh. Yashooa, Taban Kamal Rasheed, Shayma Hassan Hamadamin, Christer Janson

**Affiliations:** 1 College of Pharmacy, Cihan University-Erbil, Erbil, Kurdistan Region, Iraq; shukur.smail@su.edu.krd; 2 Department of Biology, College of Science, Salahaddin University-Erbil, Erbil 44001, Kurdistan Region, Iraq; blndazad698@gmail.com (B.A.I.); ivansdiq08@gmail.com (I.S.M.); taban.rasheed@su.edu.krd (T.K.R.); shine09876now@gmail.com (S.H.H.); 3 Department of Medical Science, Respiratory Medicine, and Allergology, Uppsala University and University Hospital, SE-751 85 Uppsala, Sweden; 4 Department of Respiratory Medicine, PAR Private Hospital, Erbil, Kurdistan Region, Iraq; rebaz.hamza1986@gmail.com; 5 Department of Genetics Unit, Bio Diagnostic Center (BDC), Erbil 44001, Kurdistan Region, Iraq; 6 Department of Biology, College of Education for Pure Sciences, University of Al-Hamdaniya, Mosul 41002, Iraq; raya.yashooa@uohamdaniya.edu.iq

**Keywords:** asthma severity, oxidative stress biomarkers, oxidant–antioxidant enzymes, genetic polymorphisms, cofactors, lipid peroxidation, microRNA, prognostic panels

## Abstract

Asthma is a heterogeneous chronic airway disease in which oxidative stress (OS) plays a central mechanistic role beyond classical immune-mediated inflammation. Reactive oxygen and nitrogen species (ROS/RNS), generated by recruited inflammatory cells and activated airway structural cells, drive epithelial injury, mucus hypersecretion, airway remodeling, and modulate key transcription factors including nuclear factor kappa B (NF-κB) and mitogen-activated protein kinase (MAPK) pathways. This review synthesizes current evidence on the multilevel redox-based determinants of asthma severity, spanning from genetic polymorphisms to circulating biomarkers. We examine serum antioxidant enzymes, superoxide dismutase (SOD), catalase (CAT), glutathione peroxidase (GPx), peroxiredoxins (PRDXs), and the thioredoxin (Trx) system as dynamic indicators of systemic redox status and disease severity, alongside oxidative enzymes including NADPH oxidases and dual oxidases (NOX/DUOX), xanthine oxidase (XO), and myeloperoxidase (MPO) that serve as upstream sources of airway oxidant burden. Functional genetic polymorphisms in antioxidant genes *(SOD2, CAT, glutathione S-transferase mu 1/glutathione S-transferase theta 1 (GSTM1/GSTT1), heme oxygenase-1 (HO-1), NAD(P)H quinone dehydrogenase 1 (NQO1), nuclear factor erythroid 2-related factor 2/Kelch-like ECH-associated protein 1 (Nrf2/KEAP1))* and oxidative enzyme genes including *nitric oxide synthase 1/2/3 (NOS1/2/3), MPO, cytochrome b-245 alpha chain (CYBA)*, and *xanthine dehydrogenase (XDH)* are reviewed as modulators of individual redox capacity and asthma susceptibility, with particular attention to gene–environment interactions. We further discuss oxidative damage biomarkers, including malondialdehyde (MDA), 8-isoprostanes, 4-hydroxynonenal, 8-oxo-7, 8-dihydro-2′-deoxyguanosine, protein carbonyls, 3-nitrotyrosine, and advanced oxidation protein products as indicators of lipid, DNA, and protein oxidation that correlate with disease activity and control. The roles of micronutrient cofactors in modulating antioxidant enzyme function and their potential as contextual biomarkers are also addressed. Additionally, emerging evidence on microRNAs (miRNAs) linked to OS biology in asthma is presented. Finally, we critically evaluate the challenges limiting clinical translation, including biomarker non-specificity, analytical variability, gene–environment complexity, and the absence of standardized reference ranges. This integrated framework supports the development of multilevel redox prognostic panels combining genetic, enzymatic, and oxidative damage readouts for improved asthma phenotyping, severity stratification, and personalized therapeutic approaches.

## 1. Introduction

Asthma is a heterogeneous chronic airway disorder characterized by variable airflow limitation, bronchial hyper responsiveness, and persistent airway inflammation. Beyond classic immune pathways, a large body of mechanistic and clinical evidence shows that asthma progression is tightly linked to oxidative stress (OS), where reactive oxygen/nitrogen species (ROS/RNS) are generated by recruited inflammatory cells and activated airway structural cells, contributing to epithelial injury, mucus hypersecretion, and remodeling. Importantly, ROS/RNS are not only toxic by-products; they also function as redox signaling mediators that trigger key transcription factors and kinases, such as nuclear factor kappa B (NF-κB) and mitogen-activated protein kinases (MAPK). These pathways modulate immune-cell activation, macrophage polarization, and inflammatory gene expression, thereby sustaining disease activity and influencing severity [1].

A defining feature of redox imbalance in asthma is the mismatch between oxidant production systems and endogenous antioxidant defenses. Enzymatic oxidant sources in the airway epithelium and immune cells drive persistent oxidant pressure, while antioxidant enzymes (e.g., superoxide dismutase (SOD), catalase (CAT), glutathione-dependent enzymes) may be quantitatively reduced, functionally impaired, or overwhelmed during active disease and exacerbations. Pediatric and adult-focused syntheses consistently emphasize that OS rises with disease activity and may be particularly relevant in severe phenotypes and in patients exposed to environmental oxidants such as air pollution and tobacco smoke [2].

Because OS is mechanistically embedded in airway pathology, oxidant/antioxidant readouts have been investigated as biomarkers for asthma activity and severity. Among the most studied noninvasive markers, exhaled breath condensate (EBC) 8-isoprostane (a lipid peroxidation product) is repeatedly elevated in asthma and has been evaluated in systematic review/meta-analysis as a candidate biomarker in adults. However, these analyses also highlight substantial methodological variability and between-subject heterogeneity, supporting the concept that oxidative biomarkers are most informative when used as part of a panel rather than as single standalone tests. Such a panel might ideally integrate non-invasive markers of lipid peroxidation (e.g., EBC 8-isoprostane), systemic enzymatic activity (e.g., serum SOD or glutathione peroxidase (GPx)), and high-impact genetic variants (e.g., *glutathione S-transferase mu 1/theta 1 null* (*GSTM1/GSTT1* null) or *CAT* rs1001179) to provide a more comprehensive redox profile [3]. Efforts to establish reference ranges for EBC 8-isoprostane further underscore the need for standardized methods to enable clinical translation [4].

Inter-individual variation in OS profiles is not explained by inflammation alone; it is also shaped by genetic differences in oxidative and antioxidative enzymes. Detoxification and antioxidant gene polymorphisms can shift baseline redox capacity and modify susceptibility to oxidant-driven airway injury. Among the most consistently studied, *GSTM1-null and GSTT1-null* genotypes reflecting the deletion of key GST enzymes have been evaluated in updated meta-analyses, supporting their association with asthma risk and providing biological plausibility for higher oxidative burden under environmental oxidant exposure. This genetic layer is central to understanding why some patients exhibit disproportionate OS, more severe disease trajectories, or exposure-sensitive exacerbations despite similar clinical diagnoses [5].

Redox biology also helps explain one of the most clinically important problems in difficult asthma: reduced responsiveness to corticosteroids. OS can suppress corticosteroid efficacy by disrupting epigenetic and signaling mechanisms required for steroid-mediated repression of inflammatory genes, including reductions in *histone deacetylase 2 (HDAC2)* activity/expression. Reviews and mechanistic studies demonstrate that OS can diminish HDAC2 function and thereby promote steroid resistance, while additional work shows that loss of antioxidant regulatory control (e.g., nuclear factor erythroid 2-related factor 2 (Nrf2) deficiency) is linked to decreased HDAC2 and steroid-insensitive inflammatory responses. These findings elevate redox regulatory pathways from “background mechanisms” to clinically relevant predictors of treatment response in severe asthma [6].

Taken together, current evidence supports an integrated model in which asthma severity reflects (i) upstream oxidant enzyme activity, (ii) downstream antioxidant enzyme capacity, (iii) inherited variation in detoxification and antioxidant genes, and (iv) redox regulatory networks that shape inflammation and steroid responsiveness. This provides the rationale for focusing on oxidative/antioxidative polymorphisms and enzyme biomarkers as prognostic tools and mechanistic indicators. Accordingly, this review synthesizes the evidence on oxidative and antioxidative enzyme biomarkers, highlights major genetic polymorphisms affecting redox balance, and discusses how regulatory networks (notably Nrf2- and HDAC2-linked mechanisms) may help stratify asthma phenotypes and predict severity and therapeutic response.

## 2. Asthma and Oxidative Stress

### 2.1. Introduction to Asthma, Its Types, Management

Asthma is a common, heterogeneous, chronic respiratory disease, usually characterized by airway inflammation and variable respiratory symptoms, such as wheeze, shortness of breath, chest tightness, and cough, that fluctuate over time in frequency and intensity [7]. These symptoms are associated with variable expiratory airflow limitation caused by episodic bronchoconstriction, airway wall thickening, and increased mucus, and in long-standing disease, the airflow limitation may become partly persistent [8]. Clinically, asthma is recognized by symptom patterns that often worsen at night or on waking and are commonly triggered by exercise [9], laughter, allergens, cold air, or viral respiratory infections [10]. Because symptoms and airflow limitation can be intermittent, physical examination may be normal between episodes, with wheeze sometimes detectable only during forced expiration or during symptomatic periods [11].

**Figure 1:**
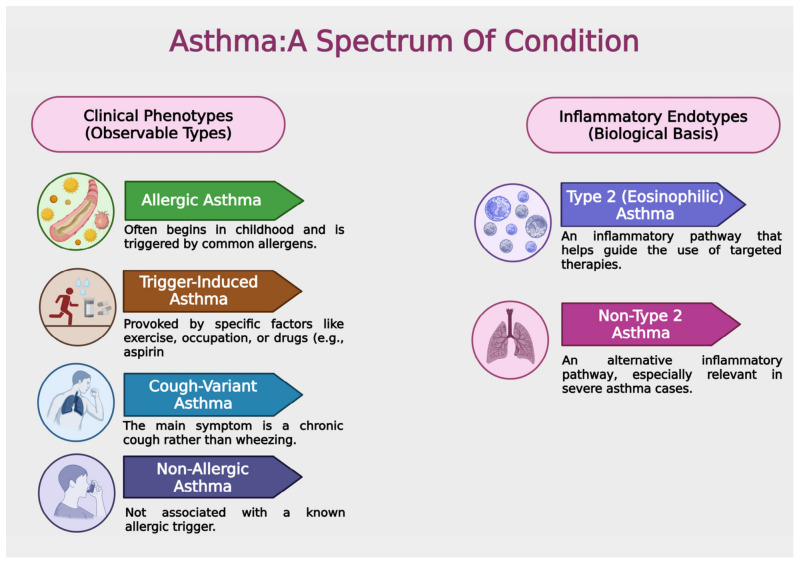
Asthma: A Spectrum of Condition. The figure illustrates the clinical phenotypes (observable types) and inflammatory endotypes (biological basis) of asthma. Clinical phenotypes include: allergic asthma (often triggered by common allergens), trigger-induced asthma (provoked by specific factors such as exercise, occupation, or drugs), cough-variant asthma (where chronic cough is the primary symptom), and non-allergic asthma (not associated with a known allergic trigger). Inflammatory endotypes include: Type 2 (Eosinophilic) asthma (an inflammatory pathway linked to eosinophilic activation and targeted therapies), and non-type 2 asthma (an alternative inflammatory pathway, especially relevant in severe cases). The image was created using the BioRender program, available at https://www.biorender.com.

Asthma is not a single uniform condition; it has multiple phenotypes (types) (Figure 1) reflecting different clinical patterns and underlying biological mechanisms. Widely recognized clinical types include allergic asthma (often beginning in childhood), non-allergic asthma, exercise-induced bronchoconstriction, occupational asthma (and work-aggravated asthma), and cough-variant asthma, in which chronic cough may be the predominant symptom, and airway hyper responsiveness is typically demonstrated by provocation testing [11,12,13]. Drug-triggered phenotypes are also important, including asthma exacerbations provoked by beta-blockers, and in susceptible individuals, aspirin/NSAIDs. In contemporary frameworks, severe asthma is also frequently described by inflammatory endotypes such as type 2 (eosinophilic) asthma versus non-type 2 asthma, which can guide biomarker use and targeted therapy approaches [14,15,16].

International guidance emphasizes that an asthma diagnosis should be confirmed and documented using objective evidence, not only symptoms. The core diagnostic requirement is a history of typical variable respiratory symptoms together with evidence of variable expiratory airflow limitation (current or previous) [17,18]. Objective confirmation is most commonly obtained with spirometry demonstrating significant bronchodilator responsiveness (reversibility). In adults, an increase in forced expiratory volume in one second (FEV_1_) or forced vital capacity (FVC) ≥12% and ≥200 mL from the pre-bronchodilator value supports asthma; when spirometry is not available, a peak expiratory flow (PEF) increase ≥20% can support variability [18,19]. Additional objective evidence includes excess diurnal PEF variability (e.g., >10% in adults; higher thresholds are used in children) and a clinically meaningful improvement in lung function after a trial of anti-inflammatory therapy (e.g., improvement in FEV_1_ after several weeks of inhaled corticosteroids (ICS)-containing treatment, outside respiratory infections) [17,20]. Testing may need to be repeated during symptomatic periods or after withholding bronchodilators to demonstrate variability.

When routine spirometry does not confirm variability despite typical symptoms, guidelines support using additional tests, most importantly, bronchial provocation (challenge) testing to detect airway hyper responsiveness, and structured assessment in special contexts (e.g., suspected occupational asthma) [21,22]. Fractional exhaled nitric oxide (FeNO) is a non-invasive biomarker that quantifies the concentration of nitric oxide in breath that has been exhaled [23], and blood eosinophils can support a diagnosis of type 2 asthma in a symptomatic patient, but low values do not exclude asthma [24].

#### Asthma Severity

Asthma severity is a multidimensional, dynamic construct that reflects both the intrinsic intensity of the underlying disease and its responsiveness to treatment, and may change over months to years in the same individual. In the present review, ‘asthma severity’ is used in accordance with the Global Initiative for Asthma (GINA) and the joint European Respiratory Society/American Thoracic Society (ERS/ATS) guidelines [25], and is operationalized through four complementary instruments as reported across the primary studies cited herein: (1) objective lung function, principally forced expiratory volume in one second as a percentage of predicted (FEV_1_ % predicted), where values below 60%, 60–80%, and above 80% broadly correspond to severe, moderate, and mild airflow limitation, respectively [25]; (2) exacerbation frequency, defined as ≥2 moderate or ≥1 severe episode per year requiring systemic corticosteroids, emergency department attendance, or hospitalization [26]; (3) validated patient-reported symptom-control scores, including the Asthma Control Test (ACT, score ≤ 19 indicating uncontrolled disease) and the Asthma Control Questionnaire (ACQ, score ≥ 1.5 indicating poor control), which are moderately correlated with spirometry but capture additional clinical dimensions not reflected by lung function alone [27,28]; and (4) GINA treatment step, where ‘severe asthma’ specifically denotes asthma that requires, or remains uncontrolled despite, high-dose ICS plus a second controller agent (GINA Steps 4–5) [25]. Because classification based solely on symptoms or spirometry may not fully reflect a patient’s true disease burden, particularly in those already receiving controller therapy [29], and because successive GINA updates since 2014 have shifted emphasis from initial severity grading toward ongoing control assessment as the primary basis for treatment decisions [30], the term ‘severity’ throughout this review describes the overall degree of disease burden, encompassing both current clinical impairment and future risk of adverse outcomes. Where a specific severity measure was used in a cited primary study, this is noted explicitly in the text.

### 2.2. Interaction of Immune System, Oxidative Stress, and Redox Signaling and Inflammation

OS is best understood within modern redox biology as a disturbance of controlled redox homeostasis rather than simply “too many oxidants” [31]. Aerobic life operates in a dynamic redox steady state in which oxidant production and antioxidant defenses are balanced, and oxidants are also used as signals [32]. When this balance is shifted in a sustained or mis-localized manner, OS can disrupt redox signaling/control and/or lead to molecular injury, thereby promoting pathological inflammation [33,34]. This mechanistic view is emphasized in foundational redox frameworks that redefine OS as the disruption of redox signaling and control, and expand the concept to include both adaptive (physiologic) and damaging (pathologic) outcomes [31].

**Figure 2:**
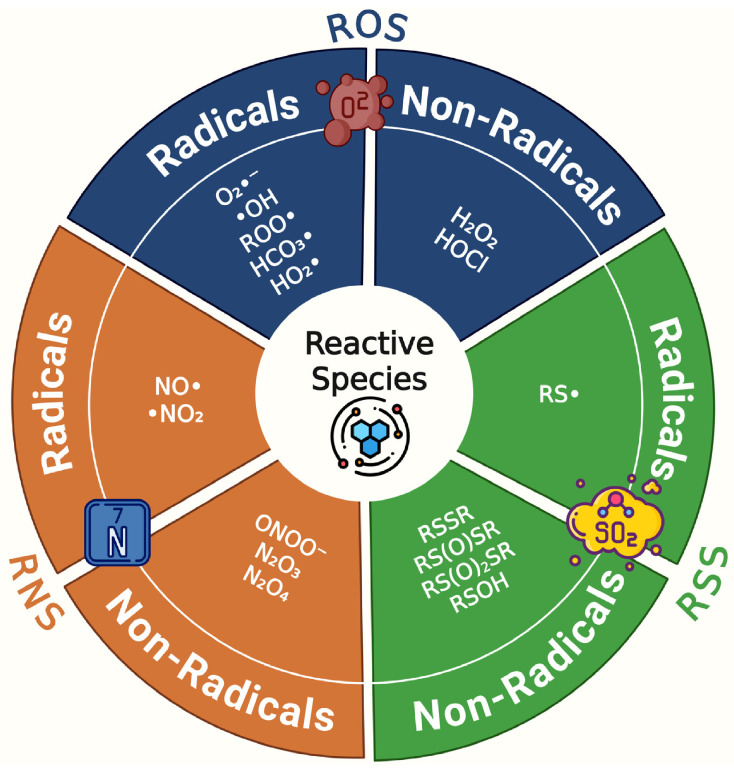
Overview of Reactive Species. This schematic categorizes reactive species into three main groups: reactive oxygen species (ROS), reactive nitrogen species (RNS), and reactive sulfur species (RSS). ROS are further divided into radicals (e.g., O_2_•^−^, OH•) and non-radicals (e.g., H_2_O_2_, HOCl). RNS include radicals like NO• and NO_2_•, and non-radicals such as ONOO^−^ and N_2_O_3_. RSS contain radicals like RS• and non-radicals such as RSSR, RSO_2_SR, and RSOH. These species are involved in various biochemical processes and can contribute to cellular damage under certain conditions. The image was created using the BioRender platform. ROS: reactive oxygen species, RNS: reactive nitrogen species, RSS: reactive sulfur species, RS: reactive species, NO: nitric oxide, NO_2_: nitrogen dioxide, ONOO^−^: peroxynitrite, N_2_O_3_: dinitrogen trioxide, O_2_•^−^: superoxide, OH•: hydroxyl radical, H_2_O_2_: hydrogen peroxide, HOCl: hypochlorous acid, HCO_3_•: bicarbonate, H_2_O: water, RSSR: disulfide, RSO_2_SR: sulfonate, RSOH: sulfhydryl. The image was created using the BioRender program, available at https://www.biorender.com.

Biologically relevant oxidants include ROS and RNS (Figure 2). ROS include radical species such as superoxide (O_2_•^−^) and hydroxyl radical (•OH), and non-radical but highly reactive species such as hydrogen peroxide (H_2_O_2_) and hypochlorous acid (HOCl) [35,36,37]. RNS include nitric oxide (NO•) and peroxynitrite (ONOO^−^), the latter formed rapidly by the diffusion-limited reaction between NO• and O_2_•^−^, linking oxidative and nitrosative pathways in inflamed tissues [38]. These reactive species arise from multiple sources, including mitochondrial electron transport, enzyme systems such as NADPH oxidases (NOX), and inducible NO synthase (iNOS) during immune activation [39].

**Figure 3:**
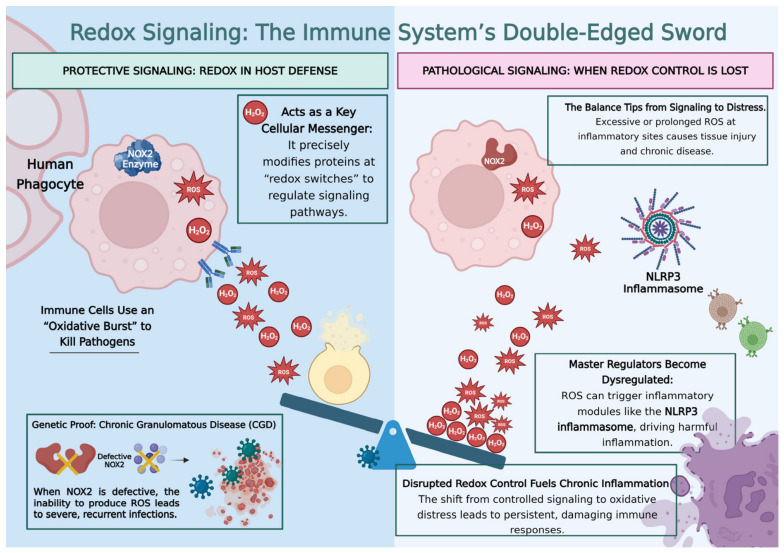
Redox Signaling: The Immune System’s Double-Edged Sword. This figure illustrates the two contrasting roles of redox signaling in the immune system. On the left, Protective Signaling: Redox in Host Defense, depicts how immune cells utilize an “oxidative burst” to produce reactive oxygen species (ROS) like H_2_O_2_ to kill pathogens. The enzyme NOX2 plays a crucial role by acting as a key cellular messenger, modifying proteins at “redox switches” to regulate signaling pathways. This process is essential for efficient immune defense, and genetic proof from chronic granulomatous disease (CGD) shows that defects in NOX2 lead to a compromised ability to produce ROS, resulting in recurrent infections. On the right, Pathological Signaling: When Redox Control is Lost, shows how excessive or prolonged ROS at inflammatory sites, caused by dysregulated redox control, leads to tissue injury and chronic diseases. The imbalance in redox signaling can trigger the NOD-like receptor family pyrin domain containing 3 (NLRP3) inflammasome, a master regulator of inflammation, contributing to harmful inflammation and disease progression. ROS: reactive oxygen species, H_2_O_2_: hydrogen peroxide, NOX2: NADPH oxidase 2, NLRP3: NOD-like receptor family pyrin domain containing 3. The image was created using the BioRender program, available at https://www.biorender.com.

In redox signaling, as demonstrated in Figure 3, oxidants are not merely toxic by-products; they can function as regulated second messengers. Among the ROS, H_2_O_2_ most closely fits the “second messenger” requirements because it can be produced and removed enzymatically and can oxidize selected protein thiols in a spatially and temporally controlled way [40,41]. Redox signaling commonly occurs through the reversible oxidation of cysteine residues (“redox switches”), forming modifications such as sulfenic acids and disulfides that modulate protein conformation, enzyme activity, and downstream pathway activation. This chemistry underpins how cells translate oxidant changes into changes in phosphorylation cascades, transcription, metabolism, and immune responses [42,43].

A major point of intersection between redox signaling and inflammation is the reversible oxidation of protein tyrosine phosphatases (PTPs). PTPs contain catalytic cysteines that are particularly sensitive to oxidation by H_2_O_2_; transient PTP inactivation can amplify receptor-driven kinase signaling and shape inflammatory outputs [44]. This provides a mechanistic link between local oxidant generation and classical inflammatory pathways, helping explain how oxidants can enhance signaling without immediate widespread macromolecular damage until redox control is lost and oxidative distress develops [45].

The immune system both generates and responds to ROS/RNS. In innate immunity, phagocytes use NADPH oxidase 2 (NOX2) to produce ROS during the oxidative burst, supporting microbial killing and shaping immune signaling [46]. Human genetics strongly supports this role: loss-of-function defects in NOX2 components cause chronic granulomatous disease (CGD) with impaired oxidative burst and recurrent severe infections, illustrating how redox enzymes are integral to host defense and immune regulation [47]. At the same time, immune-derived ROS can act as signaling cues that influence cytokine production, leukocyte recruitment, and endothelial activation; excessive or prolonged ROS generation at inflammatory sites contributes to endothelial dysfunction, tissue injury, and chronic inflammatory disease progression [48,49].

Inflammatory signaling networks are also governed by redox-sensitive transcriptional and innate immune modules. Nrf2 is a master regulator of antioxidant and cytoprotective gene programs and has been shown to modulate innate immunity through interactions with Toll-like receptor (TLR)–NF-κB signaling, inflammasome pathways, and interferon responses, positioning redox homeostasis as a regulator of inflammatory tone [50,51]. In parallel, ROS are repeatedly implicated as triggers and effectors in NOD-like receptor family pyrin domain containing 3 (NLRP3) inflammasome activation, where spatiotemporal ROS signals can contribute to interleukin-1 beta (IL-1β)-driven inflammation and pathology when dysregulated. Together, these mechanisms support a unified model in which redox signaling is essential for effective immunity, but the disruption of redox control shifts responses toward persistent inflammation and tissue damage [52,53].

## 3. Pathophysiology of Oxidative Stress in Asthma

**Figure 4:**
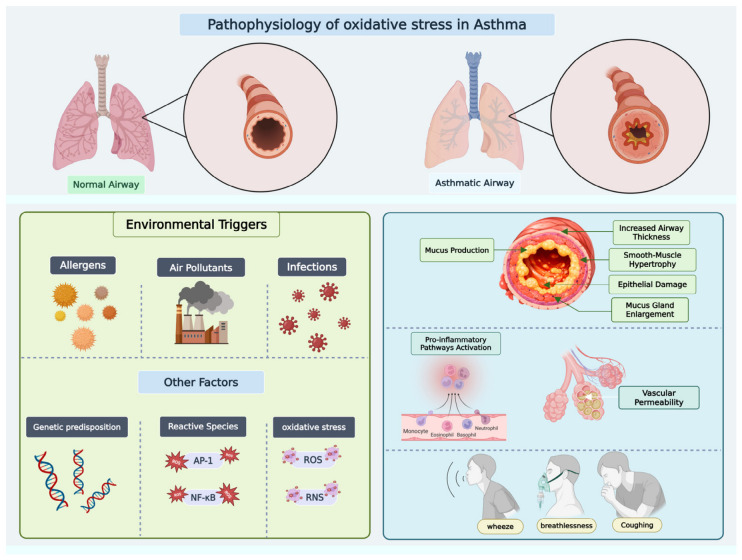
Pathophysiology of OS in Asthma. This figure compares a normal airway with an asthmatic airway, highlighting the impact of OS. Environmental triggers, including allergens, air pollutants, and infections, contribute to the exacerbation of asthma. Other factors such as genetic predisposition, reactive species (e.g., ROS and RNS), and OS play a critical role in activating pro-inflammatory pathways like AP-1 and NF-κB. In asthmatic airways, this leads to increased airway thickness, mucus production, smooth muscle hypertrophy, and epithelial damage. The resulting inflammation causes symptoms such as wheezing, breathlessness, and coughing. ROS: reactive oxygen species, RNS: reactive nitrogen species, AP-1: activator protein 1, NF-κB: nuclear factor kappa B. The image was created using the BioRender program, available at https://www.biorender.com.

The structure of the airway is altered in asthma—there is epithelial damage, subepithelial fibrosis, smooth-muscle hypertrophy, mucus gland enlargement, and increased airway wall thickness—all contributing to the hallmark symptoms of wheeze, cough, and breathlessness [54] (Figure 4). Asthma is not a single disease entity but rather a heterogeneous group of conditions, leading to the concept of various phenotypes and endotypes. Phenotypes refer to observable clinical features such as allergic (atopic) asthma, non-allergic asthma, late-onset asthma, obesity-associated asthma, and exacerbation-prone asthma, while endotypes are defined by distinct underlying pathophysiological mechanisms and molecular pathways [12]. Oxidative and nitrosative stress are seen as critical factors in the pathophysiology of asthma, rather than as universal primary mechanisms applicable to all endotypes. Excessive production of ROS and RNS can exacerbate airway inflammation, damage airway epithelial cells, elevate mucus output, facilitate airway remodeling, and impair pulmonary function [55]. Furthermore, OS has been linked to the emergence of severe asthma phenotypes that exhibit resistance to corticosteroids. Consequently, oxidative/nitrosative stress must be considered a significant pathogenic element contributing to the interplay of other processes in the pathogenesis and progression of asthma [56]. Across multiple endotypes, a key contributing mechanism is oxidative and nitrosative stress, which occurs when the production of ROS/RNS exceeds the antioxidant defenses. These reactive species contribute to airway inflammation, trigger redox-sensitive transcription factors such as NF-κB and activator protein 1 (AP-1), damage cellular macromolecules (lipids, proteins, DNA), and thereby perpetuate airway hyper responsiveness and remodeling [2].

The impact of OS in asthma is particularly significant when considering airway remodeling, treatment responsiveness, and disease severity. ROS and RNS lead to epithelial barrier dysfunction, increased mucus production, vascular permeability, and activation of pro-inflammatory pathways [54]. Additionally, antioxidant defenses are often reduced in asthma, which exacerbates the imbalance and may contribute to phenotypes that are less responsive to standard therapies (for instance, in T2-low or obesity-related asthma) [2].

## 4. Serum Activity of Pro-Oxidant and Antioxidant Enzymes as Biomarkers of Asthma Severity

Asthma is now recognized as a complex chronic inflammatory airway disease that extends beyond simple smooth muscle dysfunction. Increasing evidence indicates that OS contributes substantially to disease pathogenesis, airway remodeling, and progression by disrupting the balance between ROS production and endogenous antioxidant defenses [57]. This OS arises when ROS production exceeds endogenous antioxidant defenses. Central to this defense system are key serum enzymes, including SOD and CAT, which serve as the main line of protection against O_2_•^−^ and H_2_O_2_ [54].

The “imbalance theory” pathophysiological foundation depends on the permanent activation of immune cells that enter the body through its borders because allergens and environmental triggers lead them to bronchial tissues. The cells perform an immediate metabolic reaction called an “oxidative burst”, which results in their oxygen uptake rate increasing suddenly. This process is largely mediated by the membrane-bound enzyme NOX2, which catalyzes the conversion of molecular oxygen into superoxide anions (O_2_^•−^) [58].

Following this initial burst, the reactive species undergo conversion to H_2_O_2_ and to highly reactive HOCl when neutrophilic myeloperoxidase (MPO) is present [59]. The excessive and continuous release of ROS from asthmatic airways causes severe damage to all surrounding tissues because these ROS from healthy immune functions serve protective functions. The localized oxidative surge causes damage to the respiratory epithelium while it depletes the body’s antioxidant protection systems. Clinical evidence suggests that as these pulmonary inflammatory cells remain active, there is a concomitant depletion of SOD and CAT, as the body’s enzymatic buffering capacity is overwhelmed by the mounting oxidative load [54]. The evidence indicates that persistent activation of pulmonary inflammatory cells generates excessive ROS, resulting in OS. Chronic oxidative and inflammatory signals at the transcriptional level may compromise antioxidant defense mechanisms, particularly by disrupting the Nrf2 signaling pathway, resulting in the reduced expression of antioxidant enzymes, including SOD and CAT [60]. Furthermore, pro-inflammatory cytokines including tumor necrosis factor-alpha (TNF-α) and IL-1β may impede the transcription of antioxidant genes [61]. Excessive ROS can directly oxidize and inactivate SOD and CAT proteins at the enzymatic level, alter their active sites, and accelerate the degradation of SOD and CAT proteins. Consequently, the reduced gene expression and enzymatic activity result in diminished levels of SOD and CAT, thus compromising the endogenous antioxidant defense system and exacerbating oxidative damage in pulmonary tissues [62].

### 4.1. Serum Activity of Antioxidant Enzymes

**Figure 5:**
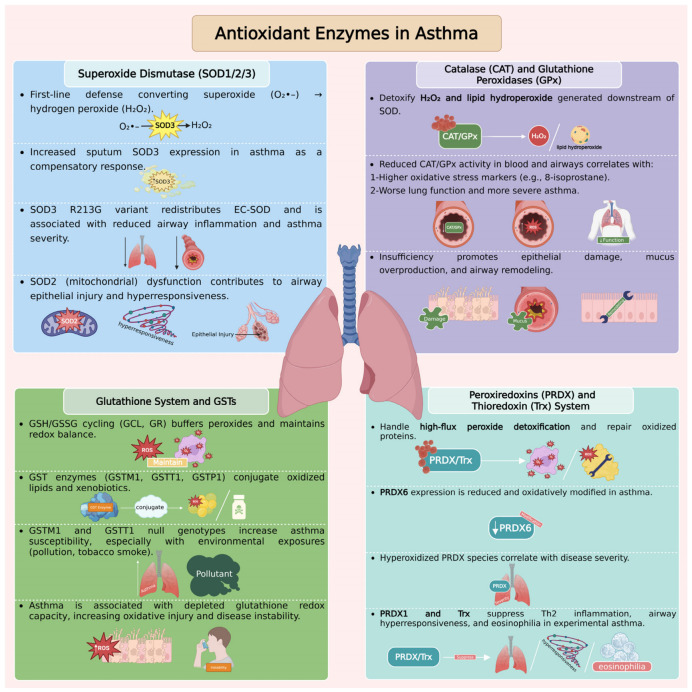
Antioxidant Enzymes in Asthma. The diagram illustrates the roles of antioxidant enzymes in the pathophysiology of asthma. Superoxide dismutase (SOD1/2/3) acts as the first-line defense by converting superoxide (O_2_•) into hydrogen peroxide (H_2_O_2_), with increased SOD3 expression in asthma as a compensatory response. The SOD3 R213G variant is linked with increased airway inflammation and asthma severity. Mitochondrial dysfunction of SOD2 contributes to epithelial injury and airway hyper responsiveness. Catalase (CAT) and glutathione peroxidase (GPx) detoxify H_2_O_2_ and lipid hydroperoxides generated downstream of SOD. Reduced activity of CAT/GPx correlates with higher OS markers and worse lung function. Glutathione (GSH) cycling via glutathione reductase (GR) buffers reactive oxygen species (ROS) and maintains redox balance. GST enzymes, including GSTM1, GSTT1, and GSTP1, conjugate oxidized lipids and xenobiotics. Asthma is associated with depleted glutathione redox capacity, exacerbating oxidative injury. Peroxiredoxins (PRDXs) and thioredoxin (Trx) systems handle peroxide detoxification and repair oxidized proteins. PRDX6 expression is reduced in asthma, correlating with disease severity. The PRDX1/Trx system suppresses T-helper 2 (Th2) inflammation and airway hyper responsiveness. SOD: superoxide dismutase, SOD1/2/3: isoforms of superoxide dismutase, H_2_O_2_: hydrogen peroxide, CAT: catalase, GPx: glutathione peroxidase, GSH: glutathione, GR: glutathione reductase, GST: glutathione S-transferase, GSTM1: glutathione S-transferase mu 1, GSTT1: glutathione S-transferase theta 1, GSTP1: glutathione S-transferase pi 1, ROS: reactive oxygen species, PRDX: peroxiredoxin, Trx: thioredoxin, Th2: T-helper 2. The image was created using the BioRender program, available at https://www.biorender.com.

The OS generated in the inflamed airways extends beyond the lungs and may have systemic consequences. ROS and inflammatory mediators produced by activated pulmonary inflammatory cells may enter the bloodstream, thereby increasing the systemic oxidative load. These oxidants are neutralized by antioxidant enzymes such as GPx and CAT, which are perpetually used to detoxify hydrogen peroxide and other reactive species. In patients with inadequately controlled asthma, persistent oxidant production may exceed the antioxidant defense capabilities, leading to heightened consumption, functional inactivation, and eventual depletion of GPx and CAT activity. Consequently, reduced systemic levels of these enzymes have been identified as biomarkers indicative of heightened OS and compromised antioxidant defense in severe asthma. Some studies have found that serum SOD activity rises during the first stages of OS, but this rise only serves as an insufficient compensation, which shows that serum enzymatic changes function as dynamic disease severity indicators [63]. The evaluation of serum enzymes enables researchers to use a non-invasive approach for assessing the oxidative status of asthma patients that extends beyond traditional lung function assessments (Table 1, Figure 5).

#### 4.1.1. Superoxide Dismutase

SOD is a crucial antioxidative metalloenzyme that serves as the primary defense mechanism against ROS in biological systems. It facilitates the dismutation of the superoxide anion (O_2_•^−^), a highly reactive free radical produced during standard cellular metabolism and inflammatory activities, into molecular oxygen (O_2_) and H_2_O_2_, which is then detoxified by CAT and glutathione peroxidase [69]. Through this method, SOD mitigates oxidative damage to essential macromolecules, including lipids, proteins, and DNA, thereby safeguarding cellular integrity and function [70]. In the respiratory system, specifically within lung tissues, SOD serves a vital protective function by preserving redox equilibrium and mitigating OS-induced inflammation, which is significantly pertinent to asthma pathogenesis [71]. SOD activity is meticulously regulated in response to alterations in the cellular redox state, rising under heightened OS to mitigate ROS buildup [72]. Consequently, variations in SOD enzyme activity operate as a significant indicator of OS and may influence disease severity and development in asthma. Asthmatic patients show decreased SOD activity, which affects both their airway epithelial cells and their serum level. The loss of SOD function results in unprotected lungs, which become vulnerable to superoxide-induced lung damage. Superoxide-induced lung damage includes both lipid peroxidation and surfactant function impairment [64].

Research findings demonstrate a strong positive relationship between serum SOD levels and lung function measurements that include FEV_1_. The research shows that decreased SOD activity causes increased airway blockage, which indicates that complete systemic loss of this essential defense enzyme functions as a diagnostic tool for detecting airflow limitations. The research found that Tunisian asthmatic subjects showed a strong positive relationship between their blood SOD levels and their lung function results, which had a correlation coefficient of r = 0.447 and a statistical significance level of *p* = 0.010 [65]. The models show that SOD levels increase at first because of OS, but SOD levels eventually decline because proteins undergo nitration, which leads to severe airway obstruction [66].

#### 4.1.2. Catalase and Glutathione Peroxidase

CAT and GPx are vital elements of the cellular antioxidant defense system, collaborating to neutralize reactive oxygen species and preserve redox equilibrium. CAT is a heme-containing enzyme that facilitates the fast breakdown of H_2_O_2_, a potentially detrimental by-product of metabolic and inflammatory processes, into water and molecular oxygen, thus averting oxidative damage to cellular macromolecules [73]. Concurrently, glutathione peroxidase, a family of selenium (Se)-dependent enzymes, catalyzes the reduction of H_2_O_2_ and lipid hydroperoxides to water and their respective alcohols, utilizing reduced glutathione (GSH) as a substrate, therefore safeguarding membrane lipids and cellular structures from peroxidative damage [74]. In conjunction with SOD, CAT and GPx constitute the primary enzymatic antioxidant defense, functioning sequentially to neutralize reactive oxygen species produced during OS [36]. In asthma, these enzymes are crucial for alleviating airway oxidative damage and inflammation; diminished activity of CAT and GPx has been linked to increased OS and disease severity [68]. Consequently, the serum activity levels of CAT and glutathione peroxidase behave as significant indicators indicative of antioxidant capability and redox imbalance in asthmatic individuals. Research findings show that GPx levels drop in asthmatic individuals, which results in increased airway hyper responsiveness and more severe clinical symptoms [67]. Clinical studies report that lower blood/airway activities of CAT and GPx track with higher OS markers (e.g., EBC 8-isoprostane) and worse clinical characteristics, supporting an overwhelmed enzymatic defense in moderate–severe disease [75]. A contemporary pediatric/phenotype-oriented synthesis likewise places SOD and CAT/GPx at the “front line” of antioxidant protection in the asthmatic airway. Together, these data position CAT/GPx insufficiency as a contributor to epithelial damage, mucus hypersecretion, and remodeling under high oxidant burden [76,77].

The body reaches its antioxidant capacity limit during “attacks”, which are known as acute exacerbations. Research indicates that GPx and CAT activities can plummet to their lowest levels during these episodes, which show total exhaustion of the protective grid. Clinical evaluations of patients admitted to the emergency room with acute asthma attacks show significantly lower total antioxidant capacity (TAC) and GSH compared to stable outpatients [64]. The specific enzymatic markers experience a “significant depletion”, which serves as an authentic indicator of both uncontrolled disease and oxidative failure during a crisis [63].

#### 4.1.3. Peroxiredoxins and the Thioredoxin System

Peroxiredoxins (PRDXs) and the Trx system constitute a meticulously coordinated antioxidant network essential for cellular redox regulation. PRDXs are thiol-dependent peroxidases that swiftly reduce H_2_O_2_, organic hydroperoxides, and ONOO^−^, thereby mitigating oxidative damage to lipids, proteins, and nucleic acids while simultaneously functioning as sensors and regulators of localized peroxide signaling [78]. The Trx system, consisting of Trx, Trx reductase, and NADPH, restores oxidized PRDXs and sustains protein thiol groups in their reduced form, thus maintaining intracellular redox homeostasis, regulating disulfide exchange, and facilitating cell survival during OS [79,80]. In addition to peroxide detoxification, the PRDX/Trx axis regulates redox-sensitive signaling pathways, transcription factor activity, inflammation, apoptosis, and immune responses, rendering it significantly pertinent to airway diseases where OS exacerbates tissue damage and disease advancement [81]. In asthma, the dysfunction or oxidative alteration of PRDXs, especially PRDX6, may diminish antioxidant defenses and promote chronic airway inflammation, indicating that serum activity or the expression of PRDX/Trx-related components could serve as prognostic biomarkers for OS and asthma severity [82,83].

High-flux peroxide handling and protein repair are facilitated by PRDX1/6 and Trx/Trx reductase. In patients, PRDX6 expression is reduced and undergoes post-translational modifications under OS, while hyper-oxidized PRDX species have been proposed as markers of disease state and severity. Experimental studies show that PRDX1 limits Th2-dominant airway inflammation, and that Trx administration suppresses airway hyper responsiveness and eosinophilic inflammation in allergic asthma models; these findings highlight these enzymes as both biomarkers and potential therapeutic targets [82].

### 4.2. Serum Activity of Pro-Oxidant Enzymes

NOX and dual oxidases (DUOXs) are primary epithelial sources of ROS that drive mucus metaplasia and remodeling. Airway epithelial DUOX1/DUOX2 generate H_2_O_2_ in response to type-2 cytokines and allergens, activating epidermal growth factor receptor (EGFR) signaling and inducing MUC5AC; human airway and model data show DUOX1-dependent cascades (protein kinase C (PKC)–ROS–tumor necrosis factor-alpha converting enzyme (TACE)–EGFR) that causally increase mucin expression and epithelial injury [84]. In neutrophilic and severe asthma, structural cells (epithelium, airway smooth muscle) exhibit increased NOX4 expression/activity, linking constitutive ROS production to ciliary dysfunction and profibrotic transforming growth factor-beta (TGF-β)/Smad signaling; NOX inhibition attenuates these features in experimental systems. Collectively, NOX/DUOX activity provides an upstream mechanistic source for oxidant products detected clinically (e.g., EBC H_2_O_2_, 8-isoprostanes) [85].

**Figure 6:**
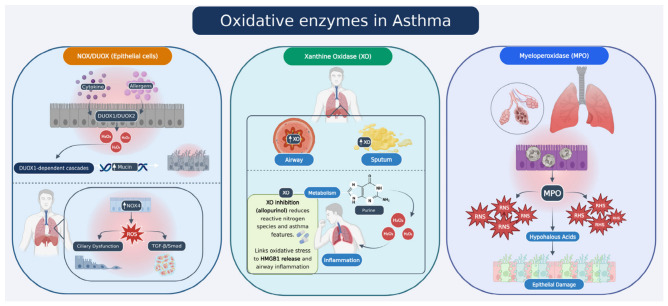
Oxidative Enzymes in Asthma. This diagram illustrates the roles of oxidative enzymes in asthma pathogenesis. NADPH oxidase (NOX) and dual oxidase (DUOX) in epithelial cells are activated by allergens, leading to NOX1-dependent signaling pathways that contribute to airway inflammation, mucus production, and ciliary dysfunction. Xanthine oxidase (XO) is involved in purine metabolism and produces reactive oxygen species (ROS) such as hydrogen peroxide (H_2_O_2_), exacerbating inflammation and sputum production in the airways. Myeloperoxidase (MPO) within neutrophils generates reactive nitrogen species (RNS) and ROS that further damage epithelial cells and contribute to airway remodeling. NOX: NADPH oxidase, DUOX: dual oxidase, XO: xanthine oxidase, MPO: myeloperoxidase, ROS: reactive oxygen species, RNS: reactive nitrogen species, H_2_O_2_: hydrogen peroxide. The image was created using the BioRender program, available at https://www.biorender.com.

Xanthine oxidase (XO) amplifies airway oxidant load and is pharmacologically targetable. XO activity is elevated in the sputum and airways of people with asthma and contributes superoxide/H_2_O_2_ during purine metabolism, especially in allergen-driven inflammation. Translational work demonstrates that allopurinol (XO inhibition) reduces airway reactive nitrogen species in chronic asthma, and recent experimental studies confirm that XO blockade suppresses high mobility group box 1 (HMGB1) secretion and ameliorates asthma features—supporting XO as both a biomarker-linked source of oxidants and a potential therapeutic target in selected phenotypes [86] (Figure 6).

MPO and neutrophil-dominant pathways fuel nitrative/halogenative stress in difficult asthma. Neutrophils from allergic/asthmatic patients display an increased propensity to release MPO, generating hypohalous acids (e.g., HOCl) that damage epithelium and extracellular matrix and correlate with poor disease control. Neutrophilic asthma-frequently steroid-insensitive-features enhanced neutrophil activity and oxidative enzyme output; MPO and related neutrophil proteases thereby link cellular phenotype to oxidative tissue injury and remodeling. These data position MPO as a mechanistically relevant oxidative enzyme in non-eosinophilic endotypes and a plausible contributor to exacerbation-prone disease [87] (Figure 6).

## 5. Genetic Polymorphisms of Oxidative and Antioxidant Enzymes as Biomarkers of Asthma Risk and/or Severity

**Figure 7:**
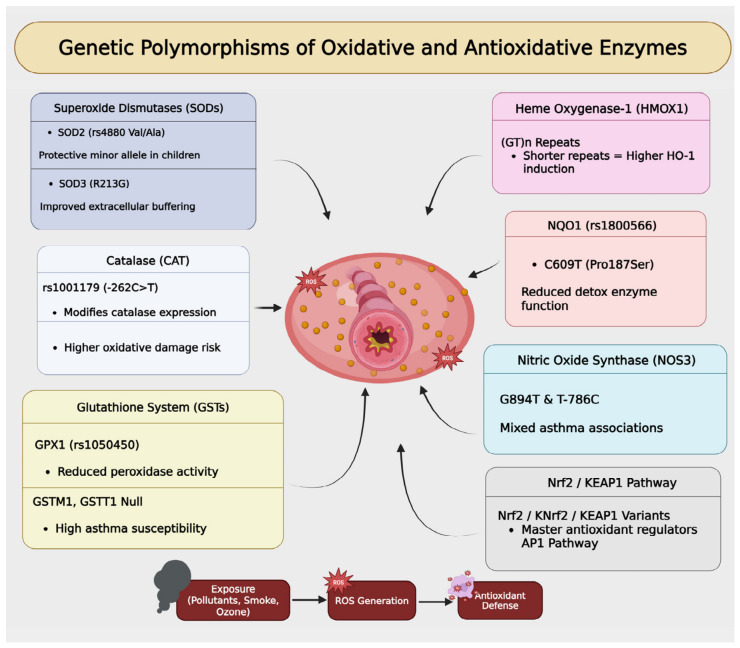
Genetic Polymorphisms of Oxidative and Antioxidative Enzymes. This figure highlights various genetic polymorphisms that influence oxidative and antioxidative enzyme functions, impacting asthma susceptibility and progression. Superoxide dismutase (SOD) polymorphisms, including SOD2 (rs4880 Val/Ala) and SOD3 (R213G), are associated with protective effects or improved extracellular buffering, influencing OS levels. Catalase (CAT) polymorphisms (rs1001179 −262C>T) modify catalase expression, with higher oxidative damage risk in individuals with the T allele. Heme oxygenase-1 (HO-1) polymorphisms, particularly (GT)n repeats, affect HO-1 induction, with shorter repeats leading to higher expression. Nitric oxide synthase (NOS3) polymorphisms (G894T & T-786C) have mixed associations with asthma. Reduced detoxification capacity is seen with NQO1 (rs1800566) and GST gene variations (GPX1 rs1050450 and GSTM1/GSTT1 null), which are linked to higher asthma susceptibility. The Nrf2/KEAP1 pathway variants regulate antioxidant defense by controlling ROS generation, with genetic variants influencing antioxidant response pathways. SOD: superoxide dismutase, CAT: catalase, HO-1: heme oxygenase-1, NQO1: NAD(P)H quinone dehydrogenase 1, NOS3: nitric oxide synthase, GPX1: glutathione peroxidase 1, GST: glutathione S-transferase, Nrf2: nuclear factor erythroid 2-related factor 2, KEAP1: Kelch-like ECH-associated protein 1, ROS: reactive oxygen species. The image was created using the BioRender program, available at https://www.biorender.com.

Asthma susceptibility (genetic variants that increase the probability of developing asthma in the first place) and asthma severity (variants that among individuals already diagnosed with asthma, predict more severe disease, worse lung function, higher exacerbation frequency, or greater treatment requirements) are not equivalent constructs. The majority of published genetic association studies in the redox/antioxidant enzyme field were originally designed to test susceptibility, not severity. As a consequence, many variants discussed below have robust case–control susceptibility evidence but limited or indirect severity evidence (Table 2 and Table 3). Where severity evidence exists, defined here as a significant association with FEV\u2081 decline, exacerbation frequency, hospitalization, or treatment step among asthmatics, it is explicitly in Table 4 under the column “Primary Association (Risk/Severity/Both)” and summarized in the Key Evidence paragraph at the end of each subsection. Variants for which severity evidence is absent or only mechanistically inferred are labeled as “Risk (susceptibility)” to ensure that the reader can accurately appraise the clinical relevance of each finding. This framework follows the approach recommended by Slager et al. and Raby, who called for future genetic studies to be designed specifically to separate susceptibility from severity loci [88,89]. Genetic variation in oxidant/antioxidant defense pathways contributes to inter-individual differences in redox capacity and can modify asthma susceptibility and severity, particularly under environmental oxidant exposures. Key functional variants linked to asthma outcomes are summarized in Figure 7.

### 5.1. Genetic Polymorphisms of Antioxidant Enzymes

They act as catalysts and are efficiently recycled after working. The enzymes that make up this enzymatic antioxidant system are GPx, GR, GST, SOD, CAT, paraoxonase 1 (PON1), heme oxygenase-1 (HO-1), NAD(P)H:quinone oxidoreductase 1 (NQO1), and Trx/Trx reductase, and PRDXs.

PON1 is an enzyme linked with high-density lipoprotein (HDL) that possesses antioxidant, anti-inflammatory, and xenobiotic-detoxifying capabilities [128]. In addition to hydrolyzing organophosphate compounds and other detrimental substrates, PON1 safeguards lipoproteins and cellular membranes from oxidative damage by breaking down lipid peroxides. PON1 complements the traditional antioxidant defense system, which includes *SOD*, *CAT*, and *GPx* [129]. A reduction in PON1 activity may correlate with an accumulation of lipid peroxides and systemic OS, potentially exacerbating the strain on other antioxidant enzymes and leading to their functional depletion [130]. PON1 activity is diminished in asthma, correlating with reduced antioxidant defense and heightened OS [131]. Current data suggest that diminished PON1 activity may correlate with the prevalence and severity of asthma; however, this link remains inadequately described and may be influenced by factors such as age, phenotype, environmental exposure, HDL function, and *PON1* genetic variations. PON1 should be regarded as an ancillary biomarker of OS dysregulation in asthma, and subsequent study ought to evaluate its correlation with asthma severity, exacerbation frequency, pulmonary function, FeNO levels, and responsiveness to corticosteroid treatment [132].

SOD family (SOD2 Val16Ala; SOD3 Arg213Gly) and mitochondrial-extracellular redox control. Among the antioxidant enzymes, SOD2 and SOD3 (EC-SOD) polymorphisms recur in asthma genetics. In a pediatric case–control cohort, the SOD2 rs4880 (Val16Ala) variant was significantly associated with reduced susceptibility to childhood bronchial asthma. The observed protective association was evident under allelic, recessive, and dominant genetic models, and was also present in both atopic and non-atopic subgroups. Collectively, these findings indicate that the SOD2 rs4880 polymorphism may confer protection against the development of childhood bronchial asthma in this population [133] (Figure 7).

CAT detoxifies H_2_O_2_ and is therefore a key enzymatic antioxidant downstream of superoxide dismutation. In a pediatric study published in *Archivos de Bronconeumología*, the CAT promoter polymorphism rs1001179 (−262C>T) was evaluated in asthmatic children together with markers of oxidative damage, and the TT genotype was found more frequently in asthmatic patients and was associated with greater oxidative injury. These findings support the view that promoter variation in CAT may influence antioxidant defense and contribute to the clinical expression of childhood asthma. Prior functional studies cited by the authors further support the biological plausibility that rs1001179 affects CAT regulation in vivo [92].

Meta-analytic evidence supports an association between GSTM1 null and GSTT1 null genotypes and asthma susceptibility, reinforcing the importance of glutathione-dependent antioxidant defense in asthma pathogenesis. Because GST enzymes participate in the detoxification of reactive oxygen species, reduced GST activity may contribute to OS and thereby increase the disease risk. The heterogeneity across studies further suggests that gene–environment interactions are likely to influence the magnitude of these associations [5].

*HO-1* promoter (GT)n and oxidant defense under environmental stress. HO-1 induction confers cytoprotection via biliverdin/bilirubin and CO signaling. The length of the (GT)n promoter repeat modulates inducibility: shorter alleles track with stronger HO-1 upregulation [134]. In a longitudinal study of California children, shorter HO-1 GTn repeats were associated with a reduced risk of new-onset asthma among non-Hispanic Whites, with the protective effect being most evident in low-ozone communities. These findings support a gene–environment interaction in OS-related asthma, and together with prior respiratory and population studies cited by the authors, suggest that HO-1 promoter variability may influence susceptibility to oxidant-related lung phenotypes [93].

NQO1 is an antioxidant enzyme involved in cellular defense against OS. The rs1800566 polymorphism (C609T; Pro187Ser) has been linked to increased susceptibility to NOx-induced lung injury, and computational analyses indicate that this substitution destabilizes the NQO1 protein and alters structural features relevant to its function. These findings support the biological plausibility that NQO1 variation may modify susceptibility to pollutant-related respiratory injury, although direct effects on asthma risk or severity were not established by this study [135].

Nrf2 (NFE2L2)/KEAP1 variants and redox program inducibility. Because Nrf2–KEAP1 controls the transcription of many antioxidant genes (*SODs*, *GPX*, *HO-1*, and *NQO1*), variants in this pathway could shape global redox capacity. Case–control work in children exposed to traffic pollution linked NFE2L2 polymorphisms to infection-triggered asthma exacerbations, whereas other cohorts found no main-effect association with asthma status, suggesting context-specific or interaction-driven effects. Beyond asthma, functional human studies and reviews confirm that Nrf2 promoter variation can alter inducibility, providing a mechanistic substrate for differences in antioxidant response, and potentially, steroid sensitivity in oxidant-stressed endotypes [136].

### 5.2. Genetic Polymorphisms of Oxidative Enzymes

#### 5.2.1. Nitric Oxide Synthase

Nitric oxide synthase (NOS) is a crucial redox-regulating enzyme that catalyzes the transformation of L-arginine into NO and L-citrulline, consequently affecting airway tone, vascular regulation, host defense, and inflammatory signaling in the respiratory system. The constitutive isoforms NOS1 and NOS3 produce low physiological levels of NO in the airways, facilitating bronchodilation and epithelial homeostasis, while the inducible NOS2 is upregulated during inflammation, significantly contributing to the increased exhaled nitric oxide seen in asthma. Due to the involvement of NO in eosinophilic inflammation, airway hyper responsiveness, and remodeling, genetic variations in *NOS* genes can influence both susceptibility to asthma and the manifestation of the disease [108].

Population-based cohort analyses (epidemiological study on the genetic and environment of asthma (EGEA) research; *n* ≈ 1277) similarly demonstrated that several SNPs within *NOS* genes correlate with FeNO levels, nitrite/nitrate concentrations, and blood eosinophils, underscoring their influence on clinically detectable inflammatory biomarkers [137]. Case–control studies corroborate these findings, indicating that NOS2 polymorphisms (e.g., rs10459953) substantially elevate the risk of pediatric allergic asthma, implying a genetic influence on disease susceptibility [106]. *NOS1* polymorphisms have been associated with asthma diagnosis and immunoglobulin E (IgE)-mediated responses across many groups, underscoring the significance of neuronal NO signaling in airway hyper responsiveness [138]. Meta-analyses encompassing over 4000 asthma cases have indicated that particular NOS2 repeat variations affect the exhaled NO levels and treatment results, hence reinforcing their clinical significance as biomarkers [110]. These collectively illustrate that *NOS* genetic variability influences NO production and contributes to the heterogeneity of asthma phenotypes, disease progression, and therapeutic response, positioning NOS polymorphisms as promising candidates for prognostic panels in redox-based asthma evaluation.

#### 5.2.2. Myeloperoxidase

MPO is a heme-containing peroxidase mostly found in neutrophils, where it facilitates the interaction between H_2_O_2_ and chloride ions to produce HOCl, a powerful oxidant crucial for microbial eradication and innate immune defense. Excessive MPO-derived oxidants, however, lead to OS, tissue damage, and persistent airway inflammation, establishing MPO as a pivotal mediator in asthma pathophysiology [139]. Genetic variants in the *MPO* gene, especially the notable promoter variant −463G>A (rs2333227), have been demonstrated to affect transcriptional activity, with the A allele linked to diminished MPO expression and modified oxidative capability. Clinical studies have shown that this polymorphism correlates with asthma susceptibility and severity, as well as circulating MPO levels, suggesting a functional genotype–phenotype link in airway inflammation (ERS clinical trial; *n* ≈ 79) [105]. Moreover, case–control clinical studies have repeatedly demonstrated significantly increased blood MPO levels in asthmatic patients relative to healthy controls, reinforcing its function as a biomarker for illness prevalence and inflammatory burden [140]. Additional mechanistic clinical evidence indicates that neutrophils from asthmatic patients secrete elevated levels of MPO, which correlates with airway impairment and diminished pulmonary function, hence associating MPO activity with illness severity [87]. Furthermore, recent investigations in both pediatric and adult populations have established that elevated MPO levels in sputum and blood correlate with neutrophilic inflammation and moderate-to-severe asthma phenotypes, underscoring its significance in disease progression and phenotypic diversity [141]. The data demonstrate that *MPO* genetic variants and enzyme activity strongly influence oxidative imbalance, inflammatory enhancement, and clinical variability in asthma, hence endorsing their incorporation as predictive biomarkers in redox-based assessments.

#### 5.2.3. NADPH Oxidases

NOX are specialized transmembrane enzyme complexes that serve as a principal source of ROS in the respiratory system by catalyzing the exchange of electrons from NADPH to molecular oxygen, resulting in the production of superoxide anion (O_2_•^−^). In contrast to other oxidative enzymes, NOX enzymes (specifically NOX1, NOX2, NOX4, and DUOX1/2) are specialized systems for reactive oxygen species production, crucial for host defense, epithelial signaling, mucosal immunology, and airway remodeling. Dysregulated NOX activity, however, leads to increased OS, epithelium damage, and persistent inflammation in asthma. Genetic polymorphisms in NOX-related genes, such as *CYBA* (p22phox), *CYBB (NOX2*), and *DUOX1/2*, have been demonstrated to affect reactive oxygen species formation and predisposition to inflammatory airway disorders. Clinical investigations indicate that the *CYBA* polymorphism (e.g., C242T, rs4673) modifies NADPH oxidase activity and correlates with heightened OS and asthma susceptibility in population-based cohorts [142]. Moreover, variations in DUOX1 and DUOX2 enzymes prominently expressed in airway epithelium have been associated with compromised epithelial host defense and heightened airway inflammation in asthma patients, underscoring their significance in disease pathophysiology [143]. Extensive cohort analyses and genetic association studies have demonstrated that *NOX* gene variations are associated with indicators of OS and deterioration in lung function, hence affirming their functional and clinical significance [144]. Furthermore, translational clinical investigations suggest that modified NOX2 activity in inflammatory cells leads to increased ROS generation, neutrophilic inflammation, and airway hyper responsiveness, especially in severe asthma phenotypes [68]. The data indicate that *NOX* genetic polymorphisms regulate ROS production, consequently affecting oxidative imbalance, airway inflammation, and clinical variability in asthma, thus endorsing their incorporation into redox-based prognostic panels.

#### 5.2.4. Xanthine Oxidase

Xanthine oxidoreductase (XOR), encoded by the *xanthine dehydrogenase (XDH)* gene, is a crucial molybdenum-dependent enzyme that facilitates purine metabolism by catalyzing the oxidation of hypoxanthine to xanthine and subsequently xanthine to uric acid. XOR is available in two interconvertible forms: XDH and XO. The latter preferentially produces ROS, such as superoxide anion and H_2_O_2_, thereby associating purine metabolism with OS and inflammation [145]. The capacity to generate ROS leads to epithelial damage, endothelial impairment, and the enhancement of inflammatory signaling pathways in the airways [146]. Clinical evidence indicates that patients with refractory asthma show markedly heightened sputum XO activity and augmented nitrative stress relative to those with moderate asthma and healthy controls, suggesting a direct association of XO with illness severity [147]. Additionally, airway epithelial cells from asthmatic patients exhibit elevated uric acid synthesis due to enhanced XDH/XO activity, which can be mitigated by allopurinol, underscoring the significance of this pathway in human airway inflammation [148]. Genetic polymorphisms in the *XDH* gene, encompassing coding and promoter variants, dramatically modify enzyme activity and transcriptional control, thereby affecting systemic OS levels [149,150]. While asthma-specific genetic investigations are scarce, recent clinical genetic research has indicated that *XDH* polymorphisms correlate with an elevated risk of inflammatory disorders, including sepsis and acute respiratory distress syndrome (ARDS), alongside increased circulating XOR activity, thereby underscoring their influence on OS in human pathology [115]. The data indicate that genetic diversity in XO/XOR contributes to oxidative imbalance, airway inflammation, and disease severity, hence supporting its possible inclusion in redox-based predictive panels for asthma.

## 6. Role of Micronutrients and Cofactors

Se, zinc (Zn), copper (Cu), and manganese (Mn) are obligate cofactors for core antioxidant enzymes that are routinely measured as asthma biomarkers—GPx (Se), Cu/Zn-SOD (Zn, Cu), and Mn-SOD (Mn). Clinical studies consistently report that circulating micronutrient status covaries with enzymatic antioxidant activity in blood/EBC and with oxidative-damage indices (e.g., 8-isoprostane, MDA), supporting their interpretation as biochemical context markers for redox assays. In classic intrinsic-asthma work, lower serum Se tracked with reduced GPx activity, aligning micronutrient deficiency with impaired peroxide detoxification; more recent pediatric case–control data likewise demonstrate coupled changes in Se and serum GPx activity [151]. Selected clinical evidence linking micronutrient cofactors (e.g., Se, Zn, Cu, Mg, vitamin D) to asthma control and redox-related outcomes is summarized in Table 5.

Beyond case–control differences, a 2024 study found a dose–response correlation between Se intake and lung function in asthma, proposing an intake window (~138–200 µg/day) that maximized pulmonary outcomes while avoiding excess—biologically coherent with Se’s role in GPx catalysis and glutathione redox cycling. Together with older enzymology showing serum Se and serum GPx coupling, these findings support using Se (dietary or serum) alongside GPx activity as a composite biomarker of systemic antioxidant capacity in asthma cohorts [156].

Zn supports epithelial barrier integrity and Cu/Zn-SOD structure/function. A 2025 narrative review synthesizing interventional and observational evidence concluded that lower Zn is common in pediatric asthma and that adjunct Zn can improve symptoms and spirometry in some trials, although study quality and heterogeneity limit firm guidance. Newer case–control data in children also show significantly lower serum Zn in asthmatics vs. controls, reinforcing its potential use as a state biomarker (when interpreted with inflammation and diet). Small clinical reports suggest that 20 mg/day Zn improved lung function and reduced severe exacerbations, warranting larger phenotype-stratified randomized controlled trials (RCTs) [157].

Given its immunomodulatory actions, vitamin D has been widely examined as a biomarker/target in asthma. The 2023 Cochrane update found no overall reduction in exacerbations or improved control across mixed populations, emphasizing heterogeneity by baseline deficiency, age, and regimen. Interpreting vitamin D as a risk-modifier biomarker, rather than a blanket treatment, fits the evidence: low 25(OH)D may identify subgroups with higher exacerbation risk or steroid-response issues, but routine supplementation for all asthmatics is not supported [158].

Antioxidant vitamins show endpoint-specific benefits. For exercise-induced bronchoconstriction (EIB), a meta-analysis found vitamin C reduced the post-exercise FEV_1_ decline (≈8.4 percentage-points absolute; ≈48% relative), consistent with acute ROS scavenging during exertion. In persistent asthma, however, trials are heterogeneous and largely inconclusive, suggesting that these vitamins function best as situational biomarkers/adjuncts rather than chronic controllers [159].

Mg participates in redox-enzyme stabilization and smooth-muscle relaxation. While serum Mg is not a reliable chronic biomarker of control, intravenous or nebulized MgSO_4_ is one of the few micronutrient-based interventions with repeat meta-analytic support in acute severe, refractory exacerbations, improving lung function and reducing admissions in specific settings. Clinically, Mg highlights a use-case where a micronutrient informs treatment decisions in emergency asthma, even if its baseline level is a poor chronic predictor [160].

Because Cu and Zn act antagonistically in redox chemistry, the serum Cu:Zn ratio is being explored as a low-cost proxy of inflammatory/oxidative load (higher ratios track with inflammatory markers). Although data are stronger in infections and systemic diseases than in asthma per se, this ratio’s biology overlaps with airway redox pathways, making it a candidate adjunct biomarker to contextualize Zn- or SOD-related findings in multi-marker panels. Prospective asthma-specific validation is still needed [161]. Dietary pattern context for micronutrient biomarkers. Micronutrient biomarkers reflect not only supplementation but also dietary patterns. Systematic reviews indicate that Mediterranean-style diets, richer in antioxidant nutrients and polyphenols, are protectively associated with child asthma/wheeze in several cohorts, supporting a diet-anchored interpretation of serum micronutrients (Se, Zn, vitamins) and of oxidant outputs (EBC H_2_O_2_, 8-isoprostanes). Using diet indices alongside micronutrient levels and enzyme activities improves ecological validity of redox biomarker assessment [162].

## 7. Biomarkers of Oxidative Damage in Asthma

OS damage biomarkers in asthma can be categorized into indicators of lipid, DNA, and protein oxidation. In asthma, reactive oxygen and nitrogen species arise from airway epithelium, activated inflammatory cells (eosinophils/neutrophils/macrophages), mitochondrial dysfunction, and oxidant-producing enzymes (e.g., NADPH oxidases, nitric oxide synthases), and are amplified by triggers such as allergens, viral infections, cigarette smoke, ozone, and particulate air pollution [163]. OS injures membranes, nucleic acids, and proteins, producing measurable end-products that can support phenotyping (eosinophilic vs. neutrophilic), severity stratification, and the monitoring of control/exacerbations [164]. Key oxidative damage biomarkers reported in asthma, including lipid, DNA, and protein oxidation readouts, their measurement approaches, and clinical associations are summarized in Table 6.

### 7.1. Lipid Peroxidation Products

MDA (MDA; commonly measured by thiobarbituric acid reactive substances (TBARS)), 4-hydroxynonenal (4-HNE), 8-iso-prostaglandin F_2_α (8-iso-PGF_2_α), lipid hydroperoxides (LOOHs), and oxidized low-density lipoprotein (oxLDL) are key lipid peroxidation products generated when ROS attack polyunsaturated fatty acids within cellular and lipoprotein membranes [172]. Among these, MDA and 4-HNE are highly reactive aldehydes that form stable adducts with proteins and nucleic acids, thereby amplifying inflammatory signaling and structural airway damage, while isoprostanes such as 8-iso-PGF_2_α are prostaglandin-like end products regarded as among the most reliable in vivo biomarkers of oxidative lipid injury [173,174]. In asthma, increased lipid peroxidation has been consistently demonstrated in both airway-derived and systemic compartments. Elevated levels of MDA and related lipid oxidation products have been detected in exhaled breath condensate, induced sputum, plasma, and serum of asthmatic patients compared with healthy controls, reflecting enhanced oxidative burden in the inflamed airway. Importantly, higher concentrations of lipid peroxidation markers have been associated with poor asthma control, increased airway inflammation, and disease severity, particularly in uncontrolled or severe asthma phenotypes [175]. OxLDL and altered oxLDL-related redox profiles further indicate that oxidative lipid modification extends beyond the airways, contributing to systemic inflammation and endothelial dysfunction in asthma.

Environmental exposures relevant to asthma pathogenesis including allergens, viral infections, cigarette smoke, ozone, and particulate air pollution can further intensify lipid peroxidation processes, leading to dynamic changes in these biomarkers during exacerbations [176]. Although individual markers show variability across studies, composite panels of lipid peroxidation biomarkers are increasingly recognized as valuable tools for characterizing OS burden, monitoring treatment responses (e.g., ICS), and identifying patients with persistent oxidative injury in asthma [177].

#### 7.1.1. Malondialdehyde as Lipid Peroxidation Biomarkers

MDA is a reactive aldehyde generated during the oxidative degradation of polyunsaturated fatty acids, particularly arachidonic acid, following ROS-mediated membrane damage [178]. Elevated MDA levels reflect enhanced lipid peroxidation and tissue injury, and MDA can form adducts with lysine residues on proteins, leading to structural and functional alterations that promote airway inflammation [178]. In asthma, increased MDA concentrations have been reported in exhaled breath condensate and blood samples, particularly in patients with uncontrolled or severe disease, supporting its utility as a marker of OS burden and airway injury [178]. MDA levels showed significant elevation in emergency room patients who experienced acute situations compared to outpatients with stable conditions. This finding demonstrates that systemic damage indicators can accurately measure the severity of pulmonary inflammation present during medical emergencies [64]. The inverse correlation shows that researchers studying both adult and pediatric groups found plasmatic MDA levels to have a strong negative relationship with TAC (r = −0.74, *p* < 0.001) and reduced glutathione level (r = −0.69, *p* < 0.001) [64].

#### 7.1.2. 4-Hydroxynonenal

4-HNE is a highly reactive α, β-unsaturated aldehyde generated during OS-driven lipid peroxidation of polyunsaturated fatty acids in airway cell membranes. Due to its strong electrophilic nature, 4-HNE readily forms stable Michael adducts with nucleophilic amino acid residues in proteins, particularly histidine, cysteine, and lysine, making 4-HNE–protein adducts a sensitive and durable marker of lipid peroxidation-associated oxidative damage [179,180].

In asthma, increased OS within the airways, largely driven by activated eosinophils, neutrophils, macrophages, and epithelial cells, leads to the enhanced generation of ROS, which in turn promotes lipid peroxidation and the formation of secondary aldehydes such as 4-HNE [163]. Elevated levels of lipid peroxidation products, including 4-HNE and MDA, have been detected in biological samples from asthmatic patients, such as exhaled breath condensate, sputum, and bronchoalveolar lavage fluid, reflecting an increased oxidant burden in the asthmatic airway [63,181].

Mechanistically, 4-HNE contributes to asthma pathophysiology by modifying structural and signaling proteins in airway epithelial and immune cells, thereby amplifying inflammatory signaling pathways such as NF-κB and MAPKs and promoting cytokine production, mucus hypersecretion, and airway hyper responsiveness [182]. Persistent OS in asthma may overwhelm local antioxidant defenses, allowing for sustained accumulation of 4-HNE–protein adducts, which can perpetuate chronic airway inflammation and tissue dysfunction [183]. Clinically, increased airway lipid peroxidation markers including 4-HNE have been associated with asthma severity and exacerbations, particularly during periods of heightened inflammation, underscoring their potential utility as biomarkers of disease activity and oxidative injury in asthma [184]. Together, these findings highlight 4-HNE as both a mechanistic mediator and a biomarker of OS-driven airway pathology in asthma.

#### 7.1.3. 8-Isoprostane

8-Iso-PGF_2_α (8-iso-PGF_2_α; also referred to as 15-F_2_t-isoprostane/8-epi-PGF_2_α) is a chemically stable, prostaglandin-like compound generated in vivo during OS through non-enzymatic free radical peroxidation of arachidonic acid in membrane lipids, making it a widely used biomarker of lipid peroxidation. It is formed in situ in lipid membranes and later released (e.g., by phospholipases), and can be quantified in biological fluids including EBC, plasma, bronchoalveolar lavage, and urine [185]. In asthma, evidence from EBC studies suggests a tendency toward higher 8-isoprostane levels versus controls, although the findings are heterogeneous. A systematic review and meta-analysis of adult asthma studies reported that results were inconsistent across studies, but a pooled random-effects analysis (from four eligible studies) identified a statistically significant between-group mean difference (~+21.62 pg/mL) in EBC 8-isoprostane for asthma vs. controls, with substantial heterogeneity (I^2^ ≈ 94%), limiting clear diagnostic thresholds [4].

More recent clinical data also support associations between 8-iso-PGF_2_α and asthma phenotype/severity features. In a cross-sectional study of 128 adults with asthma on ICS/LABA therapy, urinary 8-iso-PGF_2_α was significantly higher in noneosinophilic asthma than eosinophilic asthma (and showed moderate discrimination for noneosinophilic asthma with AUC = 0.678). Urinary 8-iso-PGF_2_α also correlated positively with neutrophilic inflammation markers (MPO r = 0.350; MCP-1 r = 0.315) and airway remodeling markers (MMP-9 r = 0.254; TIMP-1 r = 0.196; TGF-β1 r = 0.321), while showing negative correlations with lung function (e.g., FEV_1_%, FEV_1_/FVC, FEF25–75%) and higher levels in those with poorer symptom control scores [186]. Methodologically, interpretation across studies requires caution: the adult EBC literature highlights important pre-analytical and analytical variability (e.g., storage considerations to prevent in vitro formation, and assay differences where mass spectrometry methods are often more sensitive/selective than immunoassays). 8-Isoprostane functions as a “footprint” for lipid peroxidation, which appears at high concentrations in asthmatic plasma and directly correlates with the severity of their condition [66].

#### 7.1.4. Isofurans, Neuroprostanes, and Neurofurans

Isofurans, neuroprostanes, and neurofurans are advanced lipid peroxidation products generated through non-enzymatic free-radical oxidation of polyunsaturated fatty acids and are considered sensitive markers of OS under specific biological conditions. Isofurans are derived from arachidonic acid and are preferentially formed under conditions of elevated oxygen tension, whereas neuroprostanes and neurofurans originate mainly from docosahexaenoic acid and adrenic acid, lipids that are highly enriched in neural membranes, thereby providing insight into oxidative injury within nervous tissue [187].

Asthma is characterized by chronic airway inflammation and increased OS, with enhanced lipid peroxidation documented primarily through classical markers such as isoprostanes and aldehydic end products [188]. However, available asthma-focused studies have largely concentrated on airway and systemic OS biomarkers measurable in exhaled breath condensate, blood, or urine, rather than on lipid peroxidation products specific to neural or high-oxygen microenvironments [189]. Given that OS in asthma is predominantly studied in the context of airway epithelial injury, immune cell activation, and bronchial hyperresponsiveness, current data do not clarify whether these advanced lipid peroxidation markers are generated at detectable levels in asthma or whether they contribute meaningfully to disease mechanisms beyond the respiratory compartment [190]. As such, while isofurans, neuroprostanes, and neurofurans are well-established indicators of oxidative damage in other biological settings, their relevance to asthma-associated OS remains undefined based on existing evidence from the provided literature.

#### 7.1.5. Lipid Hydroperoxides

LOOHs are the primary molecular products formed at the early stages of lipid peroxidation when reactive oxygen species abstract hydrogen atoms from polyunsaturated fatty acids within cellular membranes, generating lipid peroxyl radicals that rapidly yield LOOHs. Although chemically unstable, LOOHs play a central role in propagating oxidative chain reactions and decompose into secondary reactive aldehydes, including MDA and 4-hydroxynonenal, thereby amplifying oxidative injury in biological tissues [2,191]. In asthma, chronic airway inflammation is accompanied by sustained OS driven by activated eosinophils, neutrophils, and macrophages, which generate high levels of reactive oxygen and halogen species capable of initiating lipid peroxidation. Clinical studies demonstrate elevated circulating lipid hydroperoxide levels in patients with asthma compared with healthy individuals, reflecting increased systemic lipid peroxidation [192]. Notably, higher serum LOOH concentrations have been reported following bronchial provocation with hypertonic saline in asthmatic patients exhibiting airway hyper responsiveness, indicating that intensified lipid peroxidation accompanies exaggerated bronchoconstrictive responses [192]. Mechanistically, LOOH accumulation in asthma is closely linked to MPO-activated leukocytes and iron-dependent oxidative reactions, which enhance membrane damage, epithelial injury, and inflammatory signaling within the airways. Experimental and clinical data further associate increased lipid hydroperoxide formation with impaired antioxidant defenses, including reduced vitamin E and ceruloplasmin levels, contributing to redox imbalance and heightened airway reactivity [188]. Additionally, emerging evidence implicates lipid hydroperoxide accumulation as a hallmark of ferroptosis in airway epithelial cells, a regulated cell death pathway that exacerbates airway inflammation and remodeling in asthma [191].

Overall, available evidence supports elevated lipid hydroperoxide formation as a marker of oxidative membrane damage in asthma and links LOOHs to airway inflammation and hyper responsiveness. However, while associations with disease mechanisms are well-documented, the extent to which LOOH levels directly predict long-term clinical outcomes in asthma remains incompletely characterized based on current data.

#### 7.1.6. Oxidized Low-Density Lipoprotein

oxLDL is generated when LDL particles undergo oxidative modification of their lipid core and apolipoprotein B, producing pro-inflammatory lipoproteins that promote OS and inflammatory signaling, in asthma, systemic OS driven by chronic airway inflammation and activated immune cells contributes to increased lipid oxidation, resulting in elevated circulating oxLDL levels compared with healthy individuals [168,193].

In the ASTHMA-FENOP study, serum oxLDL measured as 4-hydroxynonenal-modified LDL was significantly higher in adults with asthma than in matched controls, even after adjustment for demographic and clinical confounders, indicating enhanced systemic lipid peroxidation in asthma [168]. Mechanistically, oxLDL has been implicated in amplifying oxidative and inflammatory pathways relevant to asthma pathophysiology. Dyslipidemia-associated OS can increase oxLDL formation, which in turn activates redox-sensitive signaling pathways such as MAPKs and NF-κB, promoting reactive oxygen species generation and inflammatory mediator release [194,195]. Experimental and clinical evidence suggests that oxLDL may contribute to airway inflammation and impaired lung function by linking systemic metabolic dysfunction with pulmonary immune responses [194]. Clinically, elevated oxLDL levels in asthma are associated with the broader phenotype of dyslipidemia-related asthma, which has been linked to worse lung function, increased airway inflammation, and poorer asthma control [196]. However, while oxLDL consistently distinguishes asthmatic individuals from non-asthmatic controls as a marker of systemic OS, evidence directly linking oxLDL dynamics to asthma exacerbation risk or long-term clinical outcomes remains limited based on current data. Taken together, oxLDL represents a mechanistic biomarker of oxidative lipid injury in asthma and a potential indicator of disease-related metabolic and inflammatory burden.

### 7.2. DNA Damage Biomarkers

#### 7.2.1. 8-Hydroxy-2′-Deoxyguanosine

8-Hydroxy-2′-deoxyguanosine (8-OHdG) is a well-established biomarker of oxidative DNA damage and reflects the detrimental effects of ROS on genomic integrity, leading to mutations and cellular dysfunction. In asthma, persistent airway inflammation and activation of immune cells such as eosinophils, neutrophils, and macrophages contribute to excessive ROS production, thereby promoting oxidative DNA injury. Consequently, 8-OHdG has been proposed as a relevant indicator of systemic OS in asthmatic patients [168,197]. Evidence from clinical studies indicates that asthmatic individuals exhibit altered levels of OS biomarkers, including 8-OHdG, when compared with healthy controls. In a cross-sectional study evaluating systemic OS in adult asthma, serum 8-OHdG was assessed alongside other oxidative damage markers, such as oxidized LDL and protein carbonyls. Although not all oxidative markers differed significantly between asthmatic patients and controls, 8-OHdG was included among the key biomarkers characterizing systemic OS, supporting the concept that oxidative DNA damage is involved in asthma pathophysiology and may vary according to disease heterogeneity and inflammatory burden [163,168].

Further insight is provided by studies examining asthma under conditions of environmental stress. In a prospective observational study of inhaled corticosteroid-treated, well-controlled adult asthmatic patients exposed to fine particulate matter (PM2.5), urinary 8-OHdG was measured as a marker of oxidative DNA damage during both high- and low-pollution periods. The results showed that urinary 8-OHdG levels in asthmatic patients were not significantly elevated compared with healthy controls, suggesting that effective anti-inflammatory treatment may attenuate PM-induced oxidative DNA damage. These findings highlight that 8-OHdG levels in asthma may be influenced by disease control status, therapeutic interventions, and environmental exposure rather than asthma presence alone [198]. In addition, metabolic comorbidities frequently associated with asthma may further modulate OS. Dyslipidemia, which has emerged as a treatable trait in asthma, is known to promote ROS generation and systemic OS. Oxidative mechanisms linked to abnormal lipid metabolism can amplify inflammatory signaling pathways and DNA damage, potentially contributing to elevated oxidative biomarkers such as 8-OHdG in specific asthma phenotypes. This interaction suggests that oxidative DNA damage in asthma may reflect not only airway inflammation, but also systemic metabolic dysfunction [194]. Overall, these findings indicate that 8-OHdG represents a meaningful biomarker of oxidative DNA damage in asthma, although its levels appear to be modulated by disease severity, environmental exposures, pharmacological treatment, and associated metabolic traits. This variability underscores the complexity of OS responses in asthma and supports further investigation into the clinical utility of 8-OHdG as a marker of systemic oxidative burden in distinct asthma phenotypes.

#### 7.2.2. 8-Oxoguanine

8-Oxoguanine (8-oxoG) is a highly mutagenic oxidized guanine base formed when ROS attack DNA. Its deoxynucleoside counterpart, 8-OHdG, is generated during base excision repair (BER) following the excision of 8-oxoG by repair enzymes such as 8-oxoguanine DNA glycosylase-1 (OGG1) and is subsequently released into the circulation and excreted in urine [199]. While 8-oxoG reflects intracellular genomic DNA damage, 8-OHdG serves as a stable systemic marker of oxidative DNA injury. Both biomarkers are widely used to assess OS-mediated DNA damage in chronic inflammatory diseases, including asthma [200,201]. In asthma, persistent airway inflammation, exposure to environmental oxidants, and activation of inflammatory cells lead to sustained ROS generation, rendering guanine bases particularly vulnerable to oxidation [181]. Among oxidatively modified DNA bases, 8-oxoG is one of the most abundant lesions detected in asthmatic patients. Elevated levels of 8-oxoG have been reported in airway epithelial cells, peripheral blood mononuclear cells, sputum, serum, urine, and bronchoalveolar lavage fluid, indicating widespread oxidative DNA injury in both local and systemic compartments [200,201]. Mechanistic studies further demonstrate that oxidative DNA damage in asthma is not merely a passive consequence of inflammation but actively contributes to disease pathogenesis. The excision of 8-oxoG by OGG1 initiates BER and releases free 8-oxoG, which forms a complex with cytoplasmic OGG1 and triggers downstream signaling pathways [202]. This OGG1–8-oxoG complex has been shown to activate small RAS and RHO family GTPases, leading to NF-κB activation and the transcription of pro-inflammatory genes involved in airway hyper responsiveness, epithelial remodeling, and immune cell recruitment. These findings highlight a dual role of 8-oxoG as both a marker of oxidative DNA damage and a mediator of pro-inflammatory signaling in asthma [201].

Clinical and epidemiological evidence supports the relevance of oxidative DNA damage to asthma severity and risk. In a large pediatric cohort study examining environmental exposure and OS, asthmatic children exhibited significantly higher urinary levels of oxidative DNA damage markers compared with healthy controls. Importantly, oxidative DNA damage was strongly associated with asthma risk; each unit increase in ln-transformed urinary oxidative DNA damage marker levels was linked to a markedly increased odds of asthma (odds ratio = 9.96, 95% CI: 4.75–20.9), demonstrating a robust statistical association between oxidative DNA injury and disease presence [169]. Although direct quantification of intracellular 8-oxoG in all clinical asthma phenotypes remains challenging, studies assessing systemic OS consistently show significantly elevated oxidative damage in asthmatic patients, particularly in those with poor disease control. Patients with uncontrolled asthma display significantly higher OS markers compared with controlled asthma and healthy subjects (*p* < 0.001), supporting the concept that sustained oxidative injury is closely linked to asthma severity and disease [63]. Collectively, these findings indicate that 8-oxoG is a central biomarker of oxidative DNA damage in asthma and plays an active role in amplifying inflammatory signaling through OGG1-mediated repair pathways. The accumulation and repair of 8-oxoG lesions reflect both the oxidative burden and the molecular processes driving chronic airway inflammation, positioning 8-oxoG as a mechanistically relevant marker and potential therapeutic target in asthma.

### 7.3. Protein Oxidation/Nitration Product

#### 7.3.1. Protein Carbonyl

Protein carbonylation is a widely used biomarker of protein oxidation, arising from the direct modification of amino acid side chains by ROS, which results in altered protein structure, impaired enzymatic activity, and loss of biological function. In asthma, chronic airway inflammation and sustained OS promote extensive protein oxidation, making protein carbonyls reliable indicators of oxidative damage and systemic oxidative burden [203,204]. Clinical studies consistently demonstrate elevated protein oxidation in asthmatic patients compared with healthy controls. In a prospective cohort study evaluating OS in adult asthma, plasma levels of advanced oxidation protein products (AOPPs), a closely related marker of protein carbonylation, were significantly higher in asthmatic patients than in controls (*p* < 0.001), indicating increased protein oxidative damage associated with the disease. Moreover, protein oxidative damage appears to correlate with asthma control and disease severity. The same study reported significantly higher AOPP levels in patients with uncontrolled asthma compared with those with controlled disease (*p* < 0.001), suggesting that enhanced protein carbonylation reflects heightened OS in more severe or poorly controlled asthma phenotypes [63]. At the mechanistic level, excessive ROS generated in the inflamed asthmatic airway can oxidatively modify circulating and tissue proteins, thereby amplifying inflammatory signaling, impairing antioxidant defenses, and contributing to airway remodeling. Protein oxidation products, including carbonylated proteins, are therefore not only markers of oxidative injury, but may also participate in the perpetuation of chronic inflammation in asthma [200,201].

These findings indicate that increased protein carbonylation is a hallmark of OS in asthma and is closely associated with disease presence and poor asthma control, supporting its utility as a biomarker of oxidative protein damage in asthmatic patients.

#### 7.3.2. 3-Nitrotyrosine

3-Nitrotyrosine (3-NT), formed by tyrosine nitration via RNS such as ONOO^−^, is a well-established biomarker of nitrosative stress. In asthma, increased nitrotyrosine reflects enhanced NO-derived oxidant activity within the airways and lung tissue. Hanazawa et al. reported significantly higher nitrotyrosine in exhaled breath condensate of steroid-naïve mild asthma compared with controls (15.3 ± 2.0 vs. 6.3 ± 0.8 ng/mL, *p* < 0.01), while levels were lower in steroid-treated moderate and severe asthma (*p* < 0.05), indicating the treatment-related modulation of nitrative stress [205]. Furthermore, immunofluorescence studies showed minimal nitrotyrosine staining in non-asthmatic lungs but significantly greater staining distributed across airways and parenchyma in asthmatic lungs, supporting widespread nitrative injury in asthma [206]. Proteomic analysis also identified extensive nitration of multiple airway proteins in experimental asthma, including CAT, with reduced CAT activity in asthmatic bronchoalveolar lavage fluid (*p* < 0.05), suggesting functional consequences of nitration that may amplify OS [207].

#### 7.3.3. Advanced Oxidation Protein Products

Advanced oxidation protein products (AOPPs) are dityrosine-containing, cross-linked protein fragments generated when plasma proteins (particularly albumin) undergo oxidative modification, largely through chlorinated oxidants such as HOCl produced by peroxidases (e.g., MPO). Because they reflect sustained oxidative modification of circulating proteins, AOPPs are considered reliable biomarkers of oxidative protein damage and systemic OS in asthma [208]. Clinically, increased AOPP has been linked to worse asthma status. In adult asthma, plasma AOPP levels were significantly higher in patients with severe asthma compared with those with moderate disease (*p* < 0.05), supporting an association between oxidative protein injury and disease severity [209]. However, the findings are not entirely consistent across populations. In a Tunisian case–control study, AOPP did not differ significantly between asthmatic patients and healthy controls (*p* = 0.98), highlighting variability that may relate to phenotype differences, treatment exposure, sample type, or baseline oxidative burden [65]. AOPPs remain a meaningful marker of oxidative protein damage in asthma, with evidence suggesting higher levels in more severe and poorly controlled disease, while also showing inter-study heterogeneity.

### 7.4. miRNAs as Novel Biomarkers of Oxidative Stress in Asthma

Recent evidence indicates that several asthma-related miRNAs reflect redox imbalance and oxidative/nitrosative pathways and may serve as accessible biomarkers (Table 7). In an ovalbumin (OVA)-induced asthma model, miR-182-5p was significantly downregulated and mechanistically linked to OS as it directly targets NOX4; restoring miR-182-5p reduced ROS generation and downstream inflammatory/mitochondrial injury [210]. In patients with asthma–chronic obstructive pulmonary disease (COPD) overlap, miR-125b-5p was upregulated and promoted OS and late apoptosis; importantly, miR-125b-5p small interfering RNA (siRNA) significantly reduced the percentage of ROS-producing cells (*p* < 0.05; *p* < 0.001 reported in the experimental system) [211]. Beyond these disease-model and translational findings, miRNA–ROS crosstalk has been tied to antioxidant signaling in asthma. In allergen-challenged mice, multiple miRNAs (including miR-155, miR-146a/146b, miR-144, miR-34a) were increased, while antioxidant intervention modulated miRNAs (notably miR-144 and miR-34a) alongside the restoration of Nrf2, supporting their relevance to oxidative-stress regulation in asthma [212].

In addition, the asthma miRNA literature identifies miRNAs with oxidative/inflammatory roles such as miR-21 (reported to promote OS in asthmatic models via signaling pathways), and highlights circulating/exosomal miRNAs as promising biomarkers across phenotypes [213].

## 8. Proposed Multilevel Redox-Based Prognostic Framework for Asthma Severity

The biomarkers reviewed herein are not intended to be evaluated in isolation, but rather as components of a multilevel redox prognostic panel—an integrated framework in which each tier contributes distinct, biologically non-redundant information about the patient’s redox status. The rationale for this multilevel approach rests on the complementary nature of the three core tiers: Tier I (genetic variants) captures the patient’s stable, inherited baseline redox capacity, reflecting predisposition to oxidant-driven airway injury that persists regardless of disease activity or treatment; Tier II (enzymatic activity) captures dynamic, modifiable systemic redox status, particularly the serum activities of antioxidant enzymes (SOD, CAT, GPx) and pro-oxidant enzymes (MPO, XO), that fluctuate with disease activity, exacerbation episodes, and therapeutic interventions; and Tier III (oxidative damage readouts) captures the cumulative downstream molecular consequences of redox imbalance, including lipid peroxidation products (8-isoprostane, MDA), DNA oxidation markers (8-oxodG), and protein oxidation indices (protein carbonyls, 3-nitrotyrosine), indexing the net injurious output of the system regardless of its enzymatic source [215,216]. No single tier captures all three dimensions: a patient with a high-risk genotype may have compensated enzymatic activity at rest, and a patient with depleted SOD activity may not yet show elevated oxidative damage markers if enzymatic compensation by GPx or Trx remains intact. Evidence from related chronic inflammatory diseases indicates that biomarker panel approaches integrating multiple complementary readouts outperform single-marker models in phenotype discrimination and severity stratification [217]. The proposed panel is summarized in Table 8 with its inputs, clinical outputs, and recommended measurement methods.

### 8.1. Evidence Prioritization: Key Redox Genetic Variants for the Proposed Framework

Three variants meet the threshold for priority inclusion in the proposed clinical panel, based on replication across independent cohorts, consistency of the genotype disease association, and direct relevance to severity rather than susceptibility alone. First, the combined GSTM1-null/GSTT1-null genotype has the strongest and most replicated genetic evidence in redox asthma research, supported by a meta-analysis of over 10,000 participants and multi-cohort severity data linking the combined null genotype to hospitalization risk (OR 1.51) and pollution-amplified exacerbation risk (OR 1.69) [5,118,120]. Second, GSTP1 Ile105Val (rs1695, val/val) is currently the only individual redox gene variant with independent multivariate prediction of asthma severity after adjustment for confounders, with an OR of 4.21 (95% CI 1.58–11.21) for severity classification in a pediatric cohort alongside a biological gradient in MDA and GSH levels [117]. Third, Nrf2/KEAP1 pathway variants have the most clinically actionable implication for the steroid-resistant endotype, linking constitutional antioxidant transcriptional control to HDAC2-mediated corticosteroid insensitivity, the mechanism with the greatest potential to change treatment decisions in GINA Step 4–5 patients [6,220,221]. Two variants were classified as secondary (moderate evidence): NOS2 (CCTTT)n repeat and rs10459953, which bridge the genetic and FeNO layers of the panel and have replicated associations with exacerbation frequency and FeNO levels [106,110,123,137], and MPO −463G>A (rs2333227), which has functional genotype–phenotype evidence in asthma and is particularly relevant to the non-allergic/neutrophilic endotype [105,140,141]. The remaining variants discussed in this section, SOD2 Val16Ala (rs4880), CAT rs1001179, GPx1 Pro198Leu (rs1050450), HO-1 (GT)n, and NQO1 Pro187Ser (rs1800566), are classified as exploratory. They were retained in the review because they are biologically plausible, mechanistically relevant to the oxidant/antioxidant framework, and have generated hypotheses warranting further study in severity-stratified cohorts. However, inconsistent replication across ethnic groups, predominance of susceptibility rather than severity evidence, and modest individual effect sizes currently preclude their inclusion as primary panel components in a clinical implementation context [88,89,90,92,135].

### 8.2. Asthma Severity Requires a Multibiomarker Approach Rather than Reliance on a Single Prognostic Marker

The limitations of single-biomarker approaches in asthma are not simply a matter of analytical imprecision; they reflect a deeper biological non-redundancy across the informational tiers that make up the redox system. Three distinct forms of non-redundancy justify the multilevel approach.

#### 8.2.1. Temporal Non-Redundancy

Genetic variants (Tier I) encode the patient’s fixed, constitutional redox capacity, a baseline that does not fluctuate with disease activity, treatment, or environmental conditions, and that therefore reflects long-term susceptibility to oxidant-driven injury. Enzymatic activities (Tier II) capture the patient’s current, dynamic redox state, a modifiable property that changes with exacerbations, corticosteroid treatment, and pollution exposure [63,64]. Oxidative damage products (Tier III) such as MDA, 8-isoprostanes, and 8-OHdG record cumulative, historical net injury: the molecular footprint of prior redox imbalance that persists even after an acute episode has resolved [164,166]. A patient may carry a high-risk GSTM1-null/GSTT1-null genotype (Tier I) but demonstrate compensated enzymatic activity at rest (Tier II) with low circulating MDA (Tier III). Each tier provides a genuinely distinct piece of clinical information; no single tier can substitute for the others.

#### 8.2.2. Mechanistic Non-Redundancy

The pathway from genotype to oxidative damage is not a fixed linear cascade but a system with multiple compensatory branch points. A patient with impaired mitochondrial antioxidant capacity due to SOD2 Val16Ala may upregulate cytosolic GPx or Trx to partially compensate, meaning that enzyme activity alone can underestimate the true downstream oxidative burden unless damage markers are measured directly [66,208]. Conversely, chronically elevated MDA without a corresponding drop in enzymatic activity may signal environmental or dietary oxidant overload rather than a primary enzymatic deficiency and distinction with direct therapeutic relevance.

#### 8.2.3. Endotype Discrimination Non-Redundancy

Tier II pro-oxidant markers, specifically MPO and XO activity, identify the neutrophilic/non-Type 2 endotype, a phenotype that is not captured by standard Type 2 biomarkers (FeNO, eosinophil count) and that is frequently steroid-resistant [87,147]. Tier III urinary or exhaled bromotyrosine identifies activated eosinophil peroxidase in the Type 2-high endotype. Tier I Nrf2/KEAP1 and HDAC2-linked variants identify steroid-resistance risk across both endotypes [6,220,221]. No single tier covers all three endotype-discriminating dimensions simultaneously, and it is the combination that approaches adequate phenotypic resolution.

These three forms of non-redundancy provide the biological rationale for why the tiers must be combined rather than treated as interchangeable alternatives. Clinically, this translates into two concrete decision-making scenarios. In a patient with severe, poorly controlled asthma, a Tier I screen for Nrf2/KEAP1 variants identifies a plausible mechanism of corticosteroid insensitivity; Tier II MPO activity confirms a neutrophilic oxidant-driven endotype missed by FeNO; and Tier III 8-isoprostane quantifies the cumulative oxidative burden and guides the urgency of antioxidant co-therapy. Without Tier I, the steroid-resistance mechanism is invisible; without Tier II, the non-Type 2 phenotype is missed; without Tier III, the severity of ongoing oxidative injury is unmeasured. In a newly diagnosed patient with mild asthma, a GSTT1-null genotype (Tier I) flags elevated exacerbation risk under pollution exposure, guiding early environmental counselling; baseline SOD and GPx activities (Tier II) establish the patient’s antioxidant reserve; and a normal 8-isoprostane (Tier III) confirms that no significant cumulative oxidative damage has yet accrued, with longitudinal Tiers II and III monitoring then enabling the early detection of reserve depletion before clinical deterioration. This framework is consistent with the emerging consensus that fully determining the phenotype or endotype of severe asthma requires the interpretation of combinations of biomarkers, and that panels of biomarkers improve the identification of asthma endotypes in the era of precision medicine [215,217].

### 8.3. Redox Biomarkers in Clinical Practice: Comparison with Established Markers and Proposed Workflow

Existing guideline-endorsed biomarkers for asthma, principally FeNO and blood eosinophil count, are valuable, well-validated, and clinically available, but they address a specific and limited dimension of airway pathobiology. Type 2 (eosinophilic) inflammation. FeNO reflects iNOS-driven nitric oxide production in eosinophilic airway inflammation and is a reliable indicator of ICS responsiveness in the Type 2-high endotype [23,24]. Blood eosinophils similarly identify Type 2 inflammation, predict exacerbation risk, and guide biologic therapy selection (e.g., anti-IL-5 agents) [13,14]. However, both markers are unreliable in non-eosinophilic (Type 2-low) asthma, which encompasses up to 50% of severe asthma, and neither captures the neutrophilic, oxidant-driven processes that characterize steroid-resistant disease [68,87]. Critically, FeNO and eosinophils provide no information about the patient’s antioxidant reserve, cumulative oxidative tissue injury, or genetic redox capacity, the three informational tiers that distinguish the multilevel redox approach from conventional biomarker assessment (Table 9).

Redox biomarkers do not substitute for FeNO or eosinophil measurements; rather, they expand the clinical interpretation of inflammatory and oxidative pathways by addressing complementary biological dimensions. In this integrated framework, FeNO and eosinophil counts primarily reflect Type 2 inflammatory activity and are most useful for predicting responsiveness to ICS and biologic therapies targeting Type 2 pathways. In contrast, enzymatic antioxidant markers such as serum SOD, CAT, and GPx provide a dynamic assessment of the patient’s current antioxidant capacity and the degree of functional depletion in redox defense systems. Oxidative damage biomarkers, including MDA, 8-isoprostane, and 8-OHdG, reflect the cumulative burden of lipid, protein, and DNA oxidative injury over time, thereby indicating the extent of ongoing molecular damage rather than inflammatory phenotype alone. In addition, genetic redox-related variants such as GSTM1/T1 null genotypes and Nrf2/KEAP1 polymorphisms represent inherited susceptibility factors that define baseline redox vulnerability and may contribute to inter-individual differences in antioxidant responsiveness and potential steroid resistance [6,66,208,220].

In terms of practical implementation, redox biomarkers can be incorporated into clinical assessment at three levels of complexity. At the basic level, which is feasible in most clinical laboratories, serum MDA and TAC can be measured from standard venous blood samples alongside routine inflammatory markers, and depleted TAC or elevated MDA can flag heightened oxidative burden in patients with poorly controlled asthma who have normal FeNO and eosinophil counts, precisely the non-Type 2 group for whom existing biomarkers give no guidance [63,64,164]. At the intermediate level, serum SOD, CAT, and GPx activities can be assayed by standard spectrophotometric methods available in specialist centers; abnormal enzyme activity in this context provides a mechanistic explanation for poor disease control and may justify antioxidant co-therapy trials or targeted environmental interventions [65,67,68]. At the advanced level, genetic screening for high-impact variants (*GSTM1/GSTT1* null, *GSTP1* rs1695, *NOS2* (CCTTT)n, *Nrf2/KEAP1* pathway) can be used once in a patient’s lifetime to establish constitutional redox risk, inform gene–environment counselling (e.g., secondhand smoke avoidance in GSTT1-null patients), and identify candidates for future redox-targeted therapies [117,118,120,220].

A key practical limitation distinguishing redox markers from FeNO and eosinophils is the current absence of standardized reference ranges, validated decision thresholds, and point-of-care assay platforms. Until prospective validation studies establish clinically actionable cut-offs, analogous to the FeNO ≥ 25 ppb threshold for predicting ICS response, redox biomarkers should be interpreted as adjunctive tools that add mechanistic context to standard phenotyping rather than as independent diagnostic criteria [219,222,223]. The development of such thresholds in well-phenotyped prospective cohorts represents the most important translational research gap in this field.

**Table 9:** Clinical integration of asthma biomarkers.

Biomarker	Clinical Question Addressed	Endotype Covered	Specimen	Clinical Availability	Key Limitation	Key References
**FeNO**	ICS responsiveness; Type 2 inflammation activity	Type 2-high (eosinophilic)	Exhaled breath	Widely available (point-of-care)	Unreliable in non-eosinophilic/steroid-treated patients	[23,24,224]
**Blood eosinophils**	Type 2 burden; biologic therapy eligibility	Type 2-high	Venous blood (CBC)	Universal	Fluctuates with ICS use; absent in non-Type 2 asthma	[13,225,226]
**Serum SOD/CAT/GPx activity**	Current antioxidant reserve; dynamic redox state	Both endotypes, especially Type 2-low	Venous blood	Specialist laboratory	No validated reference ranges; influenced by diet/cofactors	[225,226]
**MDA/8-isoprostane**	Cumulative oxidative damage; lipid peroxidation burden	Both endotypes	Serum/urine/EBC	Research setting; some specialist labs	High inter-laboratory variability; method-dependent	[165,224,227,228,229]
**8-OHdG/protein carbonyls**	DNA/protein oxidative injury; disease chronicity	Both endotypes	Urine/serum	Research setting	Limited standardization; ELISA vs. HPLC values differ; not yet clinical routine	[169,170,201,230]
**Genetic variants: *GSTM1/T1*, *GSTP1*, *Nrf2/KEAP1*, *NOS2***	Constitutional redox capacity; steroid-resistance risk; gene–environment susceptibility	Both endotypes	Blood DNA	Research/specialist genomics	Single-variant effect sizes small; no clinical algorithm yet	[5,88,106,117,118,120,220]

Abbreviations: FeNO, fractional exhaled nitric oxide; ICS, inhaled corticosteroids; CBC, complete blood count; SOD, superoxide dismutase; CAT, catalase; GPx, glutathione peroxidase; MDA, malondialdehyde; 8-OHdG, 8-hydroxy-2′-deoxyguanosine; EBC, exhaled breath condensate.

### 8.4. Clinical Translation Opportunities—Toward Prevention and Personalized Therapy

Beyond their diagnostic utility, OS and antioxidant defense biomarkers carry actionable implications for asthma prevention and individualized therapeutic management. The following candidates represent the highest-priority parameters for prospective clinical validation, selected on the basis of mechanistic plausibility, evidence of severity-correlation, and potential modifiability.

#### 8.4.1. Serum GPx and SOD as Dynamic Monitoring Biomarkers for Exacerbation Risk

Among the enzymatic antioxidants, serum GPx and SOD activity have demonstrated consistent severity-dependent depletion across independent cohorts, making them strong candidates for longitudinal monitoring. Comhair et al. tracked serum SOD activity over four years in 47 adults with asthma and found that baseline SOD was a significant predictor of subsequent airflow decline, with the lowest SOD values in patients who later developed the most severe obstruction [231]. Katsoulis et al. further demonstrated that erythrocyte SOD activity dropped sharply during acute exacerbations (43.6 ± 31.8 vs. 96.2 ± 54.1 units/mL at discharge, *p* < 0.001) and correlated with admission FEV1 (r = 0.57, *p* < 0.001) and arterial oxygen tension, indicating that SOD tracks real-time disease severity rather than merely identifying asthmatic status [226]. Abboud et al. confirmed that serum GPx and SOD, but not salivary SOD, differentiated adult from childhood asthma and tracked disease severity across groups (*p* < 0.05) [232]. Al-Kinani and Sayyah corroborated that declining GPx and CAT activities, contrasted with elevated SOD, aligned with increasing GINA severity steps in a severity-stratified cohort [233]. Collectively, these data suggest that serial measurement of serum GPx/SOD activity at clinic visits could serve as an inexpensive, blood-based adjunct for exacerbation risk stratification, though validated reference ranges and prospective outcome-linked thresholds remain to be established [208].

#### 8.4.2. GSTM1/GSTT1-Null Genotypes: Identifying Patients at Highest Risk from Environmental Oxidant Exposures

GSTM1- and GSTT1-null genotypes, present in approximately 50% of the general population, abolish the capacity of the corresponding glutathione S-transferase enzymes to conjugate and detoxify reactive electrophiles derived from air pollutants, ozone, and tobacco smoke. This constitutional vulnerability has direct preventive implications. Palmer et al., in the BREATHE cohort of 504 young asthmatics, demonstrated that GSTM1-null adolescents (13–21 years) from tobacco-exposed households had peak expiratory flow rates approximately 15% lower than GSTM1-intact peers from the same environment (83% vs. 98% predicted, *p* < 0.05), an effect absent in unexposed children, confirming that the null genotype amplifies the lung-function consequence of environmental smoke exposure rather than merely conferring baseline susceptibility [118]. Kabesch et al. extended this finding in 3054 German schoolchildren, showing that GSTM1-null children currently exposed to environmental tobacco smoke had a 5.5-fold elevated risk of current asthma (OR 5.5, 95% CI 1.6–18.6) compared with GSTM1-intact unexposed peers [234]. Turner et al. (2016), in a multi-cohort study of 2197 children with asthma across four populations (BREATHE, GALA II, PACMAN, PAGES), specifically identified GSTT1-null status as associated with a 32% increased odds of exacerbation (OR 1.32, 95% CI 1.02–1.71, *p* = 0.033), an effect that rose to 69% increased odds (OR 1.69, 95% CI 1.02–2.73) among GSTT1-null children additionally exposed to secondhand smoke [235]. These gene-by-environment interactions provide a compelling argument for clinical genotyping: identifying GSTM1/GSTT1-null patients would allow clinicians and public health providers to deliver targeted environmental avoidance counseling and prioritize monitoring in high-pollution or tobacco-exposed contexts.

#### 8.4.3. EBC 8-Isoprostane as a Non-Invasive Phenotyping Tool

EBC 8-isoprostane, a stable prostaglandin-like compound generated by free radical-catalyzed peroxidation of arachidonic acid, offers a fully non-invasive window into airway OS. Samitas et al. demonstrated progressive elevation of EBC 8-isoprostane across GINA severity categories, mild: 49.1 ± 5.2 pg/mL; moderate: 49.7 ± 5.2 pg/mL; severe: 77.7 ± 7.3 pg/mL, with severe levels significantly exceeding those of mild/moderate patients (*p* < 0.01) and all asthmatic levels significantly exceeding the healthy controls (16.4 ± 1.6 pg/mL, *p* < 0.001) [228]. A 2024 study by Woo et al. added a phenotyping dimension by showing that urinary 8-isoprostane was significantly higher in non-eosinophilic (T2-low) asthma than in eosinophilic (T2-high) asthma (*p* < 0.05), with an AUC of 0.678 for discriminating non-eosinophilic disease, and that it correlated with neutrophilic inflammation markers and airway remodeling, precisely the phenotype that FeNO fails to characterize [186]. Barreto et al. further showed that baseline EBC 8-isoprostane (but not FeNO) predicted exercise-induced bronchoconstriction severity in asthmatic children (r = −0.47, *p* = 0.002), illustrating a complementary rather than redundant role relative to established markers [236]. The primary barrier to clinical implementation remains methodological: a systematic review and meta-analysis by Peel et al. identified substantial inter-laboratory heterogeneity (I^2^ = 94%) across 20 studies, underscoring the need for standardized collection and assay protocols before 8-isoprostane can be introduced as a clinical phenotyping tool analogous to FeNO [4].

#### 8.4.4. Selenium and Zinc as Modifiable Cofactors: Targeted Supplementation in Deficient Patients

Se is an essential cofactor for GPx isoenzymes; Zn is required for SOD1 activity and metallothionein-based redox buffering. Both are consistently found at lower levels in asthmatic patients compared with healthy controls, and both are theoretically modifiable through dietary supplementation. The evidence for supplementation is promising but context-dependent. Shaheen et al. conducted the largest RCT of Se supplementation in adults with asthma (*n* = 197, 24 weeks, 100 µg/day Se yeast), and while no significant improvement was observed in the primary outcome (quality of life score) or secondary lung function measures in the overall cohort, 75% of participants were already taking ICS, a factor that may have blunted any measurable oxidant-driven impairment [237]. More encouraging results emerged from studies in Se-deficient populations: Voicehovska et al. reported that 200 µg/day organic Se for 16 weeks in asthmatic patients with confirmed Se deficit produced a significant increase in GPx activity (38.6 ± 10.7 to 58.6 ± 14.6 U/g Hb, *p* = 0.01) and normalization of chemiluminescence-based OS markers [238]. A pediatric RCT by Patil and Patil found that Se supplementation per RDA for 6 months in children aged 1–12 significantly reduced wheezing episodes (52% of Se group had <5 episodes vs. 19% in placebo, *p* < 0.05) and hospitalizations [239]. A systematic review of six eligible RCTs concluded that while evidence is heterogeneous, Se supplementation shows a clinically meaningful signal particularly in patients with documented deficiency [239]. The clear implication is that supplementation should be restricted to patients with confirmed Se or Zn insufficiency, a prerequisite that requires the pre-treatment measurement of plasma Se and erythrocyte Zn, a practice not yet standard in asthma clinics.

#### 8.4.5. Nrf2 Pathway Variants as Candidate Predictors of Corticosteroid Resistance

The Nrf2–KEAP1 axis governs the transcriptional induction of over 200 antioxidant and phase II detoxification genes, including *HO-1*, *NQO1*, *GCLC*, and *Trx* reductase. Impaired Nrf2 signaling, whether from loss-of-function variants, KEAP1 gain-of-function, or promoter hypermethylation, reduces the airways’ adaptive antioxidant response and may independently contribute to the corticosteroid-insensitive oxidant burden seen in severe asthma. Cho et al. identified functional Nrf2 promoter polymorphisms that alter transcriptional activity and modify OS response, providing a genetic mechanism through which Nrf2 variant carriers may be constitutionally more vulnerable to redox-driven steroid resistance [220]. The clinical rationale is reinforced by the established mechanism of ICS insensitivity: oxidative and nitrative stress inactivate histone deacetylase 2 (HDAC2), a transcriptional co-repressor required for corticosteroid action, and reduced Nrf2 activity amplifies this inactivation by failing to neutralize the oxidants responsible [208]. While no prospective trial has yet validated Nrf2 genotyping as a steroid-resistance predictor in asthma, the pathway represents a high-priority candidate for pharmacogenomic investigation, particularly as Nrf2 activators (e.g., sulforaphane, dimethyl fumarate) enter early-phase testing in airway diseases.

## 9. Challenges of Using Oxidative and Antioxidant Biomarkers in Predicting Severity of Asthma

OS occurs when the body produces an excess of ROS that surpasses its capacity to eliminate them through antioxidants. Inflammation, allergen exposure, and environmental factors such as air pollution can induce OS in individuals with asthma. This results in injury to the airway epithelium, increased mucus production, bronchoconstriction, and chronic inflammation. Lack of specificity and standardization for numerous OS markers, such as protein carbonyls and MDA, are elevated not just in asthma but also in other inflammatory conditions such diabetes and atherosclerosis [222]. Methods for quantifying OS that utilize analytical heterogeneity often lack specificity, require substantial effort, or are overly sophisticated for routine clinical use [222]. Measuring reactive oxygen species such as H_2_O_2_ in serum is challenging and may not always be feasible in real-time [164]. The fluctuating serum levels of oxidants are influenced by environmental factors, such as tobacco smoke and pollution, as well as the use of medications such as ICS, thus confounding the determination of a reliable diagnostic threshold [240]. MDA and other oxidative markers are frequently elevated in individuals with asthma; however, they are not limited to this condition and can be influenced by factors such as diet, infections, and environmental pollutants. This complicates their use as precise indicators of asthma severity or control [178].

Markers of OS demonstrate significant variability due to individual differences in nutrition, genetics, and environmental exposure [241]. This variability complicates the ability of studies to yield consistent results repeatedly. Diverse methodologies for quantifying OS, such as HPLC and ELISA for MDA, may yield varying results. Comparing research is challenging due to the absence of standardized methodologies for these assessments. Corticosteroids and other pharmacological agents employed in asthma management can alter OS markers, thereby obscuring the preexisting oxidative imbalance in patients [223].

Markers of OS fluctuate based on the disease state, treatment regimen, and the presence of coexisting conditions, including infections. During acute asthma exacerbations, signs of OS may increase; however, this is not consistently a reliable assessment of long-term asthma management [242] (Table 10).

### 9.1. Challenges in Translating Gene Polymorphisms into Clinical Practice

Asthma is a multifaceted disease affected by over 100 potential genes; nevertheless, pinpointing actionable genetic indicators continues to be difficult. A major hurdle in asthma genetics is the difficulty in replicating findings across several ethnic groups. Many preliminary studies were limited by insufficient sample sizes or failed to account for environmental interactions [248].

Asthma is not a distinct condition; it encompasses multiple phenotypes, including eosinophilic, neutrophilic, and early-onset variants. A polymorphism like TLR4 may show no correlation when assessed in a wide cohort, however, it may possess importance exclusively in specific environmental circumstances (urban versus rural) [240]. Certain gene polymorphisms, including *IL-4* and *STAT6*, augment susceptibility; nevertheless, they often yield only a marginal increase in risk (e.g., 45–47%), which is insufficient for individual clinical diagnosis or therapeutic adjustments [248]. Asthma is influenced by both environmental and genetic factors. Several gene variations linked to OS enzymes have been identified as potential contributors to asthma susceptibility [249].

Variants such as *GSTM1*, *GSTP1*, and *GSTT1* have been shown to alter the body’s capacity to combat oxidative damage, potentially exacerbating asthma [250,251]. Nrf2 is an essential transcription factor involved in the regulation of antioxidant enzymes. Polymorphisms in *Nrf2* have been associated with altered responses to OS in asthma [220,221]. In addition, the CAT gene encodes catalase, an enzyme responsible for degrading hydrogen peroxide (H_2_O_2_). Polymorphisms in CAT have also been linked to asthma severity [208].

Despite these findings, the clinical significance of these polymorphisms remains ambiguous. The interpretation of these data is hampered by minimal impact sizes, disparities among ethnic groups, and gene–environment interactions. Gene–environment interactions, such as exposure to pollutants or allergens, may influence the expression of OS genes, hence challenging the generalization of findings across various populations.

A primary obstacle for the therapeutic application of OS markers is the considerable heterogeneity of asthma. Asthma endotypes have distinct inflammatory and molecular characteristics that may influence the expression and predictive value of biomarkers. Moreover, OS markers are affected by numerous confounding variables, including age, gender, obesity, smoking, dietary practices, environmental pollution, medication usage, and concurrent illnesses. Consequently, a singular oxidative biomarker may not reliably forecast disease severity across all patient demographics [66,252].

Genetic polymorphisms in genes that encode antioxidant enzymes (GSTM1, GSTT1, GSTP1, SOD2, CAT, GPX1, and PON1) may influence individual susceptibility to oxidative damage and therapeutic efficacy [253]. Nonetheless, the predictive significance of genetic variants in isolation is frequently constrained, as environmental factors and epigenetic processes considerably affect gene expression and enzyme activity. Consequently, the genotypic data must be linked with biochemical and clinical indicators [100].

Many gene variants linked to asthma exhibit weak relationships with disease severity, hence limiting their clinical applicability as biomarkers for diagnosis or prognosis [254]. Genetic variants may exert varying effects among populations, and environmental factors, such as air pollution or smoking, may modify these effects [255]. This challenges the translation of findings into universally applicable evaluations. Polymorphisms in OS genes are associated with asthma; however, the precise biochemical mechanisms by which these genetic variants affect the disease remain unclear [256].

A multilevel redox-based prognostic model may surpass the efficacy of any singular biomarker, based on the available evidence. The model would integrate genetic susceptibility markers (e.g., *GST*, *GPX1*, *CAT*, *SOD2*, and *PON1* polymorphisms), serum antioxidant enzyme activities (SOD, CAT, GPx, GST, and PON1), oxidative damage products (MDA, 8-isoprostane), inflammatory biomarkers (eosinophil count and FeNO), and clinical indicators (FEV1, asthma control scores, exacerbation frequency, and corticosteroid responsiveness). This cohesive technique may result in enhanced patient classification, prompt the identification of high-risk individuals, and tailored treatment protocols [250,252].

From both preventive and therapeutic standpoints, redox biomarkers may aid in identifying individuals at elevated risk of severe disease, thereby facilitating the implementation of environmental control measures, reduction in pollutant exposure, smoking cessation initiatives, and personalized therapeutic strategies. Moreover, assessing OS markers could aid in identifying patients who would benefit from adjunctive antioxidant therapy or certain biologics. Future directions encompass large-scale prospective research, biomarker standardization, and the validation of integrated prediction models before widespread clinical implementation [55,208,243].

### 9.2. Challenges of Serum Enzyme Cofactors

Enzymes such SOD, CAT, and GPx necessitate particular metal ions or molecules (cofactors) such as NADPH for their activity [208]. The concentrations of enzymes and cofactors in peripheral blood may not correctly represent the elevated OS present locally in the airway epithelial lining fluid [164]. Numerous cofactors are obtained via dietary sources, including vitamins and carotenoids. This brings considerable “noise” into clinical data, as a patient’s dietary state may obscure the fundamental molecular dysregulation of asthma itself [245,257]. The clinical application is impeded due to cofactors frequently functioning in pairs (e.g., NAD+/NADH or GSH/GSSG). Assessing a single component does not yield a comprehensive understanding of the cell’s reducing potential [258].

Specific serum enzyme cofactors, such as vitamins C, E, and Se, are essential for the function of antioxidant enzymes. Deficiencies in these cofactors can intensify OS and inflammation in asthma. Vitamin C and Vitamin E are vital antioxidants that counteract free radicals. Their serum concentrations can directly affect the activity of antioxidant enzymes, including SOD and GPx [259]. Se serves as a cofactor for glutathione peroxidase, an essential antioxidant enzyme in the body [260].

The concentrations of these cofactors may vary significantly based on dietary intake and quantity consumed. However, variations in diet and supplements may complicate the analysis of OS indicators. A patient with adequate diet may exhibit normal levels of antioxidant enzymes, but an individual with a deficiency may experience heightened OS despite having identical underlying pathophysiology. This renders serum cofactor levels less pertinent for the management of asthma in routine clinical practice [261].

### 9.3. Clinical Translation and Application in Asthma

#### 9.3.1. Lack of Robust Correlation with Clinical Outcomes

Despite significant interest in OS and genetic biomarkers in asthma, no definitive clinical applications have been realized. For instance, OS markers do not consistently correlate with clinical outcomes such as asthma severity, lung function (e.g., FEV1), or symptom control. This limits their potential as reliable biomarkers [168]. Genetic testing for polymorphisms in OS genes has not yet been integrated into clinical practice, as the associations are weak and not actionable in a routine clinical setting [262]. Measurements of enzyme cofactors reveal inconsistent associations with asthma severity, rendering them less useful for therapeutic decision-making [263].

#### 9.3.2. Challenges with Standardization and Costs

The assays used to measure OS markers, gene polymorphisms, and serum cofactor levels vary between laboratories. This complicates the comparison of data across various studies or clinical scenarios [223]. The sophisticated techniques utilized to evaluate OS and genetic markers (e.g., HPLC, PCR, gene sequencing) are expensive and not typically available in regular clinical settings, hence limiting their application in resource-limited contexts [264].

## 10. Conclusions

This review demonstrates that OS is not merely a by-product of airway inflammation in asthma but a mechanistically embedded driver of disease severity, progression, and treatment responsiveness. The evidence supports a multilevel redox model in which asthma outcomes are shaped by the interplay of (i) upstream oxidant enzyme activity from NADPH oxidases, XO, and MPO; (ii) downstream antioxidant enzymatic defense through SOD, CAT, GPx, PRDXs, and the Trx system; (iii) inherited variation in both oxidative and antioxidative enzyme genes, including *GSTM1/GSTT1* null genotypes, *SOD2* Val16Ala, *CAT* rs1001179, *NOS* polymorphisms, and *Nrf2/KEAP1* pathway variants; and (iv) redox-sensitive regulatory networks, particularly the Nrf2–antioxidant response element axis and HDAC2-mediated steroid responsiveness pathways.

Serum enzyme activities of SOD, CAT, and GPx serve as dynamic, non-invasive indicators of systemic redox imbalance, with consistent evidence showing their depletion in poorly controlled and severe asthma, particularly during acute exacerbations. Oxidative damage biomarkers, including MDA, 8-isoprostanes, 8-oxodG, protein carbonyls, 3-nitrotyrosine, and AOPPs, provide complementary readouts of lipid, DNA, and protein oxidation that correlate with disease activity. Genetic polymorphisms in redox-related genes further explain interindividual variability in OS profiles, disease trajectories, and environmental susceptibility, reinforcing the concept that redox capacity is partly genetically determined. Micronutrient cofactors including Se, Zn, and Mg provide essential biochemical context for interpreting enzymatic biomarker data, while emerging evidence on OS-related miRNAs adds a novel regulatory dimension to redox-based asthma assessment. However, significant challenges remain in translating these findings into routine clinical practice, including biomarker non-specificity across inflammatory diseases, lack of standardized assays and reference ranges, confounding by diet and medication, and the modest individual effect sizes of genetic variants. Future efforts should focus on the development and validation of integrated, multilevel redox panels that combine high-impact genetic variants (e.g., *GSTM1/T1* null, CAT rs1001179), systemic enzymatic activity (e.g., serum SOD, GPx), and non-invasive markers of oxidative damage (e.g., EBC 8-isoprostane) within well-phenotyped, prospective asthma cohorts. Such panels, interpreted alongside micronutrient status and emerging miRNA signatures, hold promise for improved asthma phenotyping, severity prediction, identification of steroid-resistant endotypes, and ultimately, personalized redox-targeted therapeutic strategies.

## Figures and Tables

**Table 1:** Serum oxidative and antioxidant biomarkers as prognostic indicators in asthma severity.

Category	Biomarker	Direction in Asthma	Clinical/Prognostic Significance	Key Source
Antioxidants	SOD	Decreased (↓)	Indispensable first line of defense; strong positive correlation with FEV1 (r = 0.447).	[64,65,66]
	CAT	Decreased (↓)	Secondary defense; plummets during acute exacerbations to signal total exhaustion.	[54,64]
	GPx	Decreased (↓)	Predictor of poor disease control, acute crisis, and airway hyper responsiveness.	[63,67]
Oxidants	NOX2	Increased (↑)	Catalyzes the “oxidative burst” by converting molecular oxygen to superoxide anions (O_2_^•−^).	[58,68]
	MPO	Increased (↑)	Neutrophilic enzyme leading to the production of hypochlorous acid.	[59]

Abbreviations: CAT: catalase; FEV1: forced expiratory volume in 1 s; GPx: glutathione peroxidase; MPO: myeloperoxidase; NOX2: NADPH oxidase 2; SOD: superoxide dismutase.

**Table 2:** Single nucleotide polymorphisms (SNPs) and functional variants in oxidative and antioxidant genes linked to asthma.

Gene (Pathway)	Variant (rsID)	Variant Type/Effect on Function	Reported Asthma Outcome(s)	Evidence Type	References
***GSTM1***	GSTM1 null (deletion)	Loss of enzyme activity (no GSTM1)	Asthma susceptibility; stronger effects in some subgroups/exposures	Updated meta-analysis	[5]
***GSTT1***	GSTT1 null (deletion)	Loss of enzyme activity (no GSTT1)	Asthma susceptibility; gene–environment interactions	Updated meta-analysis	[5]
***SOD2***	rs4880 (Val16Ala)	Alters mitochondrial targeting/processing; modifies antioxidant capacity	Association with asthma risk reported; oxidant-defense modifier in pollution contexts	Meta-analysis; G × E cohort evidence	[90,91]
***CAT***	rs1001179 (−262C>T)	Promoter variant influencing CAT expression/activity	Childhood asthma and oxidative damage markers; new-onset asthma risk modified by ozone/community exposure	Case–control; cohort G × E	[92,93]
***CAT***	rs7943316 (A>T)	Promoter-region variant influencing CAT gene expression	Childhood asthma susceptibility	Association study	[94,95]
***HO-1***	(GT)n promoter repeat	Repeat length affects inducibility of HO-1	New-onset asthma risk modified by ozone exposure; ethnicity-specific effects reported	Prospective cohort	[93]
***HO-1***	rs2071747	Regulatory variant influencing HO-1 expression	No significant independent association; shows gene–gene interaction (e.g., with GSTP1, EPHX1) in childhood asthma	Association study (interaction-based)	[94,96]
***NQO1***	rs1800566 (C609T)	Reduced enzyme stability/activity	Asthma susceptibility signals in exposure-stratified analyses; childhood asthma associations in some cohorts	Family-based/exposure-stratified; case–control	[97]
***NQO1***	rs2917666	Variant in an antioxidant defense gene involved in redox protection	Asthma prevalence and new-onset asthma in interaction with traffic-related air pollution (NO_2_)	Gene–environment interaction study	[98]
***GSTP1***	rs1695 (Ile105Val)	Alters substrate affinity/catalytic efficiency	Asthma risk/phenotypes; ROS-related polymorphism studied in asthma contexts	Association studies; ROS-related polymorphism analysis	[99]
***Antioxidant defense enzyme gene set***	Multiple SNPs (34 genes/46 SNPs)	Pathway-level antioxidant variation	Associations and gene–gene interactions reported for adult asthma phenotypes	Pathway-wide association study	[100]
***SOD3***	rs1799895 (R213G)	Alters ECM affinity & localization	Shifts extracellular buffering; linked to airway remodeling	Multidisciplinary, translational research study	[101]
***GPX1***	rs1050450 (Pro198Leu)	Lower GPx1 activity	Candidate modifier of oxidative burden (often Se-dependent)	Case–control study	[102]

Abbreviations: CAT, catalase; ECM, extracellular matrix; GPX1, glutathione peroxidase 1; GST, glutathione S-transferase; GSTM1, glutathione S-transferase mu 1; GSTP1, glutathione S-transferase pi 1; GSTT1, glutathione S-transferase theta 1; G × E, gene–environment interaction; HO-1, heme oxygenase-1; NQO1, NAD(P)H quinone dehydrogenase 1; ROS, reactive oxygen species; SNP, single nucleotide polymorphism; rsID, reference SNP cluster identifier; SOD2, mitochondrial manganese superoxide dismutase; SOD3, superoxide dismutase 3.

**Table 3:** SNPs and functional variants in oxidative genes linked to asthma.

Enzyme	Gene	Chromosomal Location	Key Polymorphism(s)	Association with Asthma	Key Studies
***nNOS***	NOS1	12q24.2	Intragenic microsatellite repeats; Exon 29 SNP; Intronic (AAT)n repeat	Significantly associated with asthma in British and European populations (OR ~2.08). The (AAT)n repeat correlates with exhaled NO levels in asthmatics.	[103,104,105]
***iNOS***	NOS2	17q11.2-q12	rs10459953 (5′UTR); Ex16 +14C>T (rs2297518)	rs10459953 associated with childhood atopic asthma in Polish children (*p* = 0.0006). The Ex16 +14C>T T allele is more frequent in asthmatics (OR 3.34).	[106,107]
***iNOS***	NOS2	17q11.2–q12	Intron 4 (GT)n repeat (AFM311ZB1)	Associated with asthma severity; allele 3 was linked to greater severity, higher blood eosinophils, and higher serum NO levels in a family-based study.	[108]
***eNOS***	NOS3	7q35-36	Glu298Asp (rs1799983); Intron 4 VNTR; −786T>C	Most studies reported no significant association with asthma. A Czech study and Chinese study found no clear link. The 2023 meta-analysis appears to confirm NOS1 but not NOS3 as a risk gene.	[109,110,111]
***MPO***	MPO	17q23.1	−463G>A (rs2333227)	The −463A allele is protective in a Russian cohort (OR 0.64 for asthma), particularly for late-onset atopic asthma (OR 0.47). A Portuguese study found the opposite, with allele A more frequent in asthmatics. A Czech pilot found no association. Results are inconsistent across populations.	[105,112]
***NADPH oxidase*** **(p22phox subunit)**	CYBA	16q24.3	C242T (H72Y, rs4673); A640G (rs1049255); −930A/G	A640G heterozygotes showed reduced asthma risk in a Russian cohort (OR 0.66). Haplotype analysis in a Czech cohort found that the −930G/242T/640A haplotype increased asthma risk (OR 1.43). A 2025 study linked NOX2 gene expression (not SNPs) to atopic asthma severity.	[113,114]
***XOR***	XDH	2p23.1	Multiple SNPs (rs206805, rs185925, rs561525, rs2163059, rs1884725, rs4952085, etc.); I703V (rs17011368)	No direct asthma association studies found. XDH variants have been linked to sepsis/ARDS risk and hypertension, and XO-mediated OS exacerbates pulmonary inflammation in obese mice exposed to air pollution. The link to asthma remains indirect and speculative.	[115,116]

Abbreviations: CYBA, cytochrome b-245 alpha polypeptide; eNOS, endothelial nitric oxide synthase; iNOS, inducible nitric oxide synthase; MPO, myeloperoxidase; nNOS, neuronal nitric oxide synthase; NO, nitric oxide; NOS1, nitric oxide synthase 1; NOS2, nitric oxide synthase 2; NOS3, nitric oxide synthase 3; OR, odds ratio; OS, oxidative stress; SNP, single nucleotide polymorphism; VNTR, variable number tandem repeat; XDH, xanthine dehydrogenase; XOR, xanthine oxidoreductase.

**Table 4:** Redox-related genetic variants in asthma: Classification by primary association (susceptibility risk vs. disease severity).

Gene/Variant	Primary Association	Severity Evidence	Severity Link	Suggested Column Entry	Citation
***GSTM1*** **null**	Risk (susceptibility)	Indirect: GSTM1-null interacts with tobacco smoke to reduce peak expiratory flow in adolescents with asthma; GSTP1 val/val (but not GSTM1 null alone) independently predicts asthma severity by multivariate logistic regression.	Weak/Gene × Environment	Risk; G × E severity modifier	[117,118]
***GSTT1*** **null**	Risk (susceptibility) + Severity modifier	GSTT1 null is associated with increased exacerbation risk (OR 1.32, 95% CI 1.02–1.71); merged GSTM1/GSTT1 null increases risk of asthma-related hospital admissions (OR 1.51). GSTT1 null combined with second-hand smoke raises exacerbation OR to 1.69.	Moderate, exacerbation frequency, hospital admission	Risk + Severity (exacerbation risk)	[119,120]
***GSTP1*** **Ile105Val (rs1695)**	Risk + Severity	Val/Val genotype independently associated with severity by multivariate logistic regression (OR 4.21); val/val children have significantly higher MDA and lower GSH than other genotypes, correlating with oxidative injury severity gradient.	Strong, independent severity predictor in JACI cohort	Risk + Severity (OS severity gradient)	[117]
***SOD2*** **Val16Ala (rs4880)**	Risk (susceptibility)	SOD2 rs4880 associated with FVC and FEV_1_ decline in COPD (cross-disease evidence); no direct severity-stratified asthma RCT data in retrieved sources. One study found no significant SOD2 polymorphism association with asthma risk or severity in waterpipe smokers.	Weak/indirect (lung function decline across airway diseases)	Risk; Severity, indirect/exploratory	[95,121]
***CAT*** **rs1001179 (−262C>T)**	Risk (susceptibility)	Polymorphisms in antioxidant enzymes (CAT, SOD, GPx) are candidates for asthma susceptibility; functional polymorphisms in SOD and CAT investigated in Hong Kong Chinese asthmatics for susceptibility, severity data limited in retrieved abstracts.	Weak, susceptibility focus; severity data not established	Risk; Severity, not established	[122]
***NOS2*** **polymorphisms ((CCTTT)n repeat; rs10459953)**	Risk + Severity (exacerbation)	NOS2 (CCTTT)n pentanucleotide repeat contributes to varying mRNA expression and affects asthma exacerbations directly. NOS2 rs10459953 significantly associated with childhood asthma susceptibility; NOS2 intron 16+88G>T allele T more frequent in uncontrolled asthma (OR 2.9 trend, *p* = 0.057).	Moderate, exacerbation frequency; disease control	Risk + Severity (exacerbation; disease control)	[106,112,123]
***MPO*** **−463G>A (rs2333227)**	Risk + Severity/Endotype	MPO −463G>A associated with differential MPO plasma levels in asthmatics by genotype (GG genotype: 40.3 ± 37 ng/mL vs. AG: 16.5 ± 13.7 ng/mL; *p* = 0.009); allele A more frequent in asthmatics vs. controls (52.2% vs. 28%; *p* < 0.001). Non-allergic asthmatics have higher MPO levels, suggesting endotype-specific severity relevance.	Moderate, plasma MPO levels by genotype; endotype (allergic vs. non-allergic) discrimination	Risk + Severity (endotype/MPO activity level)	[112]
***HO-1*** **(GT)n microsatellite**	Risk (susceptibility) + Severity (indirect)	Long (GT)n repeats associated with reduced HO-1 inducibility and greater oxidative burden; classified as susceptibility in most studies but mechanistically linked to severity via impaired Nrf2-HO-1 antioxidant response in severe phenotypes. No direct severity-stratified RCT data in retrieved abstracts.	Indirect, mechanistic severity relevance via impaired antioxidant inducibility	Risk; Severity, mechanistic/exploratory	[89]
***NQO1*** **rs1800566 (Pro187Ser)**	Risk (susceptibility)	NQO1 Pro187Ser results in an unstable protein with reduced enzyme activity; strong susceptibility evidence from air-pollution G × E studies; limited direct severity-stratified data in retrieved abstracts.	Weak/G × E susceptibility; severity not directly established	Risk; G × E severity modifier (pollution exposure)	[124]
***Nrf2/KEAP1*** **variants**	Severity (steroid-resistance)	Nrf2 pathway variants are mechanistically linked to steroid-resistance in severe asthma via HDAC2 axis; bioinformatics study identifies glucocorticoid receptor (GR) signaling pathway as the most enriched common pathway in both moderate and severe asthma, with differential enrichment between phenotypes.	Strong mechanistic, steroid-resistance; moderate-to-severe discrimination	Severity (steroid-resistance; moderate–severe discrimination)	[125,126]
**Polygenic risk score (multi-locus)**	Risk + Severity	Polygenic risk scores significantly higher in difficult-to-control vs. easy-to-control asthma (*p* = 0.02); associated with more frequent exacerbations (*p* = 0.03), higher blood eosinophil levels (*p* = 0.01), and lower lung function (*p* < 0.001) in multi-ancestry urban children cohorts.	Strong, directly validated against severity and exacerbation outcomes	Risk + Severity (validated across severity phenotypes)	[127]

Abbreviations: CAT, catalase; CI, confidence interval; COPD, chronic obstructive pulmonary disease; FEV_1_, forced expiratory volume in 1 s; FVC, forced vital capacity; GSH, reduced glutathione; GSTM1, glutathione S-transferase mu 1; GSTP1, glutathione S-transferase pi 1; GSTT1, glutathione S-transferase theta 1; G × E, gene–environment interaction; HDAC2, histone deacetylase 2; HO-1, heme oxygenase-1; KEAP1, Kelch-like ECH-associated protein 1; MDA, malondialdehyde; MPO, myeloperoxidase; NOS2, nitric oxide synthase 2 gene; NQO1, NAD(P)H:quinone oxidoreductase 1; Nrf2, nuclear factor erythroid 2-related factor 2; OR, odds ratio; SOD2, superoxide dismutase 2 gene.

**Table 5:** Antioxidant enzyme cofactors as prognostic biomarkers in asthma.

Biomarker/Cofactor	Biological Role/Definition	Measurement	Asthma-Associations	Prognostic Value	Sample Size	Country	Refs.
**Se**	Essential cofactor (selenocysteine) in GPx and other selenoenzymes; supports peroxide detoxification	Serum Se ± serum GPx activity (spectrophotometric)	Lower serum Se and GPx activity in pediatric asthma vs. controls	Lower Se/GPx suggests impaired antioxidant capacity; proposed marker of higher oxidative burden and poorer control in panels	32 asthma + 32 controls (children)	Iran (Tehran)	[152]
	Cofactor for GPx (GSH-Px); supports systemic peroxide detoxification	Serum Se + serum GPx (spectrophotometric)	Intrinsic asthma associated with lower serum Se and serum GPx vs. controls	Candidate component of prognostic redox panel; reflects reduced antioxidant defense	46 asthma + 75 controls	Iran (Babol)	[151]
**Zn**	Structural/functional cofactor for Cu/Zn- SOD1; modulates immune balance and antioxidant defense	Serum Zn (photometric) + asthma control (ACT/C-ACT)	Controlled asthma had higher mean serum Zn than uncontrolled; ROC-derived cut-off proposed	Serum Zn showed fair prediction of asthma control (AUROC ~0.72) in pediatric cohort	67 asthmatic children/adolescents	India (AIIMS Rishikesh)	[153]
**Zn + Se + Vitamin D3**	Trace elements support antioxidant enzymes; vitamin D3 is an immunomodulator linked to inflammation/oxidative pathways	ICP-MS (Zn/Se/Cu); vitamin D3 by electrochemiluminescence; control per GINA	Asthmatic children had lower Zn, Se, and vitamin D3 vs. controls; lowest levels in uncontrolled asthma	Low Zn/Se/Vit D3 associated with airway inflammation and poor asthma control; proposed biomarkers for stratification	100 asthma + 75 controls (2019–2021)	India (Lucknow; two-center)	[154]
**Cu**	Cofactor for Cu/Zn-SOD (SOD1); involved in redox enzymes and immune function; deficiency/excess may influence OS	Serum Cu (AAS/ICP-MS depending on study)	Lower serum Cu in adult asthma vs. controls (case–control); lower serum Cu was observed in asthmatic children compared with controls; association with asthma-control categories was not significant	Potential prognostic value when interpreted with Zn/Se and enzyme activity (redox balance)	100 asthma + 170 controls; and 100 asthma + 75 controls	Sudan; India	[155]
**Magnesium (Mg)**	Cofactor for many enzymes; influences airway smooth muscle tone and inflammation (often assessed in trace-element panels)	Serum Mg (AAS/automated analyzers)	No significant difference in Mg in one adult case–control study; serum Mg showed no significant difference between asthmatic patients and controls in both identified studies	Prognostic value inconsistent; may be supportive within broader micronutrient profiling	100 asthma + 170 controls; and 100 asthma + 75 controls	Sudan; India	[155]

Abbreviations: AAS, atomic absorption spectroscopy; ACT, asthma control test; AUROC, area under the receiver operating characteristic curve; C-ACT, childhood asthma control test; Cu, copper; GPx, glutathione peroxidase; GSH-Px, glutathione peroxidase; ICP-MS, inductively coupled plasma mass spectrometry; Mg, magnesium; ROC, receiver operating characteristic; Se, selenium; SOD1, superoxide dismutase 1; Vit D3, vitamin D3; Zn, zinc.

**Table 6:** Oxidative stress damage biomarkers in asthma.

Marker	Definition	Measurement/Method	Asthma Associations	Clinical/Prognostic Value	References	Sample Size	Country
**MDA**	Reactive aldehyde by-product of lipid peroxidation of PUFAs	EBC or plasma/serum; TBARS, HPLC/LC methods	EBC-MDA higher in asthma vs. controls; ICS-treated asthmatics show lower EBC-MDA than untreated	Candidate monitoring marker; responds to ICS in some cohorts	[165]	64 asthma; 14 controls (within *n* = 194 respiratory cohort)	Italy
**MDA**	Reactive aldehyde by-product of lipid peroxidation of PUFAs	Plasma spectrophotometry	Higher in asthma vs. controls; higher in uncontrolled vs. controlled asthma	Predicts poor control (with other redox markers)	[63]	60 asthma; 48 controls	Tunisia
**8-isoprostane**	Stable prostaglandin-like product of lipid peroxidation (in vivo OS marker)	EBC ELISA/MS	Evidence mixed across severe adult cohorts; systematic reviews identify it as one of the most studied EBC markers	Potential severe-asthma breath biomarker, but not consistently correlated with control in all studies	[166,167]	27 severe asthma + 11 healthy + 16 mild	(study setting in Europe)
**OxLDL**	Oxidized LDL particles linked to endothelial/immune activation	Serum immunoassay	Altered systemic OS profile in asthma vs. controls (including OxLDL)	Supports systemic OS phenotyping (esp. pollution/OS studies)	[168]	44 asthma; 37 controls	(study cohort)
**8-OHdG**	Modified nucleoside from oxidative DNA damage (repair/excretion product)	Urine; UHPLC-MS/MS or ELISA	Higher urinary 8-OHdG in asthmatic children vs. healthy; strongly associated with asthma odds in an environmental exposure setting	Useful non-invasive readout of DNA oxidative injury and exposure-linked risk	[169]	252 asthma; 69 controls	China
**Protein carbonyls**	Oxidized proteins forming carbonyl groups	Induced sputum carbonylation assays (protein-level profiling)	Elevated carbonylated sputum proteins in uncontrolled asthma vs. controls; correlated with sputum eosinophilia	Links OS damage to eosinophilic airway inflammation	[170]	23 uncontrolled asthma; 23 controls	Japan
**3-Nitrotyrosine (3-NT)**	Nitration of tyrosine residues (nitrosative stress marker)	EBC LC-MS/MS	~5-fold higher in asthmatic vs. healthy children; not correlated with FeNO or lung function in that cohort	Non-invasive indicator of airway nitrosative events (interpret with clinical context)	[171]	20 asthma; 18 controls	Italy
**AOPP**	Oxidized/cross-linked protein products (often HOCl/MPO-related)	Plasma spectrophotometry/ELISA-type assays	Higher in asthma vs. controls; higher in uncontrolled asthma	Along with MDA, associated with poor control in clinical cohorts	[63]	60 asthma; 48 controls	Tunisia

Abbreviations: MDA, malondialdehyde; PUFA, polyunsaturated fatty acids; EBC, exhaled breath condensate; TBARS, thiobarbituric acid reactive substances; OxLDL, oxidized low-density lipoprotein; LDL, low-density lipoprotein; 8-OHdG, 8-hydroxy-2′-deoxyguanosine; AOPP, advanced oxidation protein products; 3-NT, 3-nitrotyrosine; HPLC, high-performance liquid chromatography; UHPLC-MS/MS, ultra-high-performance liquid chromatography-tandem mass spectrometry; LC-MS/MS, liquid chromatography-tandem mass spectrometry; ELISA, enzyme-linked immunosorbent assay; ICS, inhaled corticosteroids; FeNO, fractional exhaled nitric oxide.

**Table 7:** Circulating/asthma-associated miRNA linked to oxidative stress biology in asthma.

microRNA	Expression in Asthma vs. Controls	Correlated OS Marker	Correlation (R, *p*-Value)	Biological/Pathophysiological Insight	Refs.
**miR-182-5p**	↓ (OVA asthma model; reduced with IL-13 stimulation)	ROS/NOX4-driven OS	Not evaluated (mechanistic)	Directly targets NOX4; restoring miR-182-5p reduces ROS and oxidative-stress-linked epithelial injury/inflammation	[210]
**miR-125b-5p**	↑ (ACO patients; also increased in CSE+OVA models)	Intracellular ROS (flow cytometry ROS-producing cells)	Not evaluated (group differences; siRNA effect)	miR-125b-5p promotes OS via IL6R/TRIAP1 signaling; miR-125b-5p siRNA reduced ROS-producing cells (*p* < 0.05; *p* < 0.001 reported)	[211]
**miR-144**	↑ (OVA-challenged lungs; modulated by antioxidant intervention)	Nrf2-linked antioxidant/OS pathway	Not evaluated	miR-144 is discussed as an Nrf2-regulating miRNA; antioxidant effects in asthma models involve miRNA modulation and Nrf2 restoration	[212]
**miR-34a**	↑ (OVA-challenged lungs; reduced after antioxidant intervention)	Nrf2-linked antioxidant/OS pathway	Not evaluated	miR-34a is reported as an Nrf2-regulating miRNA; changes track with oxidative-stress modulation in allergen models	[212]
**miR-155**	↑ (reported altered with allergen/OS context)	Redox-inflammation crosstalk (OS-responsive miRNA)	Not evaluated	Frequently discussed in ROS/miRNA crosstalk relevant to pulmonary disease, including asthma	[212]
**miR-146a/miR-146b**	↑ (reported altered with allergen/OS context)	Redox-inflammation crosstalk (OS-responsive miRNAs)	Not evaluated	Implicated in inflammatory signaling and OS-linked regulation in pulmonary disease contexts including asthma	[212]
**miR-21**	↑ (reported in asthma models; OS–linked signaling)	OS/pro-inflammatory redox signaling	Not evaluated	Reported to promote OS and inflammation in asthmatic models; also highlighted among asthma-associated miRNAs	[213,214]

Abbreviations: ACO, asthma–COPD overlap; CSE, cigarette smoke extract; IL-13, interleukin-13; IL6R, interleukin-6 receptor; miRNA, microRNA; miR, microRNA; NOX4, NADPH oxidase 4; Nrf2, nuclear factor erythroid 2-related factor 2; OS, oxidative stress; OVA, ovalbumin; ROS, reactive oxygen species; siRNA, small interfering RNA; TRIAP1, TP53-regulated inhibitor of apoptosis 1. Notes: ↑ upregulated; ↓ downregulated; “Not evaluated” indicates the study/review linked the miRNA to OS biology mechanistically or by group comparisons rather than reporting an R value.

**Table 8:** Proposed multilevel redox prognostic panel for asthma severity stratification.

Tier	Category	Exemplary Markers	Biological Role	Clinical Output	Sample/Method	Primary Association
**I, Genetic (Antioxidant)**	Antioxidant enzyme gene variants	*GSTM1/GSTT1* null; *CAT* rs1001179; *SOD2* Val16Ala (rs4880); *GPx1* rs1050450; *HO-1* (GT)n; *NQO1* rs1800566; *Nrf2/KEAP1* variants	Reflect stable, inherited baseline redox detoxification capacity; define predisposition to oxidant-driven injury independent of disease state or treatment [216].	Baseline redox capacity; environmental susceptibility (G × E with pollution); oxidative damage predisposition; steroid-response prediction (Nrf2/KEAP1) [117].	Blood or saliva DNA; PCR-based null genotyping; SNP array or Sanger sequencing	Risk (susceptibility) and/or Severity
**I, Genetic (Pro-oxidant)**	Pro-oxidant enzyme gene variants	*NOS2* polymorphisms; *MPO* −463G>A (rs2333227); *CYBA* C242T (rs4673); *DUOX1/DUOX2* variants; *XDH* polymorphisms	Determine NO production capacity, neutrophilic oxidant potential, and NADPH oxidase-driven superoxide generation. Variants modulate the magnitude of oxidant load in response to inflammatory stimuli [208].	Neutrophilic oxidant load; FeNO production capacity; non-T2 endotype identification; steroid-resistance risk (HDAC2/Nrf2 pathway) [217].	Blood DNA; PCR/SNP array; functional MPO activity assay (serum ELISA)	Risk and/or Severity
**II, Enzymatic Activity (Antioxidant)**	Serum antioxidant enzyme activity	Serum SOD (total, SOD2, EC-SOD/SOD3); CAT activity; GPx activity; PRDX6; PON1 arylesterase activity; Trx/TrxR system	Reflect dynamic, modifiable systemic redox status that fluctuates with disease activity, exacerbation episodes, and therapeutic response. Progressive depletion signals failure of compensatory antioxidant reserve [215].	Severity stratification (mild–moderate–severe); exacerbation risk prediction; antioxidant reserve depletion monitoring; serial trending for treatment response [218].	Serum; spectrophotometric colorimetric assays; ELISA; fluorometric activity assays	Severity (dynamic disease-state marker)
**II, Enzymatic Activity (Pro-oxidant)**	Serum/sputum pro-oxidant enzyme activity	Serum/sputum MPO activity; sputum XO activity; EBC H_2_O_2_; serum NOX/DUOX activity	Index neutrophilic and epithelial oxidant generation. Elevated MPO and XO activity identify non-T2 oxidant-driven inflammation in phenotypes not captured by FeNO or eosinophil counts [219].	Neutrophilic endotype identification; non-T2 phenotype discrimination; exacerbation severity in neutrophilic asthma; complement to FeNO in T2-low patients [216]	Serum, sputum, exhaled breath condensate (EBC); ELISA; colorimetric/fluorometric assays; H_2_O_2_ electrode	Severity (especially neutrophilic/non-T2 phenotype)
**III, Oxidative Damage (Lipid Peroxidation)**	Lipid oxidation end-products	EBC/serum 8-isoprostane (8-iso-PGF2α); serum MDA/TBARS; plasma 4-HNE; oxidized LDL (OxLDL)	Capture cumulative downstream lipid peroxidation injury, the net molecular output of redox imbalance regardless of its enzymatic source. 8-isoprostane is the most standardized non-invasive OS marker in asthma [208].	Cumulative lipid oxidative injury severity; eosinophilic vs. neutrophilic endotype discrimination; pollution-exposure index; severity correlation across phenotypes [117].	EBC, serum, urine; EIA/ELISA (8-isoprostane); HPLC-TBARS (MDA); LC-MS/MS (4-HNE); ELISA (OxLDL)	Severity (cumulative oxidative burden)
**III, Oxidative Damage (DNA & Protein)**	DNA and protein oxidation markers	Urinary 8-oxodG (8-OHdG); plasma protein carbonyls; 3-nitrotyrosine (3-NT); advanced oxidation protein products (AOPP); urinary bromotyrosine	Index genotoxic OS and nitrosative protein modification. Urinary bromotyrosine specifically identifies activated eosinophil peroxidase activity and is a non-invasive biomarker of poor asthma control [216].	Genotoxic and nitrosative stress burden; severity stratification; eosinophil activation index (bromotyrosine); complement to FeNO for T2 endotype quantification [218].	Urine, plasma; ELISA (8-oxodG, AOPP); DNPH assay (protein carbonyls); LC-MS/MS (3-NT, bromotyrosine)	Severity (cumulative genotoxic/nitrosative injury)
**IV, Context (Micronutrient Cofactors)**	Antioxidant enzyme cofactors	Serum Se; Zn; Mg; 25-hydroxyvitamin D [25(OH)D]; plasma vitamins C and E; serum Cu; Cu/Zn ratio	Cofactor availability gates Tier II enzyme activity: Se is essential for GPx catalytic function; Zn and Cu are structural/catalytic components of SOD1; Mg modulates smooth muscle and inflammatory cell redox responses; vitamin D modulates Nrf2-pathway antioxidant gene expression [215].	Modifiable supplementation targets; interpretation context for Tier II depletion (distinguish genetically low capacity vs. cofactor-limited activity); nutritional intervention eligibility [217].	Serum; ICP-MS (Se, Zn, Cu, Mg); immunoassay [25(OH)D]; HPLC (vitamins C, E)	Modifiable context variable (Tier II/III modifier)
**IV, Context (miRNA, Emerging)**	Epigenetic OS regulators	miR-21; miR-155; miR-146a; miR-26a; miR-let-7	Post-transcriptionally regulate expression of Nrf2, SOD2, CAT, GPx, and NF-κB pathway components; modulate steroid sensitivity via HDAC2 regulation; discriminate T2-high from T2-low endotypes via miR-21/miR-146a ratio [219].	Epigenetic OS regulation index; steroid-resistance prediction (HDAC2/Nrf2 axis); phenotype/endotype discrimination (emerging; pre-clinical validation ongoing) [215].	Serum/plasma exosomes or cell-free fraction; qRT-PCR; small RNA-Seq	Severity and steroid-resistance (emerging; requires prospective validation)

Abbreviations: CAT, catalase; CYBA, cytochrome b-245 alpha chain; DUOX, dual oxidase; EBC, exhaled breath condensate; EC-SOD, extracellular superoxide dismutase; EIA, enzyme immunoassay; ELISA, enzyme-linked immunosorbent assay; FeNO, fractional exhaled nitric oxide; GPx1, glutathione peroxidase 1; G × E, gene–environment interaction; HDAC2, histone deacetylase 2; HO-1, heme oxygenase-1; HPLC, high-performance liquid chromatography; LC-MS/MS, liquid chromatography-tandem mass spectrometry; MDA, malondialdehyde; MPO, myeloperoxidase; NQO1, NAD(P)H quinone dehydrogenase 1; Nrf2, nuclear factor erythroid 2-related factor 2; OS, oxidative stress; OxLDL, oxidized low-density lipoprotein; PCR, polymerase chain reaction; PON1, paraoxonase-1; PRDX6, peroxiredoxin 6; qRT-PCR, quantitative reverse-transcription polymerase chain reaction; SNP, single nucleotide polymorphism; SOD, superoxide dismutase; TBARS, thiobarbituric acid reactive substances; Trx, thioredoxin; TrxR, thioredoxin reductase; XO, xanthine oxidase; XDH, xanthine dehydrogenase; 3-NT, 3-nitrotyrosine; 4-HNE, 4-hydroxynonenal; 8-OHdG, 8-hydroxy-2′-deoxyguanosine.

**Table 10:** Obstacles in employing oxidative and antioxidant biomarkers for asthma severity prediction.

Category	Challenge	Impact on Clinical Application	Refs.
**OS Markers**	Variability in assays, limited specificity, and the impact of drugs.	It is challenging to identify accurate biomarkers for diagnosis or disease progression.	[243]
**Gene Polymorphisms**	Small impact sizes, interactions between genes and the environment, and differences between groups	Not particularly helpful in the clinic for forecasting the severity of asthma will get or whether therapy will be effective.	[244]
**Enzyme Cofactors**	The effects of diet and supplements are challenging to figure out because there lack any established investigations.	Makes it challenging to use serum enzyme cofactor levels for controlling asthma.	[245]
**Clinical Translation**	Weak association with clinical outcomes and variability in asthma characteristics.	Difficulties in employing biomarkers for specific or precision medicine.	[246]
**Standardization and Cost**	Various labs obtain outcomes that vary, and expensive tests are not generally available.	Restricts accessibility and uniformity in therapeutic application.	[247]

## Data Availability

Data availability is not applicable to this article, as no new data were created or analyzed in this review.

## References

[1] Liu Y., Zhang M., Wang T., Zhang J. (2025). Reactive Oxygen Species in Asthma: Regulators of Macrophage Polarization and Therapeutic Implications: A Narrative Review. J. Asthma Allergy.

[2] Koumpagioti D., Dimitroglou M., Mpoutopoulou B., Moriki D., Douros K. (2025). The Role of Oxidative Stress in the Pathogenesis of Childhood Asthma: A Comprehensive Review. Children.

[3] Unsal H., Karaguzel D., Sarac B.E., Aytekin E.S., Dal S.T., Gurel D.I., Soyer O., Sekerel B.E., Karaaslan C., Sahiner U.M. (2025). Inflammatory and oxidative stress markers in serum, urine and exhaled breath condensate: Relationship between asthma and obesity in children. Respir. Med..

[4] Peel A.M., Crossman-Barnes C.J., Tang J., Fowler S.J., Davies G.A., Wilson A.M., Loke Y.K. (2017). Biomarkers in adult asthma: A systematic review of 8-isoprostane in exhaled breath condensate. J. Breath Res..

[5] Su X., Ren Y., Li M., Kong L., Kang J. (2020). Association of glutathione S-transferase M1 and T1 genotypes with asthma: A meta-analysis. Medicine.

[6] Marwick J.A., Ito K., Adcock I.M., Kirkham P.A. (2007). Oxidative stress and steroid resistance in asthma and COPD: Pharmacological manipulation of HDAC-2 as a therapeutic strategy. Expert. Opin. Ther. Targets.

[7] Carr T.F., Bleecker E. (2016). Asthma heterogeneity and severity. World Allergy Organ. J..

[8] Hough K.P., Curtiss M.L., Blain T.J., Liu R.M., Trevor J., Deshane J.S., Thannickal V.J. (2020). Airway Remodeling in Asthma. Front. Med..

[9] Durrington H.J., Farrow S.N., Loudon A.S., Ray D.W. (2014). The circadian clock and asthma. Thorax.

[10] Scheer F., Hilton M.F., Evoniuk H.L., Shiels S.A., Malhotra A., Sugarbaker R., Ayers R.T., Israel E., Massaro A.F., Shea S.A. (2021). The endogenous circadian system worsens asthma at night independent of sleep and other daily behavioral or environmental cycles. Proc. Natl. Acad. Sci. USA.

[11] Aleem D.F. (2020). Clinical Profile and pulmonary function tests in Adult Onset Asthma. J. Med. Sci. Clin. Res..

[12] Kuruvilla M.E., Lee F.E., Lee G.B. (2019). Understanding Asthma Phenotypes, Endotypes, and Mechanisms of Disease. Clin. Rev. Allergy Immunol..

[13] Kaur R., Chupp G. (2019). Phenotypes and endotypes of adult asthma: Moving toward precision medicine. J. Allergy Clin. Immunol..

[14] Huang K.Y., Tseng P.T., Wu Y.C., Tu Y.K., Stubbs B., Su K.P., Matsuoka Y.J., Hsu C.W., Lin C.H., Chen Y.W. (2021). Do beta-adrenergic blocking agents increase asthma exacerbation? A network meta-analysis of randomized controlled trials. Sci. Rep..

[15] Morales D.R., Lipworth B.J., Donnan P.T., Jackson C., Guthrie B. (2017). Respiratory effect of beta-blockers in people with asthma and cardiovascular disease: Population-based nested case control study. BMC Med..

[16] Morales D.R., Dreischulte T., Lipworth B.J., Donnan P.T., Jackson C., Guthrie B. (2016). Respiratory effect of beta-blocker eye drops in asthma: Population-based study and meta-analysis of clinical trials. Br. J. Clin. Pharmacol..

[17] Louis R., Satia I., Ojanguren I., Schleich F., Bonini M., Tonia T., Rigau D., Ten Brinke A., Buhl R., Loukides S. (2022). European Respiratory Society guidelines for the diagnosis of asthma in adults. Eur. Respir. J..

[18] Simpson A.J., Drake S., Healy L., Wang R., Bennett M., Wardman H., Durrington H., Fowler S.J., Murray C.S., Simpson A. (2024). Asthma diagnosis: A comparison of established diagnostic guidelines in adults with respiratory symptoms. eClinicalMedicine.

[19] Tuomisto L.E., Ilmarinen P., Lehtimaki L., Tommola M., Kankaanranta H. (2019). Immediate bronchodilator response in FEV(1) as a diagnostic criterion for adult asthma. Eur. Respir. J..

[20] Thiadens H.A., De Bock G.H., Dekker F.W., Huysman J.A., Van Houwelingen J.C., Springer M.P., Postma D.S. (1998). Value of measuring diurnal peak flow variability in the recognition of asthma: A study in general practice. Eur. Respir. J..

[21] Hallstrand T.S., Leuppi J.D., Joos G., Hall G.L., Carlsen K.H., Kaminsky D.A., Coates A.L., Cockcroft D.W., Culver B.H., Diamant Z. (2018). ERS technical standard on bronchial challenge testing: Pathophysiology and methodology of indirect airway challenge testing. Eur. Respir. J..

[22] Kongsupon N., Adab P., Jordan R.E., Huntley C.C., Rattanakanokchai S., Wallbanks S., Li S., Walters G.I. (2025). Screening tools for work-related asthma and their diagnostic accuracy: A systematic review. BMJ Open Respir. Res..

[23] Dweik R.A., Boggs P.B., Erzurum S.C., Irvin C.G., Leigh M.W., Lundberg J.O., Olin A.C., Plummer A.L., Taylor D.R. (2011). An official ATS clinical practice guideline: Interpretation of exhaled nitric oxide levels (FENO) for clinical applications. Am. J. Respir. Crit. Care Med..

[24] Loewenthal L., Menzies-Gow A. (2022). FeNO in Asthma. Semin. Respir. Crit. Care Med..

[25] Chung K.F., Wenzel S.E., Brozek J.L., Bush A., Castro M., Sterk P.J., Adcock I.M., Bateman E.D., Bel E.H., Bleecker E.R. (2014). International ERS/ATS guidelines on definition, evaluation and treatment of severe asthma. Eur. Respir. J..

[26] Bousquet J., Mantzouranis E., Cruz A.A., Ait-Khaled N., Baena-Cagnani C.E., Bleecker E.R., Brightling C.E., Burney P., Bush A., Busse W.W. (2010). Uniform definition of asthma severity, control, and exacerbations: Document presented for the World Health Organization Consultation on Severe Asthma. J. Allergy Clin. Immunol..

[27] Leblanc A., Botelho C., Coimbra A., da Silva J.P., de Castro E.D., Cernadas J.R. (2013). Assessment of asthma control: Clinical, functional and inflammatory aspects. Eur. Ann. Allergy Clin. Immunol..

[28] Khalifa Ahmed E., Abdellah Hamed K.A., Mohamed Ismail D., Ata K.A. (2025). Uncontrolled and severe asthma: Predictive factors and comparison of control assessment tools. Pneumologia.

[29] Miller M.K., Johnson C., Miller D.P., Deniz Y., Bleecker E.R., Wenzel S.E. (2005). Severity assessment in asthma: An evolving concept. J. Allergy Clin. Immunol..

[30] Latorre M., Pistelli R., Carpagnano G.E., Celi A., Puxeddu I., Scichilone N., Spanevello A., Canonica G.W., Paggiaro P. (2023). Symptom versus exacerbation control: An evolution in GINA guidelines?. Ther. Adv. Respir. Dis..

[31] Sies H. (2020). Oxidative Stress: Concept and Some Practical Aspects. Antioxidants.

[32] Vardar Acar N., Ozgul R.K. (2023). The bridge between cell survival and cell death: Reactive oxygen species-mediated cellular stress. EXCLI J..

[33] Birben E., Sahiner U.M., Sackesen C., Erzurum S., Kalayci O. (2012). Oxidative stress and antioxidant defense. World Allergy Organ. J..

[34] Hong Y., Boiti A., Vallone D., Foulkes N.S. (2024). Reactive Oxygen Species Signaling and Oxidative Stress: Transcriptional Regulation and Evolution. Antioxidants.

[35] Kozlov A.V., Javadov S., Sommer N. (2024). Cellular ROS and Antioxidants: Physiological and Pathological Role. Antioxidants.

[36] Jomova K., Alomar S.Y., Alwasel S.H., Nepovimova E., Kuca K., Valko M. (2024). Several lines of antioxidant defense against oxidative stress: Antioxidant enzymes, nanomaterials with multiple enzyme-mimicking activities, and low-molecular-weight antioxidants. Arch. Toxicol..

[37] Bezdicek J., Sekaninova J., Janku M., Makarevic A., Luhova L., Dujickova L., Petrivalsky M. (2025). Reactive oxygen and nitrogen species: Multifaceted regulators of ovarian activitydagger. Biol. Reprod..

[38] Borisov V.B., Forte E. (2022). Bioenergetics and Reactive Nitrogen Species in Bacteria. Int. J. Mol. Sci..

[39] Manoharan R.R., Prasad A., Pospisil P., Kzhyshkowska J. (2024). ROS signaling in innate immunity via oxidative protein modifications. Front. Immunol..

[40] Lennicke C., Cocheme H.M. (2021). Redox metabolism: ROS as specific molecular regulators of cell signaling and function. Mol. Cell..

[41] Sies H., Berndt C., Jones D.P. (2017). Oxidative Stress. Annu. Rev. Biochem..

[42] Lee Y.M., He W., Liou Y.C. (2021). The redox language in neurodegenerative diseases: Oxidative post-translational modifications by hydrogen peroxide. Cell Death Dis..

[43] Forman H.J., Ursini F., Maiorino M. (2014). An overview of mechanisms of redox signaling. J. Mol. Cell. Cardiol..

[44] Denu J.M., Tanner K.G. (1998). Specific and reversible inactivation of protein tyrosine phosphatases by hydrogen peroxide: Evidence for a sulfenic acid intermediate and implications for redox regulation. Biochemistry.

[45] Perez-Quintero L.A., Abidin B.M., Tremblay M.L. (2024). Immunotherapeutic implications of negative regulation by protein tyrosine phosphatases in T cells: The emerging cases of PTP1B and TCPTP. Front. Med..

[46] Andres C.M.C., Perez de la Lastra J.M., Juan C.A., Plou F.J., Perez-Lebena E. (2022). The Role of Reactive Species on Innate Immunity. Vaccines.

[47] Mortimer P.M., Svetitsky S., Thomas D.C. (2025). Chronic granulomatous disease: Lessons in cell biology from monogenic immunodeficiency. Clin. Exp. Immunol..

[48] Mittal M., Siddiqui M.R., Tran K., Reddy S.P., Malik A.B. (2014). Reactive oxygen species in inflammation and tissue injury. Antioxid. Redox Signal..

[49] Yu Y., Liu S., Yang L., Song P., Liu Z., Liu X., Yan X., Dong Q. (2024). Roles of reactive oxygen species in inflammation and cancer. MedComm.

[50] van der Horst D., Carter-Timofte M.E., van Grevenynghe J., Laguette N., Dinkova-Kostova A.T., Olagnier D. (2022). Regulation of innate immunity by Nrf2. Curr. Opin. Immunol..

[51] Pant T., Uche N., Juric M., Zielonka J., Bai X. (2024). Regulation of immunomodulatory networks by Nrf2-activation in immune cells: Redox control and therapeutic potential in inflammatory diseases. Redox Biol..

[52] Harijith A., Ebenezer D.L., Natarajan V. (2014). Reactive oxygen species at the crossroads of inflammasome and inflammation. Front. Physiol..

[53] Abais J.M., Xia M., Zhang Y., Boini K.M., Li P.L. (2015). Redox regulation of NLRP3 inflammasomes: ROS as trigger or effector?. Antioxid. Redox Signal..

[54] Jesenak M., Zelieskova M., Babusikova E. (2017). Oxidative Stress and Bronchial Asthma in Children-Causes or Consequences?. Front. Pediatr..

[55] Barnes P.J. (2020). Oxidative stress-based therapeutics in COPD. Redox Biol..

[56] Dozor A.J. (2010). The role of oxidative stress in the pathogenesis and treatment of asthma. Ann. N. Y. Acad. Sci..

[57] Ismailov I., Kalmatov R., Abdurakhmanov B., Mirza A.M., Chaurasia J.K. (2024). Role of reactive oxygen species in the pathogenesis of bronchial asthma and obstructive pulmonary diseases: Systematic review. Adv. Life Sci..

[58] Penn R.B. (2021). Honing in on the effectors of oxidative stress in the asthmatic lung: Oxidised phosphatidylcholines. Eur. Respir. Soc..

[59] Albano G.D., Gagliardo R.P., Montalbano A.M., Profita M. (2022). Overview of the Mechanisms of Oxidative Stress: Impact in Inflammation of the Airway Diseases. Antioxidants.

[60] Kensler T.W., Wakabayashi N., Biswal S. (2007). Cell survival responses to environmental stresses via the Keap1-Nrf2-ARE pathway. Annu. Rev. Pharmacol. Toxicol..

[61] Ma Q. (2013). Role of nrf2 in oxidative stress and toxicity. Annu. Rev. Pharmacol. Toxicol..

[62] Rahman I., MacNee W. (2000). Oxidative stress and regulation of glutathione in lung inflammation. Eur. Respir. J..

[63] Ammar M., Bahloul N., Amri O., Omri R., Ghozzi H., Kammoun S., Zeghal K., Ben Mahmoud L. (2022). Oxidative stress in patients with asthma and its relation to uncontrolled asthma. J. Clin. Lab. Anal..

[64] Fatani S.H. (2014). Biomarkers of oxidative stress in acute and chronic bronchial asthma. J. Asthma.

[65] Ben Anes A., Ben Nasr H., Fetoui H., Bchir S., Chahdoura H., Yacoub S., Garrouch A., Benzarti M., Tabka Z., Chahed K. (2016). Alteration in systemic markers of oxidative and antioxidative status in Tunisian patients with asthma: Relationships with clinical severity and airflow limitation. J. Asthma.

[66] Nadeem A., Masood A., Siddiqui N. (2008). Oxidant—Antioxidant imbalance in asthma: Scientific evidence, epidemiological data and possible therapeutic options. Ther. Adv. Respir. Dis..

[67] Karadogan B., Beyaz S., Gelincik A., Buyukozturk S., Arda N. (2022). Evaluation of oxidative stress biomarkers and antioxidant parameters in allergic asthma patients with different level of asthma control. J. Asthma.

[68] Sahiner U.M., Birben E., Erzurum S., Sackesen C., Kalayci O. (2011). Oxidative stress in asthma. World Allergy Organ. J..

[69] Powers K.M., Oberley L.W., Domann F.E. (2008). The adventures of superoxide dismutase in health and disease: Superoxide in the balance. Oxidants in Biology: A Question of Balance.

[70] Islam M.N., Rauf A., Fahad F.I., Emran T.B., Mitra S., Olatunde A., Shariati M.A., Rebezov M., Rengasamy K.R.R., Mubarak M.S. (2022). Superoxide dismutase: An updated review on its health benefits and industrial applications. Crit. Rev. Food. Sci. Nutr..

[71] Bowler R.P., Crapo J.D. (2002). Oxidative stress in airways: Is there a role for extracellular superoxide dismutase?. Am. J. Respir. Crit. Care. Med..

[72] Bafana A., Dutt S., Kumar A., Kumar S., Ahuja P.S. (2011). The basic and applied aspects of superoxide dismutase. J. Mol. Catal. B. Enzym..

[73] Alhumaydhi F.A., Younus H., Khan M.A. (2025). Catalase Functions and Glycation: Their Central Roles in Oxidative Stress, Metabolic Disorders, and Neurodegeneration. Catalysts.

[74] Handy D.E., Loscalzo J. (2022). The role of glutathione peroxidase-1 in health and disease. Free Radic. Biol. Med..

[75] Singh S., Verma S.K., Kumar S., Ahmad M.K., Nischal A., Singh S.K., Dixit R.K. (2017). Evaluation of Oxidative Stress and Antioxidant Status in Chronic Obstructive Pulmonary Disease. Scand. J. Immunol..

[76] Andrianjafimasy M., Zerimech F., Akiki Z., Huyvaert H., Le Moual N., Siroux V., Matran R., Dumas O., Nadif R. (2017). Oxidative stress biomarkers and asthma characteristics in adults of the EGEA study. Eur. Respir. J..

[77] Balkrishna A., Solleti S.K., Singh H., Verma S., Sharma N., Nain P., Varshney A. (2020). Herbal decoction Divya-Swasari-Kwath attenuates airway inflammation and remodeling through Nrf-2 mediated antioxidant lung defence in mouse model of allergic asthma. Phytomedicine.

[78] Rhee S.G., Woo H.A., Kil I.S., Bae S.H. (2012). Peroxiredoxin functions as a peroxidase and a regulator and sensor of local peroxides. J. Biol. Chem..

[79] Lu J., Holmgren A. (2014). The thioredoxin antioxidant system. Free Radic. Biol. Med..

[80] Arner E.S., Holmgren A. (2000). Physiological functions of thioredoxin and thioredoxin reductase. Eur. J. Biochem..

[81] Elko E.A., Cunniff B., Seward D.J., Chia S.B., Aboushousha R., van de Wetering C., van der Velden J., Manuel A., Shukla A., Heintz N.H. (2019). Peroxiredoxins and Beyond; Redox Systems Regulating Lung Physiology and Disease. Antioxid. Redox Signal..

[82] Shim H.J., Park S.Y., Kwon H.S., Song W.J., Kim T.B., Moon K.A., Choi J.P., Kim S.J., Cho Y.S. (2020). Oxidative Stress Modulates the Expression Pattern of Peroxiredoxin-6 in Peripheral Blood Mononuclear Cells of Asthmatic Patients and Bronchial Epithelial Cells. Allergy Asthma Immunol. Res..

[83] Inoue K.-i., Takano H., Koike E., Warabi E., Yanagawa T., Yanagisawa R., Ishii T. (2009). Peroxiredoxin I is a negative regulator of Th2-dominant allergic asthma. Int. Immunopharmacol..

[84] Chang S., Linderholm A., Franzi L., Kenyon N., Grasberger H., Harper R. (2013). Dual oxidase regulates neutrophil recruitment in allergic airways. Free Radic. Biol. Med..

[85] Shao M.X., Nadel J.A. (2005). Dual oxidase 1-dependent MUC5AC mucin expression in cultured human airway epithelial cells. Proc. Natl. Acad. Sci. USA.

[86] Ichinose M., Sugiura H., Yamagata S., Koarai A., Tomaki M., Ogawa H., Komaki Y., Barnes P.J., Shirato K., Hattori T. (2003). Xanthine oxidase inhibition reduces reactive nitrogen species production in COPD airways. Eur. Respir. J..

[87] Monteseirin J., Bonilla I., Camacho J., Conde J., Sobrino F. (2001). Elevated secretion of myeloperoxidase by neutrophils from asthmatic patients: The effect of immunotherapy. J. Allergy Clin. Immunol..

[88] Raby B.A. (2019). Asthma severity, nature or nurture: Genetic determinants. Curr. Opin. Pediatr..

[89] Slager R.E., Hawkins G.A., Li X., Postma D.S., Meyers D.A., Bleecker E.R. (2012). Genetics of asthma susceptibility and severity. Clin. Chest Med..

[90] Alhobeira H.A., Mandal R.K., Khan S., Dar S.A., Mahto H., Saeed M., Wahid M., Lohani M., Khan M., Haque S. (2020). Link between MnSOD Ala16Val (rs4880) polymorphism and asthma risk is insignificant from sequential meta-analysis. Bioinformation.

[91] Wang I.J., Karmaus W.J. (2017). Oxidative Stress-Related Genetic Variants May Modify Associations of Phthalate Exposures with Asthma. Int. J. Environ. Res. Public Health.

[92] Babusikova E., Jesenak M., Evinova A., Banovcin P., Dobrota D. (2013). Frequency of polymorphism -262 c/t in catalase gene and oxidative damage in Slovak children with bronchial asthma. Arch. Bronconeumol..

[93] Islam T., McConnell R., Gauderman W.J., Avol E., Peters J.M., Gilliland F.D. (2008). Ozone, oxidant defense genes, and risk of asthma during adolescence. Am. J. Respir. Crit. Care Med..

[94] Wu Z., Chen Q., Lin C., Huang H., Chen L. (2025). Genetic polymorphisms of antioxidant enzymes (GSTP1/CAT/HMOX1/EPHX1) and childhood asthma risk in Fuzhou. Front. Pediatr..

[95] Kansal H., Chopra V., Garg K., Sharma S. (2024). Genetic variations in the antioxidant genes and their role in modulating susceptibility towards chronic obstructive pulmonary disease in the North Indian population. Free Radic. Biol. Med..

[96] Du Y., Zhang H., Xu Y., Ding Y., Chen X., Mei Z., Ding H., Jie Z. (2019). Association among genetic polymorphisms of GSTP1, HO-1, and SOD-3 and chronic obstructive pulmonary disease susceptibility. Int. J. Chron. Obstruct. Pulmon. Dis..

[97] Morin A., Brook J.R., Duchaine C., Laprise C. (2012). Association study of genes associated to asthma in a specific environment, in an asthma familial collection located in a rural area influenced by different industries. Int. J. Environ. Res. Public Health.

[98] Castro-Giner F., Kunzli N., Jacquemin B., Forsberg B., de Cid R., Sunyer J., Jarvis D., Briggs D., Vienneau D., Norback D. (2009). Traffic-related air pollution, oxidative stress genes, and asthma (ECHRS). Environ. Health Perspect..

[99] Lee S.H., Kang M.J., Yu H.S., Hong K., Jung Y.H., Kim H.Y., Seo J.H., Kwon J.W., Kim B.J., Kim H.J. (2014). Association between recent acetaminophen use and asthma: Modification by polymorphism at TLR4. J. Korean Med. Sci..

[100] Polonikov A.V., Ivanov V.P., Bogomazov A.D., Freidin M.B., Illig T., Solodilova M.A. (2014). Antioxidant defense enzyme genes and asthma susceptibility: Gender-specific effects and heterogeneity in gene-gene interactions between pathogenetic variants of the disease. BioMed Res. Int..

[101] Garcia A.M., Allawzi A., Tatman P., Hernandez-Lagunas L., Swain K., Mouradian G., Bowler R., Karimpour-Fard A., Sucharov C.C., Nozik-Grayck E. (2018). R213G polymorphism in SOD3 protects against bleomycin-induced inflammation and attenuates induction of proinflammatory pathways. Physiol. Genom..

[102] Jablonska E., Gromadzinska J., Peplonska B., Fendler W., Reszka E., Krol M.B., Wieczorek E., Bukowska A., Gresner P., Galicki M. (2015). Lipid peroxidation and glutathione peroxidase activity relationship in breast cancer depends on functional polymorphism of GPX1. BMC Cancer.

[103] Gao P.S., Kawada H., Kasamatsu T., Mao X.Q., Roberts M.H., Miyamoto Y., Yoshimura M., Saitoh Y., Yasue H., Nakao K. (2000). Variants of NOS1, NOS2, and NOS3 genes in asthmatics. Biochem. Biophys. Res. Commun..

[104] Grasemann H., Yandava C., Drazen J. (1999). Neuronal NO synthase (NOS1) is a major candidate gene for asthma. Clin. Exp. Allergy Suppl..

[105] Polonikov A.V., Solodilova M.A., Ivanov V.P. (2009). Genetic variation of myeloperoxidase gene contributes to atopic asthma susceptibility: A preliminary association study in Russian population. J. Asthma.

[106] Nowakowska J., Sobkowiak P., Bręborowicz A., Mrówczyńska M., Wojsyk-Banaszak I., Szczepankiewicz A. (2021). Nitric oxide synthase 2 promoter polymorphism is a risk factor for allergic asthma in children. Medicina.

[107] Castro M.C., Matos A., Ferreira J., Prabhudas R., Bicho M. (2014). Inducible nitric oxide synthase polymorphism in asthmatic patients. Eur. Respir. J..

[108] Batra J., Pratap Singh T., Mabalirajan U., Sinha A., Prasad R., Ghosh B. (2007). Association of inducible nitric oxide synthase with asthma severity, total serum immunoglobulin E and blood eosinophil levels. Thorax.

[109] Leung T.F., Liu E.K., Tang N.L., Ko F.W., Li C.Y., Lam C.W., Wong G.W. (2005). Nitric oxide synthase polymorphisms and asthma phenotypes in Chinese children. Clin. Exp. Allergy.

[110] Fan Z., Liu T., Na W. (2023). Association of nitric oxide synthase gene polymorphism with asthma: A systematic review and meta-analysis. Clin. Respir. J..

[111] Holla L.I., Buckova D., Kuhrova V., Stejskalova A., Francova H., Znojil V., Vacha J. (2002). Prevalence of endothelial nitric oxide synthase gene polymorphisms in patients with atopic asthma. Clin. Exp. Allergy.

[112] Castro M.C., Matos A., Ferreira J., Prabhudas R., Bicho M. (2014). Asthma and myeloperoxidase gene promoter region polymorphism. Eur. Respir. J..

[113] Ivanov V.P., Solodilova M.A., Polonikov A.V., Khoroshaia I.V., Kozhukhov M.A., Panfilov V.I. (2008). Association of C242T and A640G polymorphisms in the gene for p22phox subunit of NADPH oxidase with the risk of bronchial asthma: A pilot study. Genetika.

[114] Izakovičová Hollá L., Kaňková K., Jurajda M., Znojil V. (2007). Haplotype analysis of NADPH oxidase p22phox gene in Czech patients with bronchial asthma. Int. Arch. Allergy Immunol..

[115] Gao L., Rafaels N., Dudenkov T.M., Damarla M., Damico R., Maloney J.P., Moss M., Martin G.S., Sevransky J., Shanholtz C. (2023). Xanthine oxidoreductase gene polymorphisms are associated with high risk of sepsis and organ failure. Respir. Res..

[116] Hussain M.S., Jahan N., Or Rashid M.M., Hossain M.S., Chen U., Rahman N. (2019). Antihyperlipidemic screening and plasma uric acid reducing potential of Momordica charantia seeds on Swiss albino mice model. Heliyon.

[117] Ercan H., Birben E., Dizdar E.A., Keskin O., Karaaslan C., Soyer O.U., Dut R., Sackesen C., Besler T., Kalayci O. (2006). Oxidative stress and genetic and epidemiologic determinants of oxidant injury in childhood asthma. J. Allergy Clin. Immunol..

[118] Palmer C.N., Doney A.S., Lee S.P., Murrie I., Ismail T., Macgregor D.F., Mukhopadhyay S. (2006). Glutathione S-transferase M1 and P1 genotype, passive smoking, and peak expiratory flow in asthma. Pediatrics.

[119] Cunningham J., Cheek E., Tavendale R., Mitra A., MacGregor D., Palmer C., Smith H., Mukhopadhyay S., Basu K. (2012). Genetic variants implicated in airway remodelling are risk factors for asthma severity in children and young adults. Arch. Dis. Child..

[120] Turner S., Francis B., Wani N., Vijverberg S., Pino-Yanes M., Mukhopadhyay S., Tavendale R., Palmer C., Burchard E.G., Merid S.K. (2018). Variants in genes coding for glutathione S-transferases and asthma outcomes in children. Pharmacogenomics.

[121] Majid W.N., Mehdi L.M. (2021). Correlation between Superoxide Dismutase 1 and 2 Polymorphisms in Asthma Patients. Indian J. Forensic. Med. Toxicol..

[122] Mak J.C., Leung H.C., Ho S.P., Ko F.W., Cheung A.H., Ip M.S., Chan-Yeung M.M. (2006). Polymorphisms in manganese superoxide dismutase and catalase genes: Functional study in Hong Kong Chinese asthma patients. Clin. Exp. Allergy.

[123] Hirai K., Shirai T., Suzuki M., Shimomura T., Itoh K. (2018). Association between (CCTTT)n repeat polymorphism in NOS2 promoter and asthma exacerbations. J. Allergy Clin. Immunol..

[124] Tizaoui K., Hamzaoui K., Hamzaoui A. (2017). Update on Asthma Genetics: Results From Meta-Analyses of Candidate Gene Association Studies. Curr. Mol. Med..

[125] Zayed H. (2020). Novel Comprehensive Bioinformatics Approaches to Determine the Molecular Genetic Susceptibility Profile of Moderate and Severe Asthma. Int. J. Mol. Sci..

[126] Slager R.E., Li X., Meyers D.A., Bleecker E.R., Bernstein J.A., Levy M.L. (2014). Genetics and Asthma. Clinical Asthma: Theory and Practice.

[127] Dapas M., Wentworth-Sheilds W., Thompson E.E., Kumar R., Lippner E., Wood R.A., O’Connor G.T., Khurana Hershey G.K., Gruchalla R.S., Liu A.H. (2026). Cumulative Genetic Risk for Asthma Contributes to Disease Severity in Children with Asthma living in Urban Environments. J. Allergy Clin. Immunol. Glob..

[128] Bassu S., Mangoni A.A., Argiolas D., Carru C., Pirina P., Fois A.G., Zinellu A. (2023). A systematic review and meta-analysis of paraoxonase-1 activity in asthma. Clin. Exp. Med..

[129] Camps J., Iftimie S., Arenas M., Castañé H., Jiménez-Franco A., Castro A., Joven J. (2023). Paraoxonase-1: How a xenobiotic detoxifying enzyme has become an actor in the pathophysiology of infectious diseases and cancer. Chem. Biol. Interact..

[130] Durrington P.N., Bashir B., Soran H. (2023). Paraoxonase 1 and atherosclerosis. Front. Cardiovasc. Med..

[131] Litvinov D., Mahini H., Garelnabi M. (2012). Antioxidant and anti-inflammatory role of paraoxonase 1: Implication in arteriosclerosis diseases. N. Am. J. Med. Sci..

[132] Jung J.H., Kang S.A., Park J.-H., Kim S.-D., Yu H.S., Mun S.J., Cho K.-S. (2024). Paraoxonase-1 is a pivotal regulator responsible for suppressing allergic airway inflammation through adipose stem cell-derived extracellular vesicles. Int. J. Mol. Sci..

[133] Anber N.H., Ahmed Shahin H.E., Badawy H.K., Oraby E.A., Mohammed S.A., Shaaban E.I.A., Attia Z.R., Mohamed S., Shabana M.F., El-Eshmawy M.A. (2025). Potential Impact of SOD2 (rs4880; p.Val16Ala) Variant with the Susceptibility for Childhood Bronchial Asthma. Biochem. Genet..

[134] Gill A.J., Garza R., Ambegaokar S.S., Gelman B.B., Kolson D.L. (2018). Heme oxygenase-1 promoter region (GT)n polymorphism associates with increased neuroimmune activation and risk for encephalitis in HIV infection. J. Neuroinflamm..

[135] Basharat Z., Messaoudi A., Ruba S., Yasmin A. (2016). NQO1 rs1800566 polymorph is more prone to NOx induced lung injury: Endorsing deleterious functionality through informatics approach. Gene.

[136] Ungvári I., Hadadi É., Virág V., Nagy A., Kiss A., Kalmár Á., Zsigmond G., Semsei Á.F., Falus A., Szalai C. (2012). Relationship between air pollution, NFE2L2 gene polymorphisms and childhood asthma in a Hungarian population. J. Community Genet..

[137] Bouzigon E., Monier F., Boussaha M., Le Moual N., Huyvaert H., Matran R., Letort S., Bousquet J., Pin I., Lathrop M. (2012). Associations between nitric oxide synthase genes and exhaled NO-related phenotypes according to asthma status. PLoS ONE.

[138] Martinez B., Barrios K., Vergara C., Mercado D., Jimenez S., Gusmao L., Caraballo L. (2007). A NOS1 gene polymorphism associated with asthma and specific immunoglobulin E response to mite allergens in a Colombian population. Int. Arch. Allergy Immunol..

[139] Lin W., Chen H., Chen X., Guo C. (2024). The Roles of Neutrophil-Derived Myeloperoxidase (MPO) in Diseases: The New Progress. Antioxidants.

[140] Abdullah S.O., Ramadan G.M., Makki Al-Hindy H.A., Mousa M.J., Al-Mumin A., Jihad S., Hafidh S., Kadhum Y. (2022). Serum Myeloperoxidase as a Biomarker of Asthma Severity Among Adults: A Case Control Study. Rep. Biochem. Mol. Biol..

[141] Kim M.J., Kim S.Y., Kim J.D., Park M., Kim Y.H., Kim K.W., Sohn M.H. (2024). Release of sputum neutrophil granules is associated with pulmonary function and disease severity in childhood asthma. BMC Pulm. Med..

[142] Takacova T., Schirmer M.A. (2025). Impact of genetic variability on NADPH oxidase activity: An extensive genotype-phenotype assessment. Free. Radic. Res..

[143] Fischer H. (2009). Mechanisms and function of DUOX in epithelia of the lung. Antioxid. Redox Signal..

[144] Bedard K., Krause K.H. (2007). The NOX family of ROS-generating NADPH oxidases: Physiology and pathophysiology. Physiol. Rev..

[145] Bortolotti M., Polito L., Battelli M.G., Bolognesi A. (2021). Xanthine oxidoreductase: One enzyme for multiple physiological tasks. Redox Biol..

[146] Boueiz A., Damarla M., Hassoun P.M. (2008). Xanthine oxidoreductase in respiratory and cardiovascular disorders. Am. J. Physiol. Lung. Cell. Mol. Physiol..

[147] Sugiura H., Komaki Y., Koarai A., Ichinose M. (2008). Nitrative stress in refractory asthma. J. Allergy Clin. Immunol..

[148] Huff R.D., Hsu A.C., Nichol K.S., Jones B., Knight D.A., Wark P.A.B., Hansbro P.M., Hirota J.A. (2017). Regulation of xanthine dehydrogensase gene expression and uric acid production in human airway epithelial cells. PLoS ONE.

[149] Kudo M., Moteki T., Sasaki T., Konno Y., Ujiie S., Onose A., Mizugaki M., Ishikawa M., Hiratsuka M. (2008). Functional characterization of human xanthine oxidase allelic variants. Pharmacogenet. Genom..

[150] Kudo M., Sasaki T., Ishikawa M., Hirasawa N., Hiratsuka M. (2010). Functional characterization of genetic polymorphisms identified in the promoter region of the xanthine oxidase gene. Drug Metab. Pharmacokinet..

[151] Qujeq D., Hidari B., Bijani K., Shirdel H. (2003). Glutathione peroxidase activity and serum selenium concentration in intrinsic asthmatic patients. Clin. Chem. Lab. Med..

[152] Tabatabaei A., Babaee M., Moradi N., Nabavi M., Arshi S., Fallah S. (2020). Serum Concentration of Selenium and GPX Enzyme Activity in Iranian Children with Asthma. Mod. Care. J..

[153] Rajkumar S., Bhat N.K., Kumar V., Bolia R., Verma P.K., Kumar M., Chacham S., Mirza A.A. (2023). Association of serum zinc levels and symptom control of asthma in children and adolescents—A prospective observational study. Eur. J. Pediatr..

[154] Srivastava S., Tiwari V., Singh S., Karoli R., Bhattacharya P., Gupta N. (2023). Low Serum Levels of Zinc, Selenium, and Vitamin D3 Are Biomarkers of Airway Inflammation and Poor Asthma Control: A Two-Centre Study. Cureus.

[155] Hussein M.M., Yousif A.A., Saeed A.M. (2008). Serum Levels of Selenium, Zinc, Copper and Magnesium in Asthmatic Patients: A Case Control Study. Sudan J. Med. Sci..

[156] Jiang H., Yang G., Chen J., Yuan S., Wu J., Zhang J., Zhang L., Yuan J., Lin J., Chen J. (2024). The correlation between selenium intake and lung function in asthmatic people: A cross-sectional study. Front. Nutr..

[157] Dinardo G., Indolfi C., Klain A., Grella C., Tosca M.A., Ruocco E., Miraglia Del Giudice M., Ciprandi G. (2025). The Role of Zinc in Pediatric Asthma and Allergic Rhinitis: Mechanisms and Clinical Implications. Nutrients.

[158] Williamson A., Martineau A.R., Sheikh A., Jolliffe D., Griffiths C.J. (2023). Vitamin D for the management of asthma. Cochrane Database Syst. Rev..

[159] Hemila H. (2013). Vitamin C may alleviate exercise-induced bronchoconstriction: A meta-analysis. BMJ Open.

[160] Rovsing A.H., Savran O., Ulrik C.S. (2023). Magnesium sulfate treatment for acute severe asthma in adults-a systematic review and meta-analysis. Front. Allergy.

[161] Yang M., Li Y., Yao C., Wang Y., Yan C. (2023). Association between serum copper-zinc ratio and respiratory tract infection in children and adolescents. PLoS ONE.

[162] Lv N., Xiao L., Ma J. (2014). Dietary pattern and asthma: A systematic review and meta-analysis. J. Asthma Allergy.

[163] Melinte O.E., Stavarache E.I., Dobrin M.E., Cernomaz A.T., Cioroiu I.B., Popa D.R., Grosu-Creanga I.A., Zabara Antal A., Trofor A.C. (2025). Oxidative Stress and Risk Factors in Adult Patients with Bronchial Asthma: A Clinical Analysis of Representative Biomarkers. J. Clin. Med..

[164] Bazan-Socha S., Wojcik K., Olchawa M., Sarna T., Pieta J., Jakiela B., Soja J., Okon K., Zarychta J., Zareba L. (2022). Increased Oxidative Stress in Asthma-Relation to Inflammatory Blood and Lung Biomarkers and Airway Remodeling Indices. Biomedicines.

[165] Bartoli M.L., Novelli F., Costa F., Malagrino L., Melosini L., Bacci E., Cianchetti S., Dente F.L., Di Franco A., Vagaggini B. (2011). Malondialdehyde in exhaled breath condensate as a marker of oxidative stress in different pulmonary diseases. Mediat. Inflamm..

[166] Piotrowski W.J., Majewski S., Marczak J., Kurmanowska Z., Gorski P., Antczak A. (2012). Exhaled breath 8-isoprostane as a marker of asthma severity. Arch. Med. Sci..

[167] Gagliani C., Brinkman P., Del Riccio M., Benfante A., Shahbazi Khamas S., Maitland-van der Zee A.H., Principe S., Scichilone N. (2025). Advances in exhaled breath condensate markers for severe asthma management: A systematic review. Expert Rev. Respir. Med..

[168] Santibanez M., Nunez-Robainas A., Barreiro E., Exposito A., Aguero J., Garcia-Rivero J.L., Abascal B., Amado C.A., Ruiz-Cubillan J.J., Fernandez-Sobaler C. (2025). Characterization of Systemic Oxidative Stress in Asthmatic Adults Compared to Healthy Controls and Its Association with the Oxidative Potential of Particulate Matter Collected Using Personal Samplers. Antioxidants.

[169] Kuang H., Li Z., Lv X., Wu P., Tan J., Wu Q., Li Y., Jiang W., Pang Q., Wang Y. (2021). Exposure to volatile organic compounds may be associated with oxidative DNA damage-mediated childhood asthma. Ecotoxicol. Environ. Saf..

[170] Nagai K., Betsuyaku T., Konno S., Ito Y., Nasuhara Y., Hizawa N., Kondo T., Nishimura M. (2008). Diversity of protein carbonylation in allergic airway inflammation. Free Radic. Res..

[171] Baraldi E., Giordano G., Pasquale M.F., Carraro S., Mardegan A., Bonetto G., Bastardo C., Zacchello F., Zanconato S. (2006). 3-Nitrotyrosine, a marker of nitrosative stress, is increased in breath condensate of allergic asthmatic children. Allergy.

[172] Tsikas D. (2017). Assessment of lipid peroxidation by measuring malondialdehyde (MDA) and relatives in biological samples: Analytical and biological challenges. Anal. Biochem..

[173] Gegotek A., Skrzydlewska E. (2019). Biological effect of protein modifications by lipid peroxidation products. Chem. Phys. Lipids.

[174] Sonowal H., Ramana K.V. (2019). 4-Hydroxy-Trans-2-Nonenal in the Regulation of Anti-Oxidative and Pro-Inflammatory Signaling Pathways. Oxid. Med. Cell. Longev..

[175] Wang D., Cui Y., Gao F., Zheng W., Li J., Xian Z. (2023). Effects of imperatorin on airway remodeling in bronchial asthma through S1PR2/STAT3 signaling pathway. Cell. Mol. Biol..

[176] Tang W., Dong M., Teng F., Cui J., Zhu X., Wang W., Wuniqiemu T., Qin J., Yi L., Wang S. (2021). Environmental allergens house dust mite-induced asthma is associated with ferroptosis in the lungs. Exp. Ther. Med..

[177] Worgall S. (2023). Environmental exposures: Another effect on sphingolipids in asthma?. Thorax.

[178] Cordiano R., Di Gioacchino M., Mangifesta R., Panzera C., Gangemi S., Minciullo P.L. (2023). Malondialdehyde as a Potential Oxidative Stress Marker for Allergy-Oriented Diseases: An Update. Molecules.

[179] Li Y., Zhao T., Li J., Xia M., Li Y., Wang X., Liu C., Zheng T., Chen R., Kan D. (2022). Oxidative Stress and 4-hydroxy-2-nonenal (4-HNE): Implications in the Pathogenesis and Treatment of Aging-related Diseases. J. Immunol. Res..

[180] Shoeb M., Ansari N.H., Srivastava S.K., Ramana K.V. (2014). 4-Hydroxynonenal in the pathogenesis and progression of human diseases. Curr. Med. Chem..

[181] Michaeloudes C., Abubakar-Waziri H., Lakhdar R., Raby K., Dixey P., Adcock I.M., Mumby S., Bhavsar P.K., Chung K.F. (2022). Molecular mechanisms of oxidative stress in asthma. Mol. Asp. Med..

[182] Ioannidis M., Tjepkema J., Uitbeijerse M.R.P., van den Bogaart G. (2025). Immunomodulatory effects of 4-hydroxynonenal. Redox Biol..

[183] Nandedkar S.D., Weihrauch D., Xu H., Shi Y., Feroah T., Hutchins W., Rickaby D.A., Duzgunes N., Hillery C.A., Konduri K.S. (2011). D-4F, an apoA-1 mimetic, decreases airway hyperresponsiveness, inflammation, and oxidative stress in a murine model of asthma. J. Lipid Res..

[184] Jaganjac M., Milkovic L., Gegotek A., Cindric M., Zarkovic K., Skrzydlewska E., Zarkovic N. (2020). The relevance of pathophysiological alterations in redox signaling of 4-hydroxynonenal for pharmacological therapies of major stress-associated diseases. Free Radic. Biol. Med..

[185] Voynow J.A., Kummarapurugu A. (2011). Isoprostanes and asthma. Biochim. Biophys. Acta.

[186] Woo S.-D., Park H.S., Yang E.-M., Ban G.-Y., Park H.-S. (2024). 8-Iso-prostaglandin F2α as a biomarker of type 2 low airway inflammation and remodeling in adult asthma. Ann. Allergy Asthma Immunol..

[187] Roberts L.J., Fessel J.P., Davies S.S. (2005). The biochemistry of the isoprostane, neuroprostane, and isofuran Pathways of lipid peroxidation. Brain Pathol..

[188] Wood L.G., Gibson P.G., Garg M.L. (2003). Biomarkers of lipid peroxidation, airway inflammation and asthma. Eur. Respir. J..

[189] Zanjani B.N., Samadi A., Isikhan S.Y., Lay I., Beyaz S., Gelincik A., Buyukozturk S., Arda N. (2023). Plasma levels of oxysterols 7-ketocholesterol and cholestane-3beta, 5alpha, 6beta-triol in patients with allergic asthma. J. Asthma.

[190] Vasconcelos L.H.C., Ferreira S.R.D., Silva M., Ferreira P.B., de Souza I.L.L., Cavalcante F.A., da Silva B.A. (2021). Uncovering the Role of Oxidative Imbalance in the Development and Progression of Bronchial Asthma. Oxid. Med. Cell. Longev..

[191] Li M., Li M., Hou Y., He H., Jiang R., Wang C., Sun S. (2023). Ferroptosis triggers airway inflammation in asthma. Ther. Adv. Respir. Dis..

[192] Pirogov A.B., Prikhodko A.G., Perelman J.M., Borodin E.A., Naumov D.E., Zinovyev S.V., Shtarberg M.A. (2016). Peculiarities of Airway Inflammation and Lipid Peroxidation in the Development of Hyperosmotic Airway Hyperresponsiveness in Patients with Asthma. Int. J. Biomed.

[193] Diaz-Garcia E., Sanz-Rubio D., Garcia-Tovar S., Alfaro E., Cubero P., Gil A.V., Marin J.M., Cubillos-Zapata C., Garcia-Rio F. (2023). Inflammasome activation mediated by oxidised low-density lipoprotein in patients with sleep apnoea and early subclinical atherosclerosis. Eur. Respir. J..

[194] Deng K., Wang J., Oliver B.G., Wood L.G., Wang G. (2025). Dyslipidemia in asthma: Treatable trait, or just a common comorbidity?. Chin. Med. J..

[195] Chen Y., Yang M., Huang W., Chen W., Zhao Y., Schulte M.L., Volberding P., Gerbec Z., Zimmermann M.T., Zeighami A. (2019). Mitochondrial Metabolic Reprogramming by CD36 Signaling Drives Macrophage Inflammatory Responses. Circ. Res..

[196] Liu L., Liu Y., Zhang X., Yuan Y.L., Chen Z.H., Chen-Yu Hsu A., Oliver B.G., Xie M., Qin L., Li W.M. (2023). Dyslipidemia Is Associated with Worse Asthma Clinical Outcomes: A Prospective Cohort Study. J. Allergy Clin. Immunol. Pract..

[197] Chen P.Y., Chen C.W., Su Y.J., Chang W.H., Kao W.F., Yang C.C., Wang I.J. (2020). Associations between Levels of Urinary Oxidative Stress of 8-OHdG and Risk of Atopic Diseases in Children. Int. J. Environ. Res. Public Health.

[198] Chaiwong W., Liwsrisakun C., Inchai J., Duangjit P., Bumroongkit C., Deesomchok A., Theerakittikul T., Limsukon A., Tajarernmuang P., Niyatiwatchanchai N. (2025). Biomarkers of Oxidative Stress, Systemic Inflammation and Thrombosis in Adult Asthmatic Patients Treated with Inhaled Corticosteroids During Exposure to Fine Particulate Matter. J. Clin. Med..

[199] Urbaniak S.K., Boguszewska K., Szewczuk M., Kaźmierczak-Barańska J., Karwowski B.T. (2020). 8-Oxo-7, 8-dihydro-2′-deoxyguanosine (8-oxodG) and 8-hydroxy-2′-deoxyguanosine (8-OHdG) as a potential biomarker for gestational diabetes mellitus (GDM) development. Molecules.

[200] Belanger K.K., Ameredes B.T., Boldogh I., Aguilera-Aguirre L. (2016). The Potential Role of 8-Oxoguanine DNA Glycosylase-Driven DNA Base Excision Repair in Exercise-Induced Asthma. Mediat. Inflamm..

[201] Ba X., Aguilera-Aguirre L., Sur S., Boldogh I. (2015). 8-Oxoguanine DNA glycosylase-1-driven DNA base excision repair: Role in asthma pathogenesis. Curr. Opin. Allergy Clin. Immunol..

[202] Cai X., Xu Q., Yin T., Li X., Cheng Y., Li X., Hao C. (2025). Modulation of SUMO1-TOP1 DNA damage repair by TOPORS following ovalbumin-induced oxidative stress in macrophages. Toxicol. Lett..

[203] Foreman R.C., Mercer P.F., Kroegel C., Warner J.A. (1999). Role of the eosinophil in protein oxidation in asthma: Possible effects on proteinase/antiproteinase balance. Int. Arch. Allergy Immunol..

[204] Wang Z., DiDonato J.A., Buffa J., Comhair S.A., Aronica M.A., Dweik R.A., Lee N.A., Lee J.J., Thomassen M.J., Kavuru M. (2016). Eosinophil Peroxidase Catalyzed Protein Carbamylation Participates in Asthma. J. Biol. Chem..

[205] Hanazawa T., Kharitonov S.A., Barnes P.J. (2000). Increased nitrotyrosine in exhaled breath condensate of patients with asthma. Am. J. Respir. Crit. Care Med..

[206] Kaminsky D.A., Mitchell J., Carroll N., James A., Soultanakis R., Janssen Y. (1999). Nitrotyrosine formation in the airways and lung parenchyma of patients with asthma. J. Allergy Clin. Immunol..

[207] Ghosh S., Janocha A.J., Aronica M.A., Swaidani S., Comhair S.A., Xu W., Zheng L., Kaveti S., Kinter M., Hazen S.L. (2006). Nitrotyrosine proteome survey in asthma identifies oxidative mechanism of catalase inactivation. J. Immunol..

[208] Comhair S.A., Erzurum S.C. (2010). Redox control of asthma: Molecular mechanisms and therapeutic opportunities. Antioxid. Redox Signal..

[209] Babanov S.A., Strizhakov L.A., Baikova A.G., Budash D.S., Agarkova A.S., Vostroknutova M.Y. (2021). Clinical and immunological features and prognosis of different phenotypes of occupational asthma. Russ. J. Occup. Health Ind. Ecol..

[210] Wang Z., Song Y., Jiang J., Piao Y., Li L., Bai Q., Xu C., Liu H., Li L., Piao H. (2022). MicroRNA-182-5p Attenuates Asthmatic Airway Inflammation by Targeting NOX4. Front. Immunol..

[211] Chang Y.P., Tsai Y.H., Chen Y.M., Huang K.T., Lee C.P., Hsu P.Y., Chen H.C., Lin M.C., Chen Y.C. (2024). Upregulated microRNA-125b-5p in patients with asthma-COPD overlap mediates oxidative stress and late apoptosis via targeting IL6R/TRIAP1 signaling. Respir. Res..

[212] Climent M., Viggiani G., Chen Y.W., Coulis G., Castaldi A. (2020). MicroRNA and ROS Crosstalk in Cardiac and Pulmonary Diseases. Int. J. Mol. Sci..

[213] Sharma R., Tiwari A., McGeachie M.J. (2022). Recent miRNA Research in Asthma. Curr. Allergy Asthma Rep..

[214] Gil-Martinez M., Lorente-Sorolla C., Naharro S., Rodrigo-Munoz J.M., Del Pozo V. (2023). Advances and Highlights of miRNAs in Asthma: Biomarkers for Diagnosis and Treatment. Int. J. Mol. Sci..

[215] Kunc P., Fabry J., Lucanska M., Pecova R. (2020). Biomarkers of Bronchial Asthma. Physiol. Res..

[216] Erzurum S.C. (2016). New Insights in Oxidant Biology in Asthma. Ann. Am. Thorac. Soc..

[217] Popovic-Grle S., Stajduhar A., Lampalo M., Rnjak D. (2021). Biomarkers in Different Asthma Phenotypes. Genes.

[218] Mao R., Jiang Z., Min Z., Wang G., Xie M., Gao P., Zhu L., Li H., Chen Z. (2023). Peripheral neutrophils and oxidative stress-associated molecules for predicting the severity of asthma: A cross-sectional study based on multidimensional assessment. Front. Med..

[219] Bayes H.K., Cowan D.C. (2016). Biomarkers and asthma management: An update. Curr. Opin. Allergy Clin. Immunol..

[220] Cho H.Y., Marzec J., Kleeberger S.R. (2015). Functional polymorphisms in Nrf2: Implications for human disease. Free Radic. Biol. Med..

[221] Audousset C., McGovern T., Martin J.G. (2021). Role of Nrf2 in Disease: Novel Molecular Mechanisms and Therapeutic Approaches—Pulmonary Disease/Asthma. Front. Physiol..

[222] Frijhoff J., Winyard P.G., Zarkovic N., Davies S.S., Stocker R., Cheng D., Knight A.R., Taylor E.L., Oettrich J., Ruskovska T. (2015). Clinical Relevance of Biomarkers of Oxidative Stress. Antioxid. Redox Signal..

[223] Marrocco I., Altieri F., Peluso I. (2017). Measurement and Clinical Significance of Biomarkers of Oxidative Stress in Humans. Oxid. Med. Cell. Longev..

[224] Rupani H., Kent B.D. (2022). Using Fractional Exhaled Nitric Oxide Measurement in Clinical Asthma Management. Chest.

[225] Couillard S., Pavord I.D., Heaney L.G., Petousi N., Hinks T.S.C. (2022). Sub-stratification of type-2 high airway disease for therapeutic decision-making: A ‘bomb’ (blood eosinophils) meets ‘magnet’ (FeNO) framework. Respirology.

[226] Katsoulis K., Kontakiotis T., Gerou S., Kougioulis M., Lithoxopoulou H., Papakosta D. (2010). Alterations of erythrocyte superoxide dismutase activity in patients suffering from asthma attacks. Monaldi. Arch. Chest Dis..

[227] Cialkowska-Rysz A., Kowalczyk M., Gottwald L., Kazmierczak-Lukaszewicz S. (2012). The comparison of common cancer types and the coincidence of concomitant chronic diseases between palliative home care patients in Lodz Voivodeship and the general Polish population. Arch. Med. Sci..

[228] Samitas K., Chorianopoulos D., Vittorakis S., Zervas E., Economidou E., Papatheodorou G., Loukides S., Gaga M. (2009). Exhaled cysteinyl-leukotrienes and 8-isoprostane in patients with asthma and their relation to clinical severity. Respir. Med..

[229] Robinson D.S., Campbell D.A., Durham S.R., Pfeffer J., Barnes P.J., Chung K.F. (2003). Asthma and Allergy Research Group of the National Heart and Lung Institute. Systematic assessment of difficult-to-treat asthma. Eur. Respir. J..

[230] Valavanidis A., Vlachogianni T., Fiotakis C. (2009). 8-hydroxy-2′-deoxyguanosine (8-OHdG): A critical biomarker of oxidative stress and carcinogenesis. J. Environ. Sci. Health Part C Environ. Carcinog. Ecotoxicol. Rev..

[231] Comhair S., Khan A., Erzurum S. (2011). Superoxide dismutase as a longitudinal biomarker of lung function in asthma. Eur. Respir. J..

[232] Abboud M.M., Al-Rawashde F.A., Al-Zayadneh E.M. (2022). Alterations of serum and saliva oxidative markers in patients with bronchial asthma. J. Asthma.

[233] Al-Kinani M.F.H., Sayyah S.G. (2016). Evaluate of antioxidant enzymes superoxide dismutase, glutathione peroxidase and catalase levels in asthma patients. J. Nat. Sci. Res..

[234] Kabesch M., Hoefler C., Carr D., Leupold W., Weiland S.K., von Mutius E. (2004). Glutathione S transferase deficiency and passive smoking increase childhood asthma. Thorax.

[235] Turner S., Francis B., Vijverberg S., Pino-Yanes M., Maitland-van der Zee A.-H., Tavendale R., Burchard E., Mukhopadhyay S., Palmer C. (2016). Risk for asthma exacerbations in association with variations in the genes coding for the glutathione S-transferase family. Eur. Respir. J..

[236] Barreto M., Villa M.P., Olita C., Martella S., Ciabattoni G., Montuschi P. (2009). 8-Isoprostane in exhaled breath condensate and exercise-induced bronchoconstriction in asthmatic children and adolescents. Chest.

[237] Shaheen S.O., Newson R.B., Rayman M.P., Wong A.P., Tumilty M.K., Phillips J.M., Potts J.F., Kelly F.J., White P.T., Burney P.G. (2007). Randomised, double blind, placebo-controlled trial of selenium supplementation in adult asthma. Thorax.

[238] Voicehovska J.G., Shkesters A., Orlikov G.A., Silova A.A., Rusakova N.J., Larmane L.T., Karpov J.G., Ivanov A.D., Maulinsh E. (2008). Assessment of some oxidative stress parameters in patients with bronchial asthma after selenium supplementation. Biochemistry. (Mosc.) Suppl. Ser. Part A Membr. Cell. Biol..

[239] Patil P., Patil P.B. (2020). Effect of selenium supplementation in bronchial asthma in children aged 1–12 years. Int. J. Sci. Res..

[240] Stolz D., Matera M.G., Rogliani P., van den Berge M., Papakonstantinou E., Gosens R., Singh D., Hanania N., Cazzola M., Maitland-van der Zee A.H. (2023). Current and future developments in the pharmacology of asthma and COPD: ERS seminar, Naples 2022. Breathe.

[241] Dato S., Crocco P., D’Aquila P., de Rango F., Bellizzi D., Rose G., Passarino G. (2013). Exploring the role of genetic variability and lifestyle in oxidative stress response for healthy aging and longevity. Int. J. Mol. Sci..

[242] Nadeem A., Raj H.G., Chhabra S.K. (2005). Increased oxidative stress in acute exacerbations of asthma. J. Asthma.

[243] Louhelainen N., Myllarniemi M., Rahman I., Kinnula V.L. (2008). Airway biomarkers of the oxidant burden in asthma and chronic obstructive pulmonary disease: Current and future perspectives. Int. J. Chron. Obstruct. Pulmon. Dis..

[244] Kauffmann F., Demenais F. (2012). Gene-environment interactions in asthma and allergic diseases: Challenges and perspectives. J. Allergy Clin. Immunol..

[245] Fabian E., Poloskey P., Kosa L., Elmadfa I., Rethy L.A. (2011). Activities of antioxidant enzymes in relation to oxidative and nitrosative challenges in childhood asthma. J. Asthma.

[246] Ohadike Y.U., Malveaux F.J., Lesch J.K. (2011). Challenges and lessons learned from the translation of evidence-based childhood asthma interventions: A commentary on the MCAN initiative. Health Promot. Pract..

[247] Arora N.S., Zhou S., Baptist A.P. (2024). Regulatory and insurance challenges must be overcome in the United States to meet global standards for asthma management. J. Allergy Clin. Immunol. Pract..

[248] Zhu L., Zhu Q., Zhang X., Wang H. (2013). The correlation analysis of two common polymorphisms in STAT6 gene and the risk of asthma: A meta-analysis. PLoS ONE.

[249] Greene L.S. (1995). Asthma and oxidant stress: Nutritional, environmental, and genetic risk factors. J. Am. Coll. Nutr..

[250] Yucesoy B., Johnson V.J., Lummus Z.L., Kissling G.E., Fluharty K., Gautrin D., Malo J.L., Cartier A., Boulet L.P., Sastre J. (2012). Genetic variants in antioxidant genes are associated with diisocyanate-induced asthma. Toxicol. Sci..

[251] Spiteri M., Bianco A., Strange R., Fryer A. (2000). Polymorphisms at the glutathione S-transferase, GSTP1 locus: A novel mechanism for susceptibility and development of atopic airway inflammation. Allergy.

[252] Agache I., Akdis C.A. (2019). Precision medicine and phenotypes, endotypes, genotypes, regiotypes, and theratypes of allergic diseases. J. Clin. Investig..

[253] Dani C., Poggi C. (2014). The role of genetic polymorphisms in antioxidant enzymes and potential antioxidant therapies in neonatal lung disease. Antioxid. Redox Signal..

[254] di Palmo E., Cantarelli E., Catelli A., Ricci G., Gallucci M., Miniaci A., Pession A. (2021). The Predictive Role of Biomarkers and Genetics in Childhood Asthma Exacerbations. Int. J. Mol. Sci..

[255] Wang L., Xie J., Hu Y., Tian Y. (2022). Air pollution and risk of chronic obstructed pulmonary disease: The modifying effect of genetic susceptibility and lifestyle. eBioMedicine.

[256] Kholmirzayev B. (2025). Genetic Insights into Asthma Pathogenesis and Therapeutic Approaches. Front. Glob. Sci..

[257] Liu P.-J., Lin K.-P., Chen P.-C. (2012). Nutritional supplement therapy improves oxidative stress, immune response, pulmonary function, and quality of life in allergic asthma patients: An open-label pilot study. Altern. Med. Rev..

[258] Schleich F., Demarche S., Louis R. (2016). Biomarkers in the Management of Difficult Asthma. Curr. Top. Med. Chem..

[259] Zajac D., Wojciechowski P. (2023). The Role of Vitamins in the Pathogenesis of Asthma. Int. J. Mol. Sci..

[260] Jacobson G.A., Yee K.C., Ng C.H. (2007). Elevated plasma glutathione peroxidase concentration in acute severe asthma: Comparison with plasma glutathione peroxidase activity, selenium and malondialdehyde. Scand. J. Clin. Lab. Investig..

[261] Aleksandrova K., Koelman L., Rodrigues C.E. (2021). Dietary patterns and biomarkers of oxidative stress and inflammation: A systematic review of observational and intervention studies. Redox Biol..

[262] Krishnamurthy H.K., Rajavelu I., Pereira M., Jayaraman V., Krishna K., Wang T., Bei K., Rajasekaran J.J. (2024). Inside the genome: Understanding genetic influences on oxidative stress. Front. Genet..

[263] Papamichael M.M., Katsardis C., Sarandi E., Georgaki S., Frima E.S., Varvarigou A., Tsoukalas D. (2021). Application of Metabolomics in Pediatric Asthma: Prediction, Diagnosis and Personalized Treatment. Metabolites.

[264] Gupta S., Venkatesh A., Ray S., Srivastava S. (2014). Challenges and prospects for biomarker research: A current perspective from the developing world. Biochim. Biophys. Acta.

